# Supplementary material for: Enantiopure trigonal bipyramidal coordination cages templated by in situ self-organized D2h-symmetric anions
Source: Nat Commun. 2024 Jul 4;15:5628. doi: 10.1038/s41467-024-49964-w (PMC11224320; doi:10.1038/s41467-024-49964-w)
Supplement: Supplementary file 1 — Supplementary Information [file 41467_2024_49964_MOESM1_ESM.pdf]

Supplementary Information for

Enantiopure trigonal bipyramidal coordination cages templated by in situ self-organized D_{2h}-symmetric anions

Shan Guo[1]#, Wen-Wen Zhan[1]#, Feng-Lei Yang[1]#, Jie Zhou[1], Yu-Hao Duan[1], Dawei Zhang[2]* and Yang Yang[1]*

[1]School of Chemistry and Materials Science, Jiangsu Normal University, Xuzhou 221116, China

*E-mail: yangyang@jsnu.edu.cn

[2]State Key Laboratory of Petroleum Molecular & Process Engineering, Shanghai Key Laboratory of Green Chemistry and Chemical Processes, School of Chemistry and Molecular Engineering, East China Normal University, Shanghai 200062, China.

*Email: dwzhang@chem.ecnu.edu.cn

[#]These authors contribute equally.

Table of Contents

1. Materials and instrumentation 3

2. Synthesis and characterization for ligand 4

2.1 Synthesis of ligand $\mathbf{L^2}$ 4

2.2 Synthesis precursor ligand **B** 6

2.3 Synthesis of precursor ligand **C** 8

3. Synthesis and characterization for cages 10

3.1 Synthesis of cage **1** 10

3.2 Synthesis of cage **2-Cr** 14

3.3 Synthesis of cage **2-Zn** 17

3.4 Transforming **1** to **2-Zn** 22

3.5 Attempts to induce cages by using 2, 2′-bipyrimdine or $[Ru(2,\ 2'\text{-bipyrimdine})_3]^{2+}$ 25

3.6 Synthesis of cage **3** 28

3.7 Synthesis of cage **4** 32

3.8 Synthesis of cage **5** 36

3.9 Synthesis of cage **6-Zn** 38

3.10 Synthesis of cage **6-Co** 43

3.11 Synthesis of cage $\mathbf{6\text{-}Zn\text{-}O_2}$ 45

4. Density functional theory (DFT) calculations 50

5. Release of singlet oxygen 52

6. Photophysical properties 54

7. Crystallographic data and structure refinement 58

References: 69

1. Materials and instrumentation

Unless otherwise specified, all reagents were purchased from commercial sources and used as received. Precursor ligand **A** was purchased form Bidepharm. Caution: *Perchlorate salts are potentially explosive*.

NMR spectra were recorded using Bruker Avance III 101 MHz (^{13}C NMR), Bruker Avance III 400 MHz (^{1}H NMR, COSY) in deuterated deuterium reagent and are reported relative to residual solvent signals. Chemical shifts for ^{1}H and ^{13}C NMR are reported in ppm on the δ scale;^{1}H and ^{13}C were referenced to the residual solvent peak. Coupling constants (J) are reported in Hz. The assignments were done with the help of ^{1}H-^{1}H COSY experiments.

Mass spectra (MS) were recorded on a Bruker impact II high definition mass spectrometer, quadrupole and time-of-flight (Q/TOF) modules. Typical measurement conditions are as follows: end plate offset = -400 V; dry gas = 3 L min^{-1}, nebulizer = 0.3 bar, capillary voltage = 3500 V, sample flow rate = 180 μL/h. The data analyses of mass spectra were performed based on the isotope distribution patterns using Compass Data Analysis software (Version 4.4). Unless otherwise specified, the samples were prepared by dissolving the crystals of the products.

Circular Dichroism was performed on an JASCO J-810 CD spectrometer using a 1 cm path-length cuvette. Experiments were recorded at 298 K. Measurements were background subtracted from blank solvent in an identical cuvette. The sample concentrations were adjusted to maintain a HV below 600 V. A minimum sample integration time of 1 second was used.

The excitation and emission spectra were obtained by Hitachi F4700. The emission lifetimes were determined using an Edinburgh FLS1000 spectrometer and absolute quantum yields were measured by Hamamatsu PMA-12 with integrating sphere. UV-Vis spectra were measured using a Shimadzu 2550 spectrometer. The solid state samples were the crystalline products. The solution samples were prepared by dissolving 2 mg crystalline product in 3 mL acetonitrile. Jasco CPL-300 was used to measure the circularly polarized luminescence.

X-ray photoelectron spectroscopy (XPS) was recorded on Thermo Fisher K-Alpha spectrometer. The sample was the crystals.

2. Synthesis and characterization for ligand

2.1 Synthesis of ligand L^2

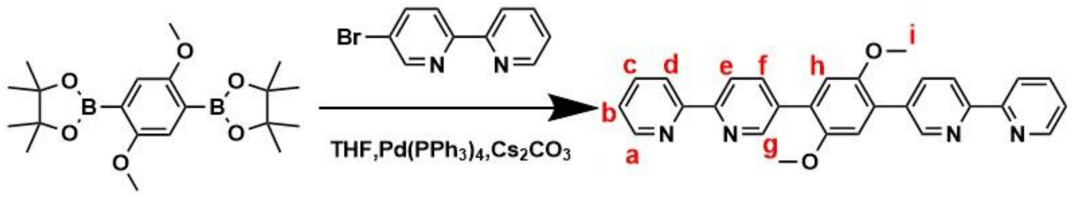

The $\mathbf{L^2}$ ligand was synthesized based on reported methods. A suspension of Cs_2CO_3 (1.74 g, 5.34 mmol), 5-bromo-2,2'-bipyridine (1.44 g, 6.10 mmol) and 2,2'-(2,5-dimethoxy-1,4-phenylene)-bis(4,4,5,5-tetramethyl-1,3,2-dioxaborolane) (1 g, 2.56 mmol) were mixed in THF (200 mL) and degassed with nitrogen for 15 minutes. $Pd(PPh_3)_4$ (500 mg, 0.432 mmol) was added and the mixture was heated at 74 °C for 72 hours under nitrogen. After cooling to room temperature, the mixture was extracted with 100 mL ethyl acetate 3 times. The combined organic layers were washed with brine, and dried over $MgSO_4$ and the solvent was evaporated. The crude product was purified by flash column, with mixed solvent hexane/ethyl acetate (1:1) as eluent. About 1.0 g of ligand $\mathbf{L^2}$ was obtained. Yield: 87%. ^{1}H NMR (400 MHz, $CDCl_3$) δ 8.93 (s, 1H), 8.71 (d, J = 4.8 Hz, 1H), 8.47 (t, J = 8.9 Hz, 2H), 8.11 - 8.04 (d, J = 10.4 Hz, 1H), 7.81 (dd, J_1= 5.2 Hz, J_2=4.0 Hz, 1H), 7.33 (dd, J_1= 7.2 Hz, J_2=5.1 Hz, 2H), 7.08 (s, 1H), 3.86 (s, 3H).^{13}C NMR (100 MHz, $CDCl_3$) δ 156.08, 154.75, 151.19, 149.68, 149.36, 137.74, 137.05, 133.90, 127.63, 123.80, 121.17, 120.53, 114.35, 56.55. ESI-MS (positive mode, CH_3CN/ CH_3OH, m/z, high resolution): calculated for $(C_{28}H_{22}N_4O_4+H)^+$: 447.17, found: 447.18.

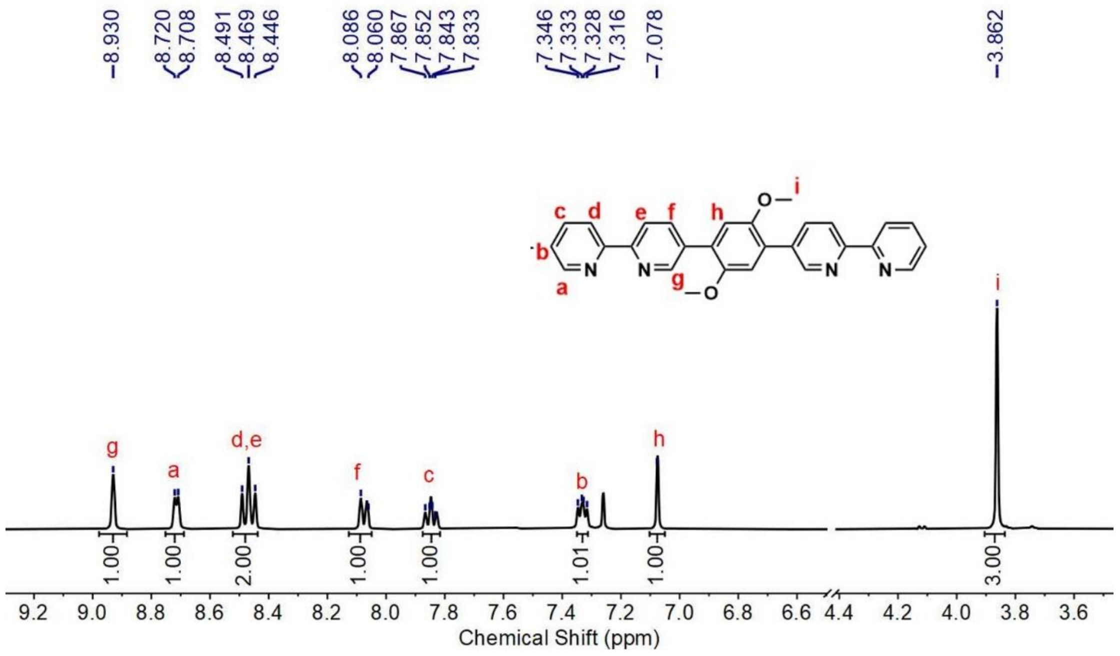

Supplementary Figure 1. ^{1}H NMR spectrum of $\mathbf{L^2}$ in $CDCl_3$ (400 MHz, 298 K).

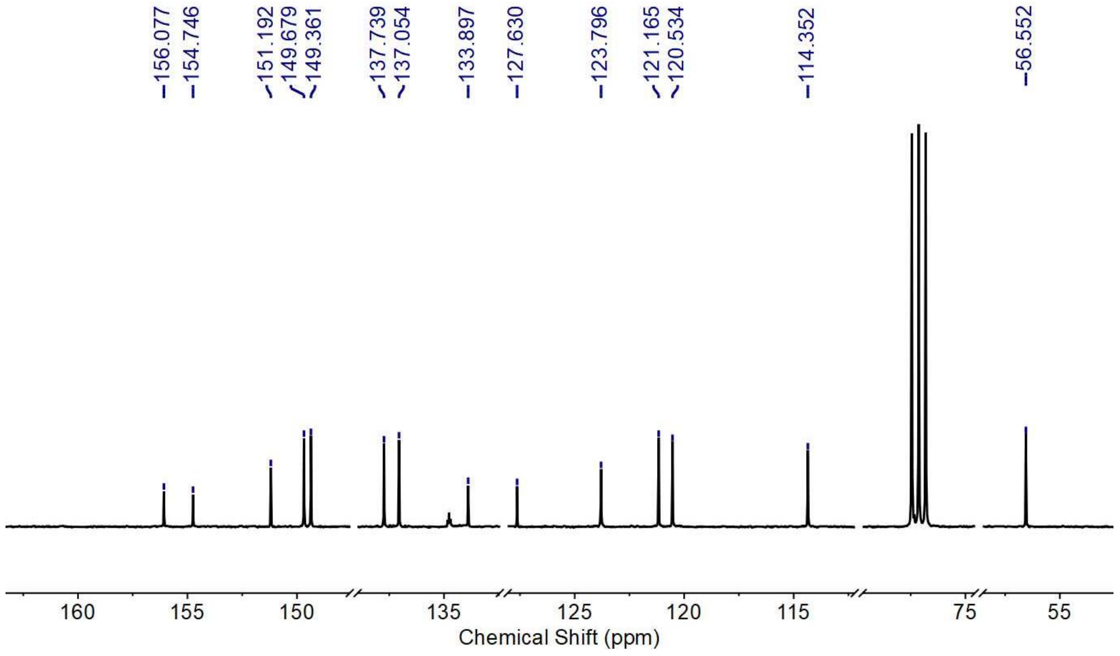

Supplementary Figure 2. ^{13}C NMR spectrum of $\mathbf{L^2}$ in $CDCl_3$ (100 MHz, 298 K).

2.2 Synthesis precursor ligand B

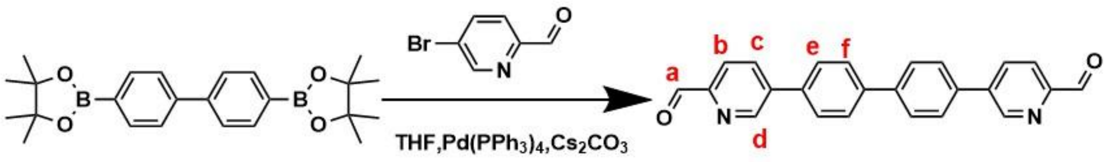

A suspension of Cs_2CO_3 (838 mg, 2.60 mmol), 5-bromopicolinaldehyde (547 mg, 2.90 mmol), and 4,4'-bis(4,4,5,5-tetramethyl-1,3,2-dioxaborolan-2-yl)-1,1'-biphenyl (500 mg, 1.23 mmol) was mixed in THF (200 mL) and degassed with nitrogen for 15 minutes. $Pd(PPh_3)_4$ (210 mg, 0.18 mmol) was added, and the mixture was heated at 72 °C for 72 hours under nitrogen. After cooling to room temperature, the mixture was extracted with 100 mL ethyl acetate for 3 times. The combined organic layers were washed with brine, and dried over $MgSO_4$. The solvent was evaporated and crude product was purified by flash column, with mixed solvent hexane/ethyl acetate (1:1) as eluent. About 350 mg of ligand **B** was obtained. Yield: 78%. ^{1}H NMR (400 MHz, $CDCl_3$) δ 10.15 (s, 1H), 9.09 (s, 1H), 8.13 (d, J = 8.3 Hz, 1H), 8.08 (d, J = 8.0 Hz, 1H), 7.82 (d, J = 8.0 Hz, 2H), 7.78 (d, J = 8.1 Hz, 2H). ^{13}C NMR (100 MHz, CD_2Cl_2) δ 193.10, 151.83, 148.62, 140.74, 139.93, 136.07, 135.04, 128.01, 127.91, 121.70. ESI-MS (positive mode, CH_3CN/CH_3OH, m/z, high resolution): calculated for $(C_{24}H_{16}N_2O_2+H)^+$: 365.12, found: 365.13.

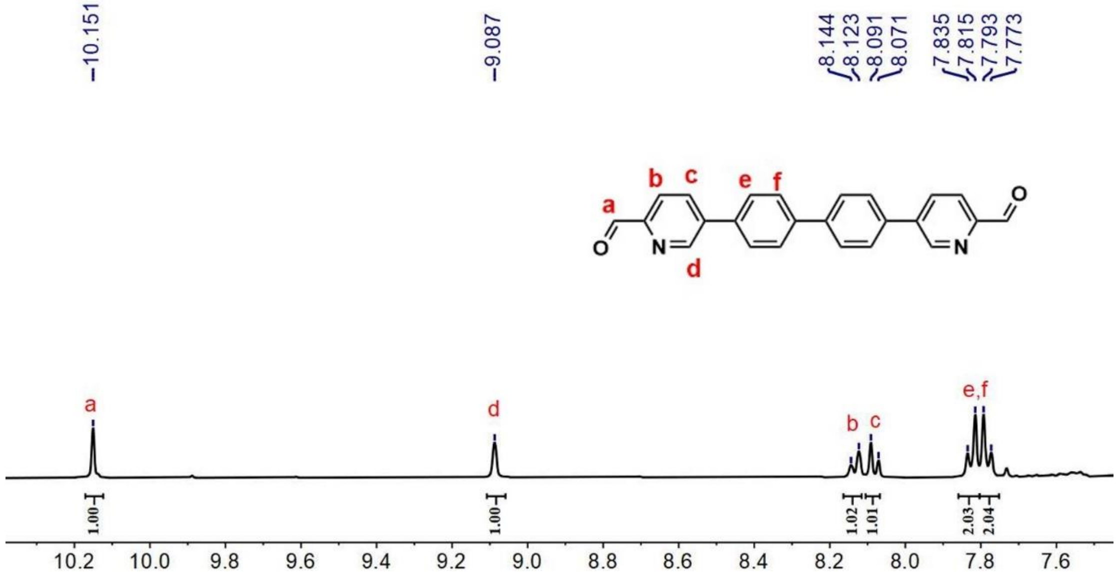

Supplementary Figure 3. ^{1}H NMR spectrum of **B** in $CDCl_3$ (400 MHz, 298 K).

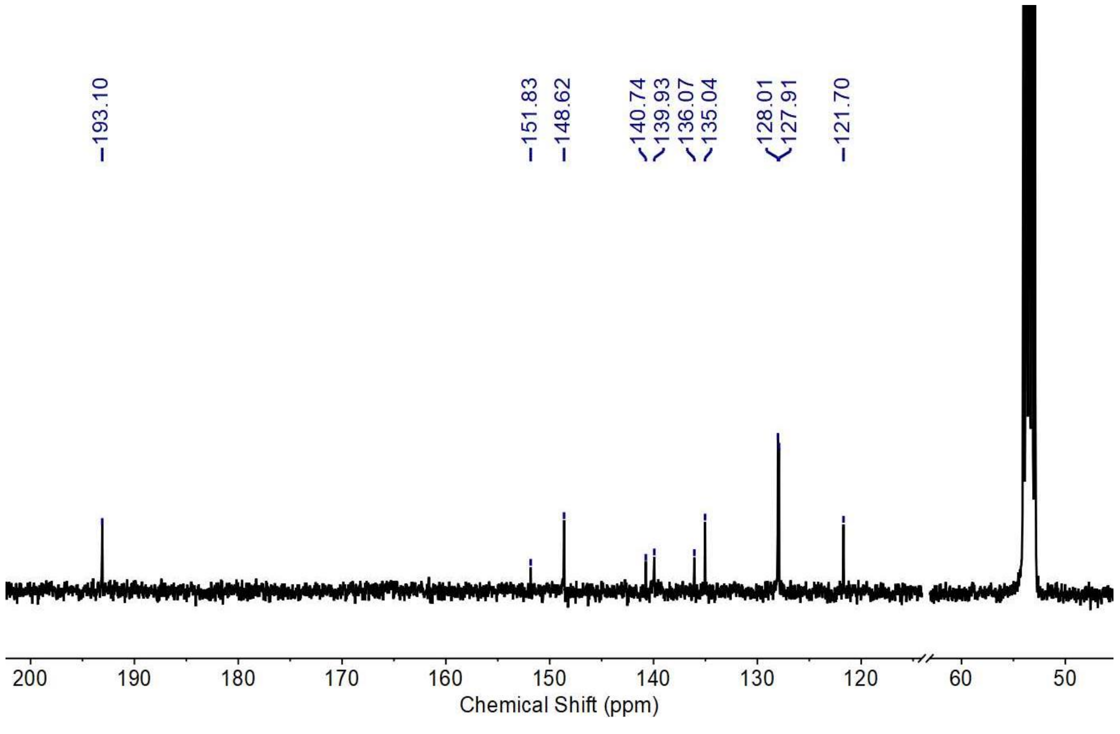

Supplementary Figure 4. ^{13}C NMR spectrum of **B** in CD_2Cl_2(100 MHz, 298 K).

2.3 Synthesis of precursor ligand C

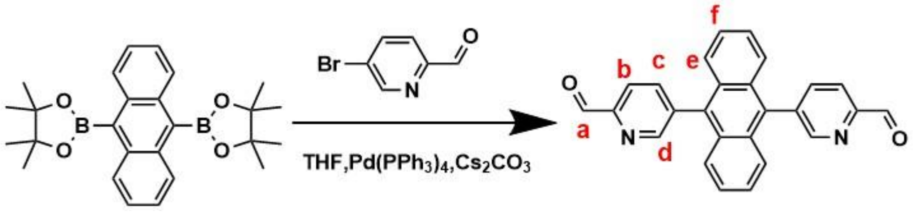

Cs_2CO_3 (4.4 g, 13.5 mmol), $Pd(PPh_3)_4$ (1.0 g, 0.87 mmol), 9,10-Anthracenediboronic acid bis(pinacol) ester (2.9 g, 6.7 mmol), and 5-bromo-2-formylpyridine (3.0 g, 16.1 mmol) were added stepwise into a 500 mL Schlenk flask. THF (200 mL) was poured into the flash to make a suspension, and then N_2 was pumped into the suspension for 5 minutes. The mixture was refluxed at 68 °C for 72 hours in a nitrogen atmosphere in the absence of light. The final solution was concentrated under reduced pressure to give a brown solid, which was purified by flash column chromatography on silica gel (EtOAc : hexane = 1:1) to afford 1.10g (42.3% yield) target product as a yellow powder.^{1}H NMR (400 MHz, $CDCl_3$, ppm): δ10.31 (s, 1H), 8.94 (s, 1H), 8.3 (d, J = 6.4 Hz, 1H), 8.08 (d, J = 6.4 Hz, 1H), 7.62 (q, J_1 = 5.2 Hz, J_2 = 2.4 Hz, 2H), 7.47 (q, J_1 = 5.6Hz, J_2 = 2.8Hz, 2H). ^{13}C NMR (100 MHz, $CDCl_3$, ppm): δ 192.08, 151.22, 138.95, 138.30, 131.99, 128.81, 125.41, 125.10, 120.49. ESI-MS (positive mode, CH_3CN/CH_3OH, m/z, high resolution): calculated for $(C_{26}H_{16}N_2O_2+H)^+$: 389.12, found: 389.13.

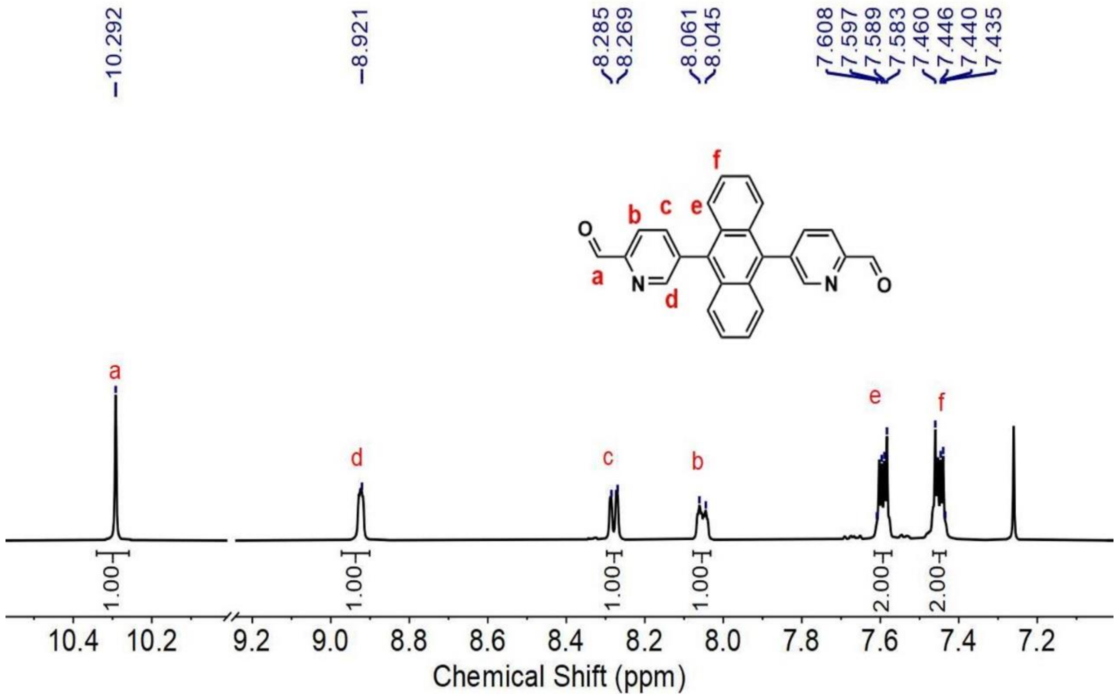

Supplementary Figure 5. ^{1}H NMR spectrum of **C** in $CDCl_3$ (400 MHz, 298 K).

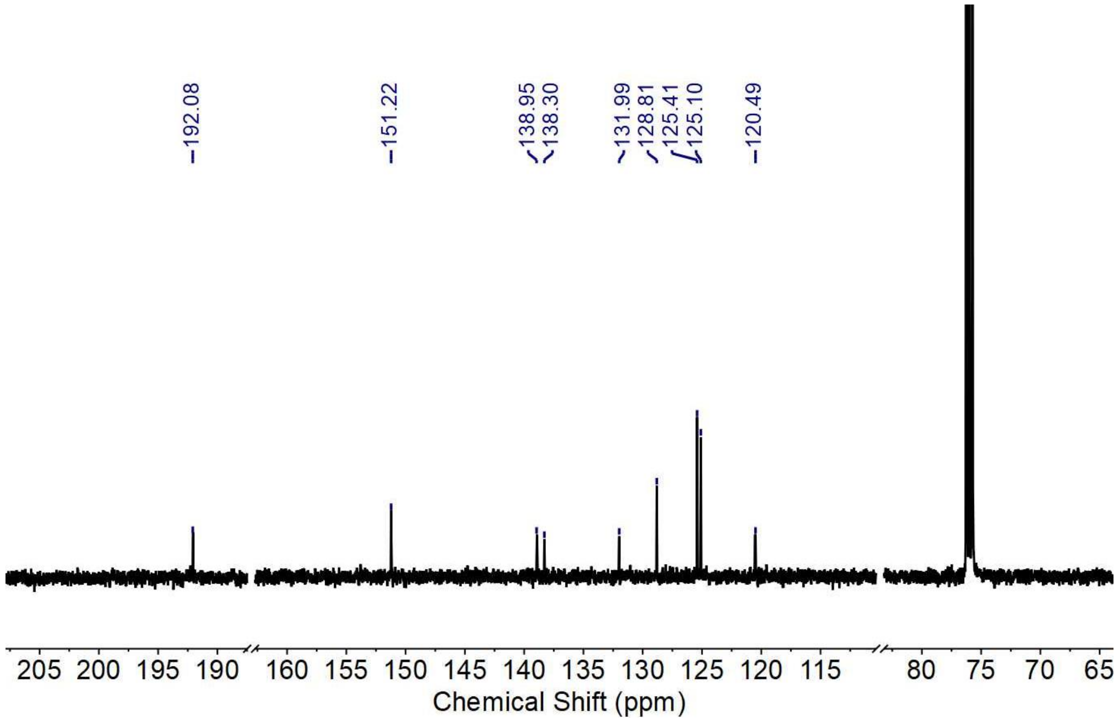

Supplementary Figure 6. ^{13}C NMR spectrum of **C** in $CDCl_3$ (100 MHz, 298 K).

3. Synthesis and characterization for cages

3.1 Synthesis of cage 1

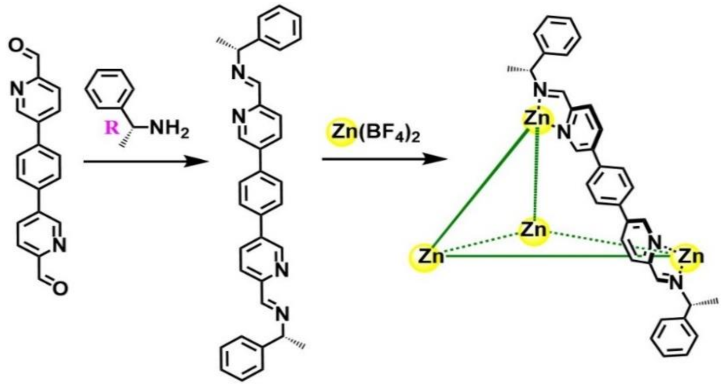

Subcomponent **A** (10 mg, 34.7 µmol) and *R*-1-phenylethylamine (9 µL, 69.4 µmol) were dissolved in 2 mL acetonitrile. The mixed solution was stirred at 68 °C for 4 hours. After cooling to room temperature, zinc tetrafluoroborate hydrate (8.2 mg,23.6 µmol) was added. The mixture was stirred at room temperature overnight. The resulted solution was filtered into a thin tube, and anhydrous diethyl ether was layered onto the solution. The diffusion was allowed to obtain crystals of *R*-**1**. About 20.48 mg of cage *R*-**1** was obtained, yield: 88%. *S*-**1** was obtained by replacing *R*-1-phenylethylamine with *S*-1-phenylethylamine. ^{1}H NMR (400 MHz, CD_3CN) δ 8.37 (s, 1H), 8.27 (d, *J* = 10.0 Hz, 1H), 7.72 (s, 1H), 7.56 (d, *J* = 8.1 Hz, 1H), 7.42 (s, 2H), 7.11 (dd, J_1 = 7.2 Hz, J_2 = 7.6 Hz, 1H), 6.94 (dd, J_1 = 7.6 Hz, J_2 = 7.2 Hz, 2H), 6.76 (d, *J* = 7.6 Hz, 2H), 5.46 (q, J_1 = 6.3 Hz, J_2 = 6.3 Hz, 1H), 1.68 (d, *J* = 6.5 Hz, 3H). ^{13}C NMR (100 MHz, CD_3CN) δ 161.72, 145.85, 145.27, 140.60, 139.90, 139.34, 135.78, 129.91, 128.89, 127.88, 127.85, 125.74, 64.28, 22.91. The counterions (perchlorate, bis-(trifluoromethylsulfonyl)imide and tetrafluoroborate ions) of zinc salts did not affect the formation of the target cages. ESI-MS (positive mode, CH_3CN, m/z, high resolution): calculated for $[(\mathbf{L^1})_6Zn_4(ClO_4)_5]^{3+}$: 1242.20, found: 1241.93; calculated for $[(\mathbf{L^1})_6Zn_4(ClO_4)_4]^{4+}$: 906.79, found: 906.19.

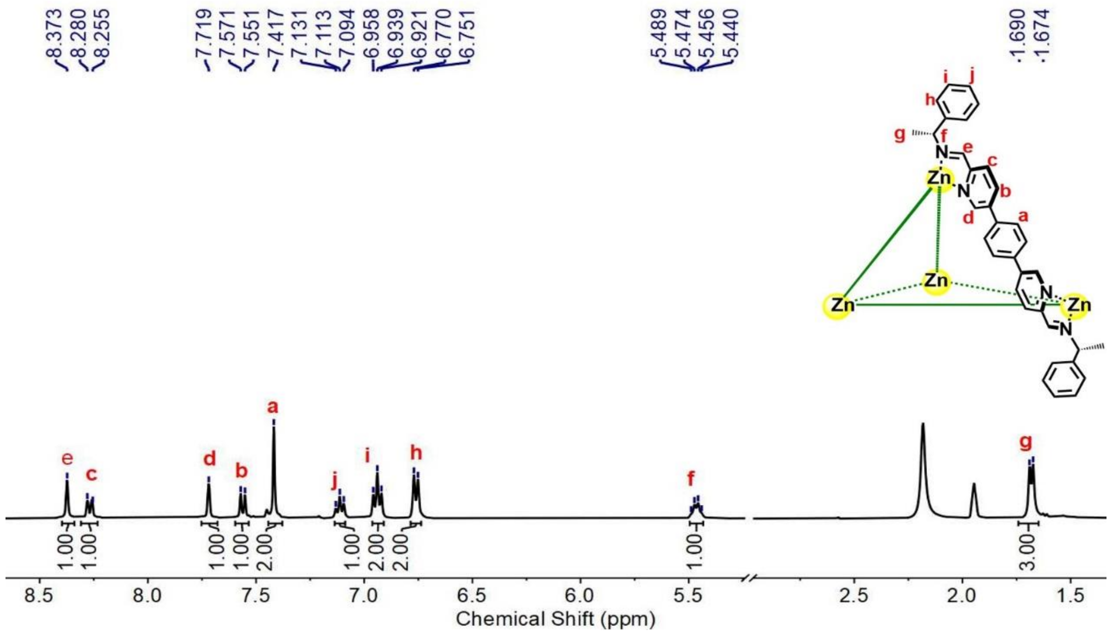

Supplementary Figure 7. ^{1}H NMR spectrum of **1** in CD_3CN (400 MHz, 298 K).

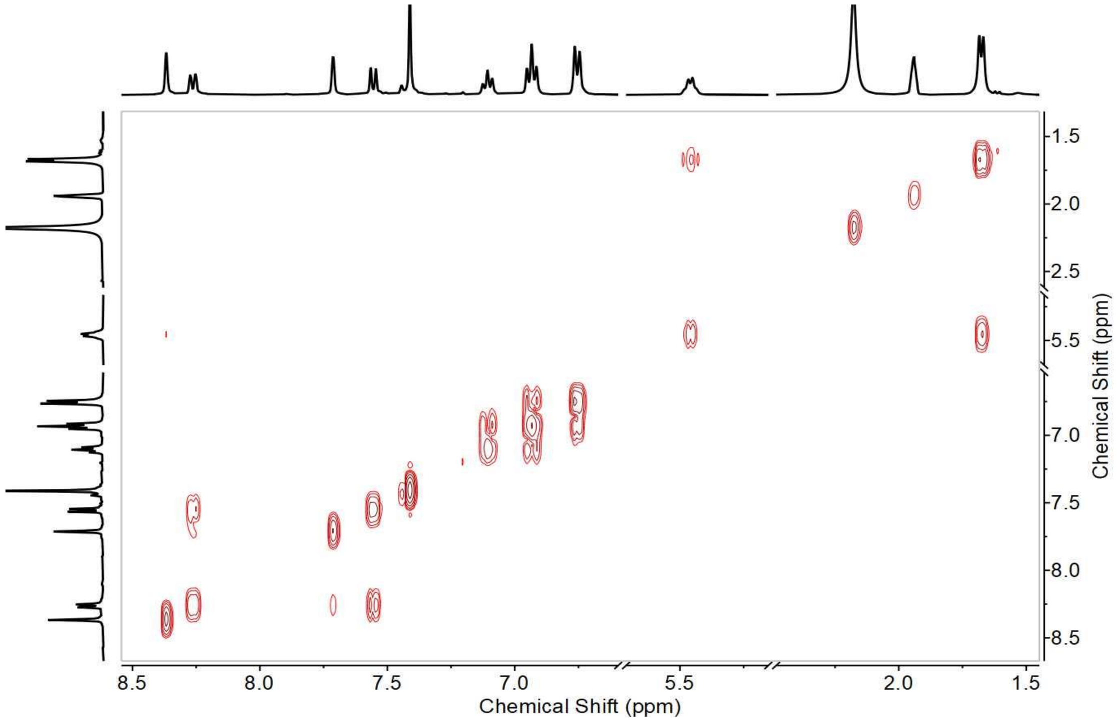

Supplementary Figure 8. ^{1}H-^{1}H COSY spectrum of **1** in CD_3CN (298 K).

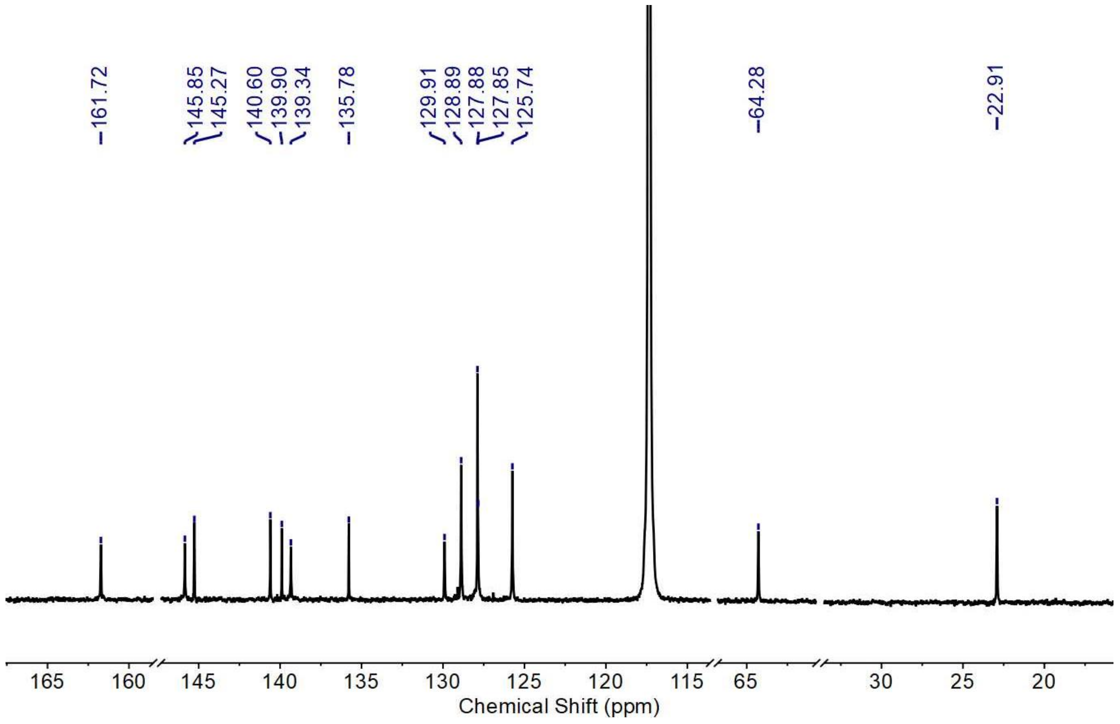

Supplementary Figure 9. ^{13}C NMR spectrum of **1** in CD_3CN (100 MHz, 298 K).

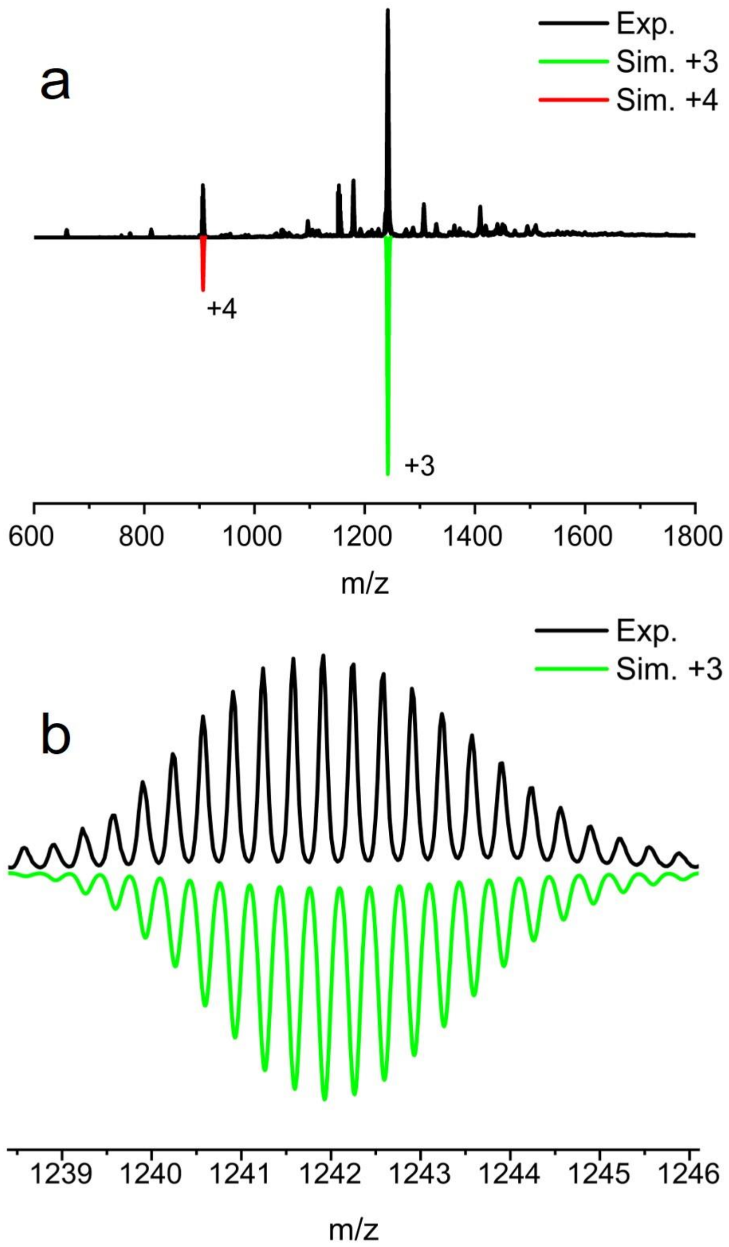

Supplementary Figure 10. a) ESI-MS spectrum of **1** in acetonitrile solution; b) experimental and simulated isotopic patterns of 3+ charged molecular peak of **1** by losing the corresponding numbers of counter-ions.

3.2 Synthesis of cage 2-Cr

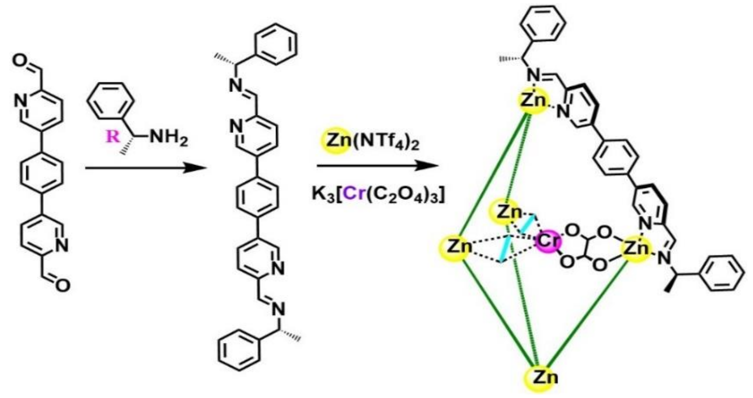

Subcomponent **A** (10 mg, 34.7 μmol) and *R*-1-phenylethylamine (9 μL, 69.4 μmol) were dissolved in 2 mL acetonitrile. The mixture was stirred at 68 °C for 4 hours. After cooling to room temperature, zinc bis-(trifluoromethylsulfonyl)imide (18.08 mg, 28.9 μmol) and $K_3[Cr^{III}(C_2O_4)_3]$ (2.8 mg, 5.8 μmol) dissolving in 30 μL water were added into the above solution. The mixture was further stirred at room temperature overnight. The resulting solution was filtered into a thin tube, and anhydrous diethyl ether was layered onto the solution. The diffusion led to crystals of *R*-**2-Cr**, yield: 90%. *S*-**2-Cr** was obtained by replacing *R*-1-phenylethylamine with *S*-1-phenylethylamine. The counterions (perchlorate, bis-(trifluoromethylsulfonyl)imide and tetrafluoroborate ions) of zinc salts did not affect the formation of the target cages. ESI-MS (positive mode, CH_3CN, m/z, high resolution): calculated for $\{(\mathbf{L^1})_6Zn_5[Cr(C_2O_4)_3](C_2NS_2O_4F_6)_4\}^{3+}$: 1576.89, found: 1576.78; calculated for $\{(\mathbf{L^1})_6Zn_5[Cr(C_2O_4)_3](C_2NS_2O_4F_6)_3\}^{4+}$: 1112.69, found: 1112.62.

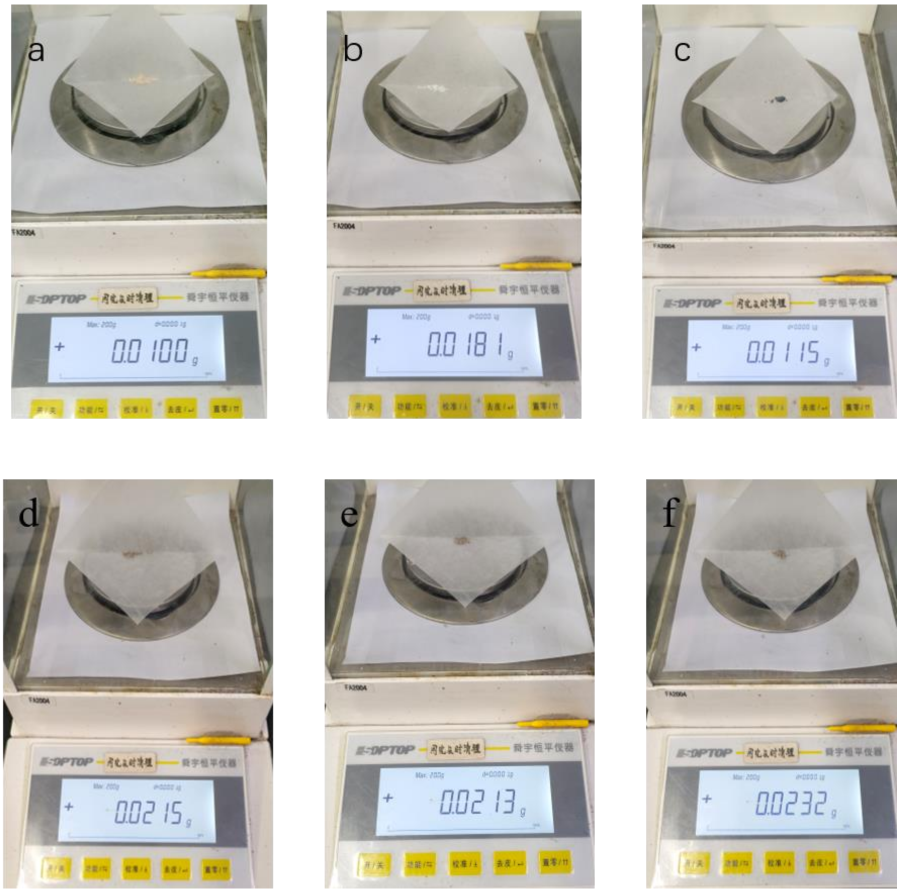

Supplementary Figure 11. The weights of materials using for the preparation of **2-Cr**: a) ligand **A**; b) zinc bis-(trifluoromethylsulfonyl)imide; c) $K_3[Cr^{III}(C_2O_4)_3]$; and d - f) crystalline products of **2-Cr** from three independent experiments. The crystalline product weighted in the range of 21.3 mg - 23.2 mg with an average value of 22.0 mg, corresponding to a 70% yield.

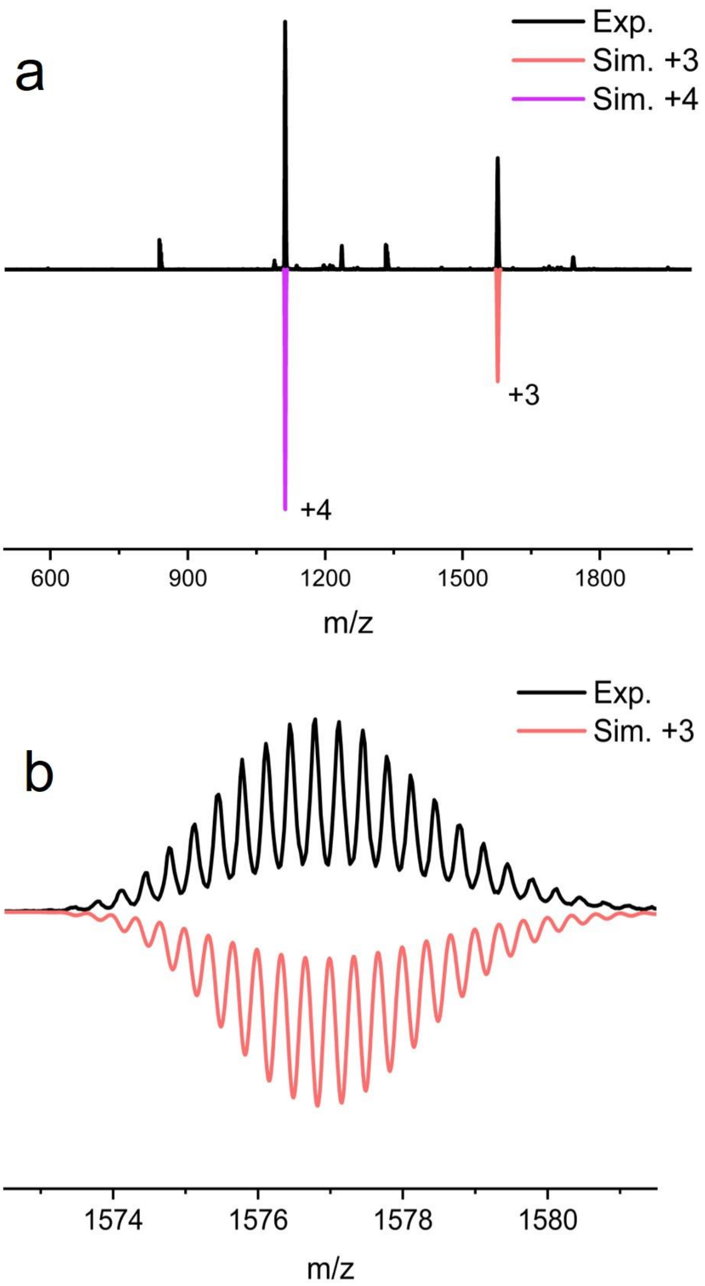

Supplementary Figure 12. a) ESI-MS spectrum of **2-Cr** in acetonitrile solution, b) experimental and simulated isotopic patterns of 3+ charged molecular peak of **2-Cr** by losing the corresponding numbers of counter-ions.

3.3 Synthesis of cage 2-Zn

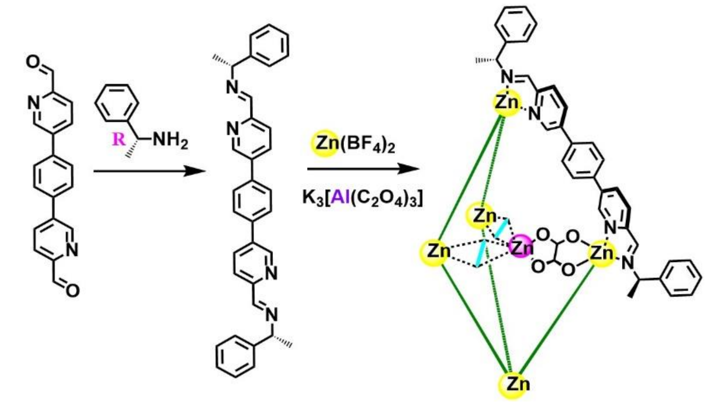

Subcomponent **A** (10 mg, 34.7 μmol) and *R*-1-phenylethylamine (9 μL, 69.4 μmol) were dissolved in 2 mL acetonitrile. The mixture was stirred at 68 °C for 4 hours. After cooling to room temperature, zinc tetrafluoroborate hydrate (12 mg, 34.7 μmol) and $K_2C_2O_4$(3.2 mg, 17.3 μmol) which was dissolved in 30 μL water were added into the above solution. The mixture was stirred at room temperature overnight. The resulting solution was filtered into a thin tube, and anhydrous diethyl ether was layered onto the solution. The slow diffusion led to crystals of *R*-**2-Zn**. About 22.8 mg of crystalline cage *R*-**2-Zn** was obtained, yield: 92%. *S*-**2-Zn** was obtained by replacing *R*-1-phenylethylamine with *S*-1-phenylethylamine. The counterions (perchlorate, bis-(trifluoromethylsulfonyl)imide and tetrafluoroborate ions) of zinc salts did not affect the formation of the target cages. ^{1}H NMR (400 MHz, CD_3CN) δ 8.45 (d, J = 8.8 Hz, 2H), 8.40 (s, 2H), 8.31 (s, 1H), 8.18 (s, 1H), 7.91 (d, J = 8.4 Hz, 2H), 7.80 (d, J= 8.0 Hz, 1H), 7.66 (d, J = 8.0 Hz, 2H), 7.47 (d, J = 8.0 Hz, 1H), 7.18 (dd, J_1= 7.2 Hz, J_2= 7.6 Hz, 1H), 7.02 (dd, J_1= 7.6 Hz, J_2= 7.6 Hz, 2H), 6.87-6.82 (m, 3H), 6.76 (d, J = 7.6 Hz, 2H), 6.72 (d, J = 8.4 Hz, 2H), 5.53 (q, J_1= 6.4 Hz, J_2= 6.4 Hz, 1H), 4.79 (q, J_1= 6.8 Hz, J_2= 6.8 Hz, 1H), 1.74 (d, J = 6.4 Hz, 3H), 1.30 (d, J = 6.4 Hz, 3H). ^{13}C NMR (100 MHz, CD_3CN) δ 168.26, 163.04, 161.24, 147.55, 147.38, 146.75, 146.44, 141.72, 140.40, 140.22, 139.87, 139.52, 136.79, 136.67, 130.91, 129.94, 129.64, 129.28, 129.19, 128.75, 128.65, 128.30, 127.90, 126.49, 122.45, 119.26, 116.07, 66.19, 65.80, 65.10, 23.92, 20.95. ESI-MS (positive mode, CH_3CN, m/z, high resolution): calculated for $\{(\mathbf{L^1})_6Zn_5[Zn(C_2O_4)_3](BF_4)_3\}^{3+}$: 1294.87, found: 1294.73; calculated for $\{(\mathbf{L^1})_6Zn_5[Zn(C_2O_4)_3](BF_4)_2\}^{4+}$: 949.45, found: 949.33.

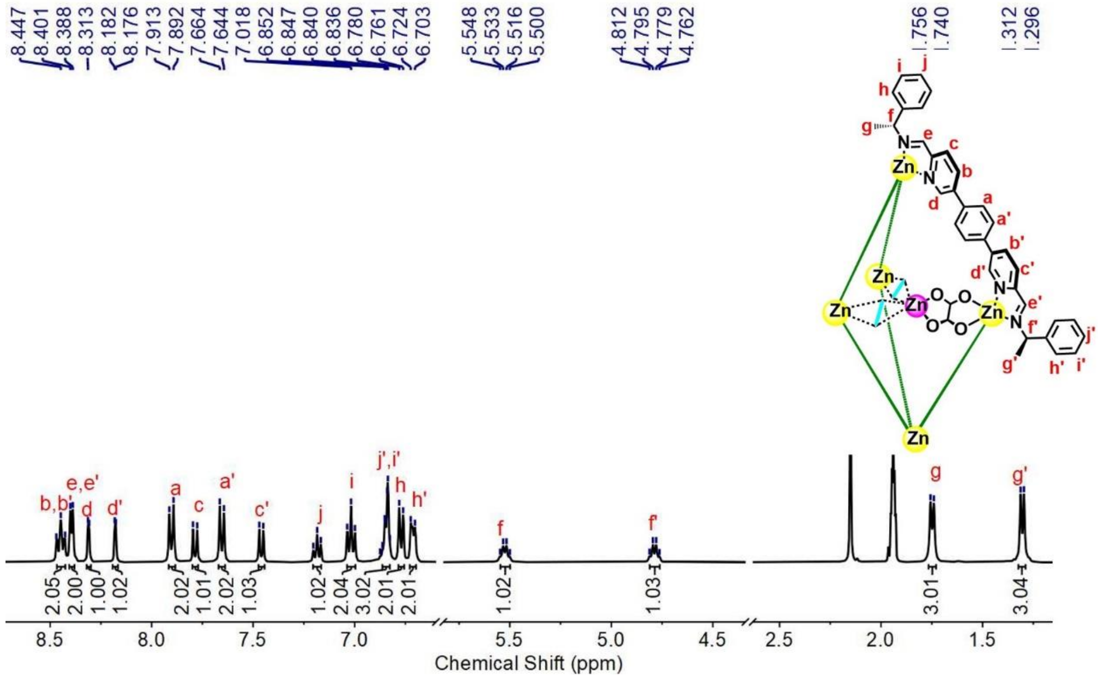

Supplementary Figure 13. ^{1}H NMR spectrum of **2-Zn** in CD_3CN (400 MHz, 298 K).

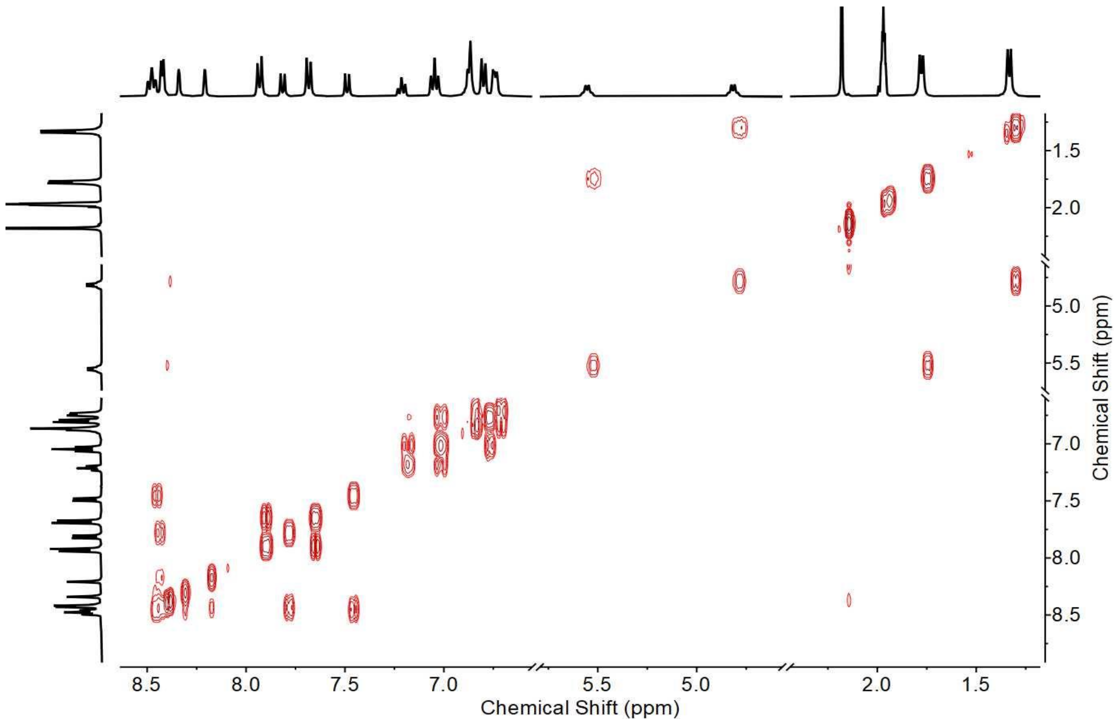

Supplementary Figure 14. ^{1}H-^{1}H COSY spectrum of **2-Zn** in CD_3CN (298 K).

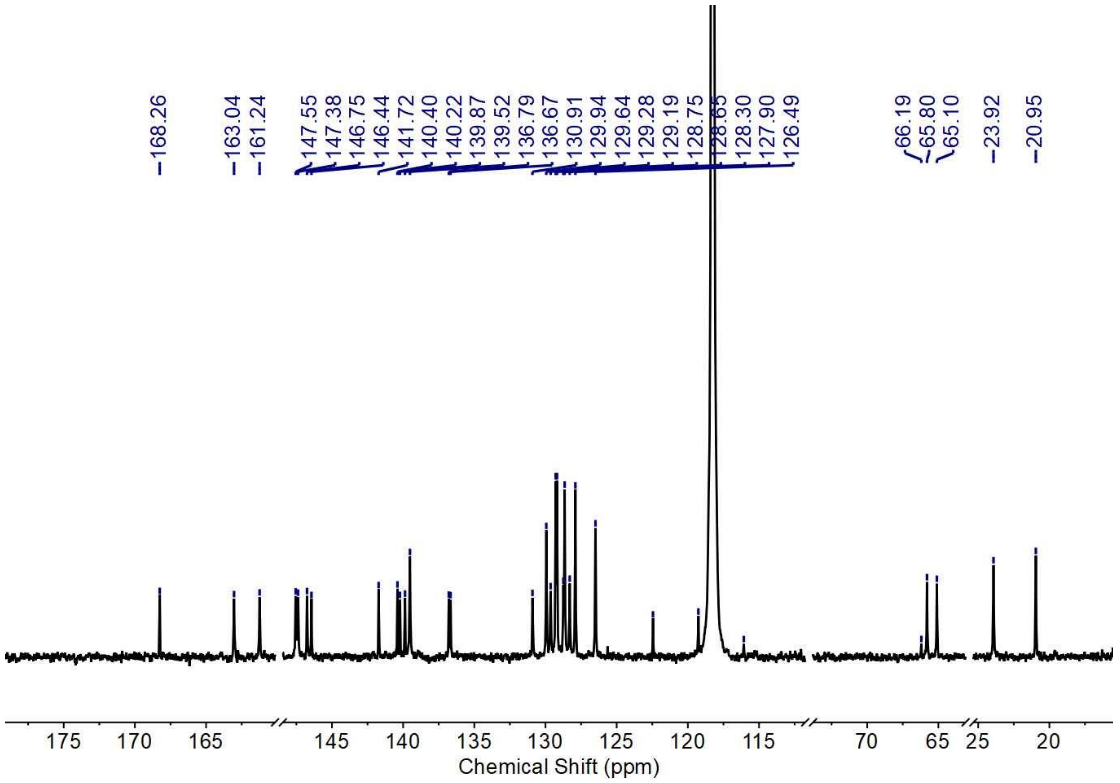

Supplementary Figure 15. ^{13}C NMR spectrum of **2-Zn** in CD_3CN (100 MHz, 298 K).

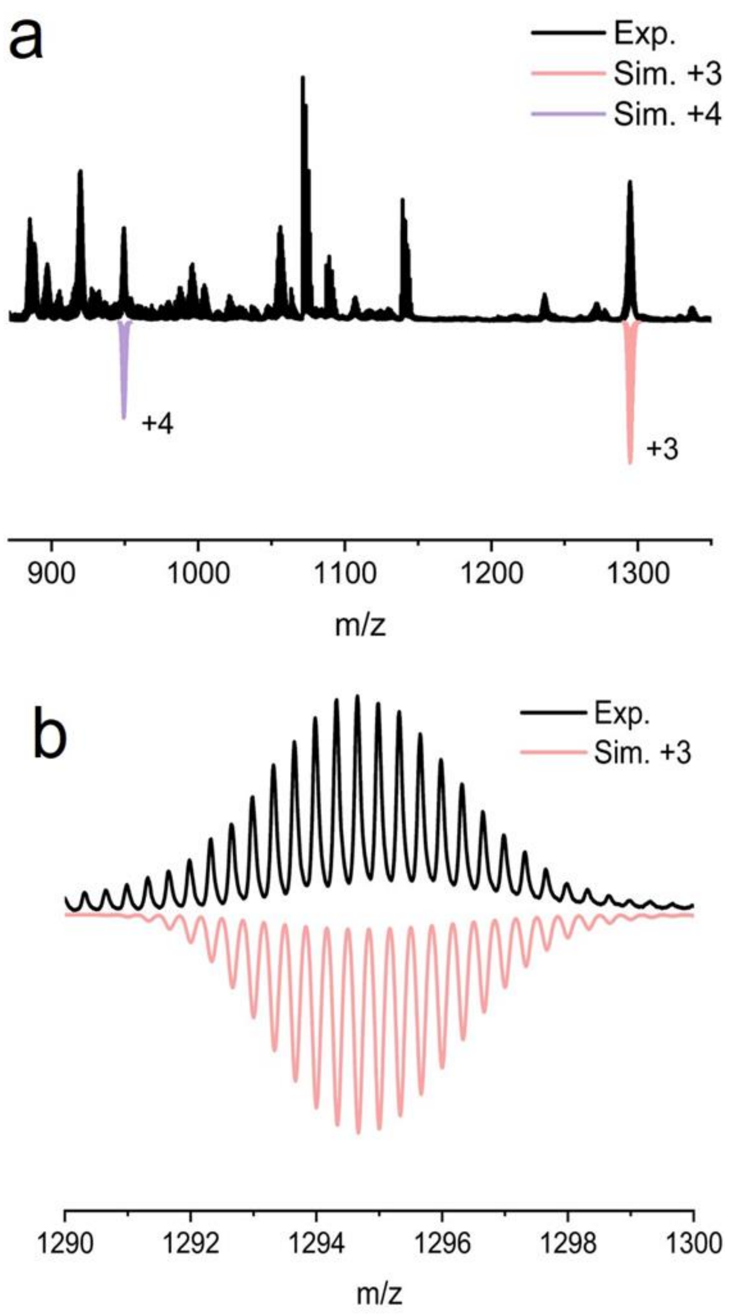

Supplementary Figure 16. a) ESI-MS spectrum of **2-Zn** in acetonitrile solution; b) experimental and simulated isotopic patterns of 4+ charged molecular peak of **2-Zn** by losing the corresponding numbers of counter-ions.

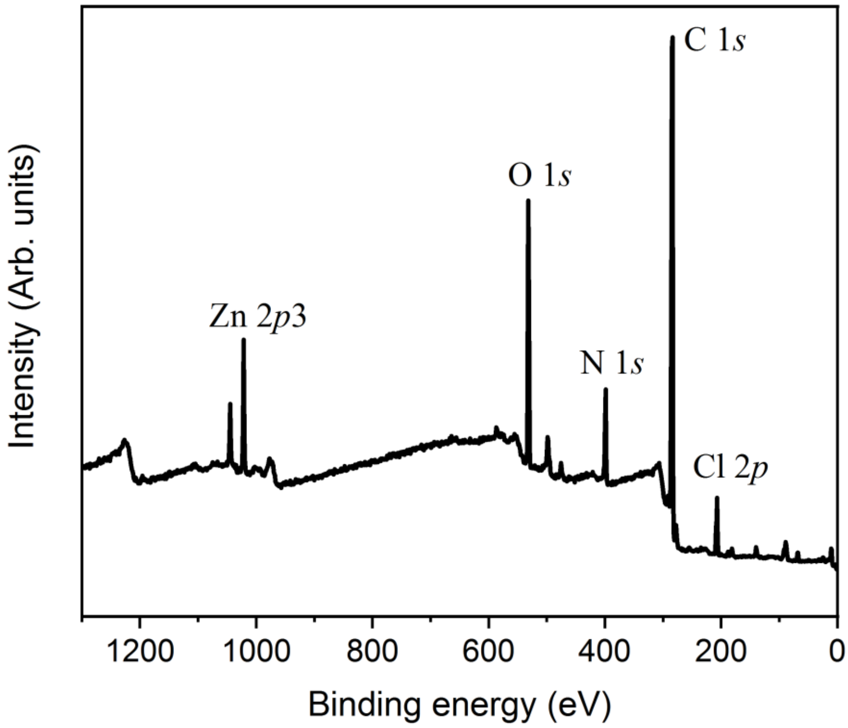

Supplementary Figure 17. XPS of **2-Zn** (The metal salt is zinc perchlorate hexahydrate).

3.4 Transforming 1 to 2-Zn

When additional 2 eq. $Zn(BF_4)_2$ and 3 eq. potassium oxalate were added into the solution of **1**, the **2-Zn** appear gradually. We try to transform **1** into **2-Zn** by only adding different mole amounts of potassium oxalate into the solution of **1** and monitor the reactions by ^{1}H NMR. The results are shown as following.

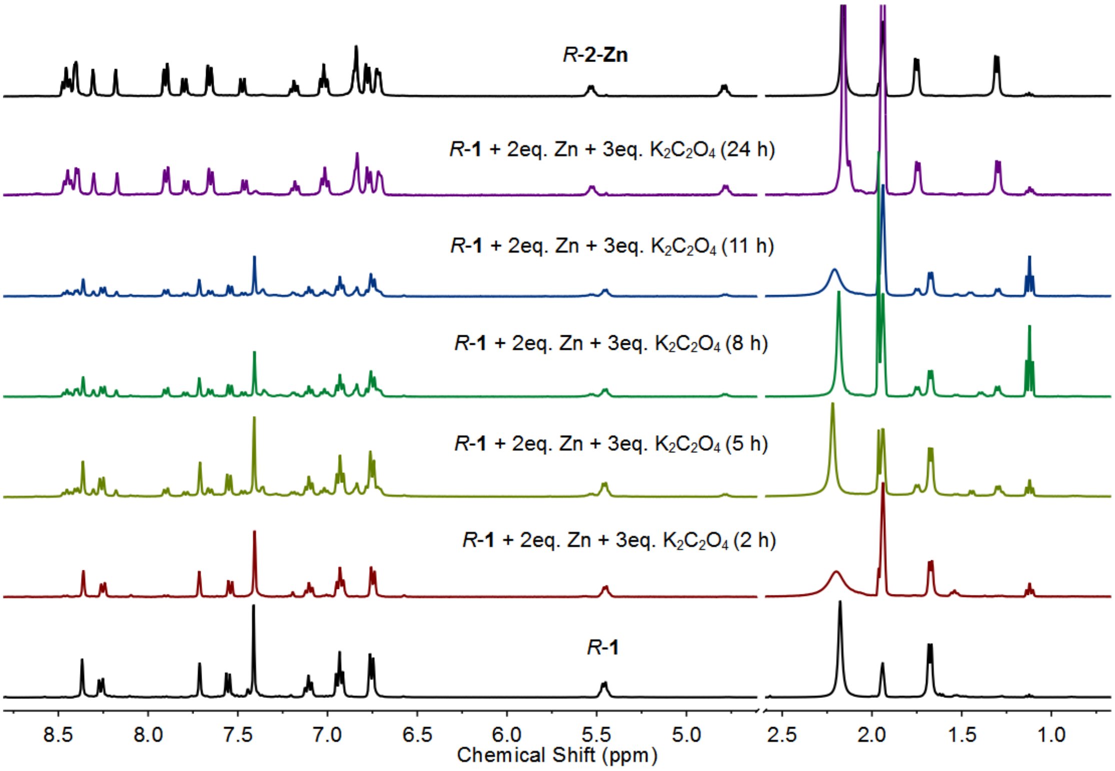

Supplementary Figure 18. ^{1}H NMR spectra (CD_3CN, 298K) of time-dependent reaction mixture containing *R*-**1** and 2 eq. $Zn(BF_4)_2$ and 3 eq. potassium oxalate with with *R*-**1**, *R*-**2**-Zn as references.

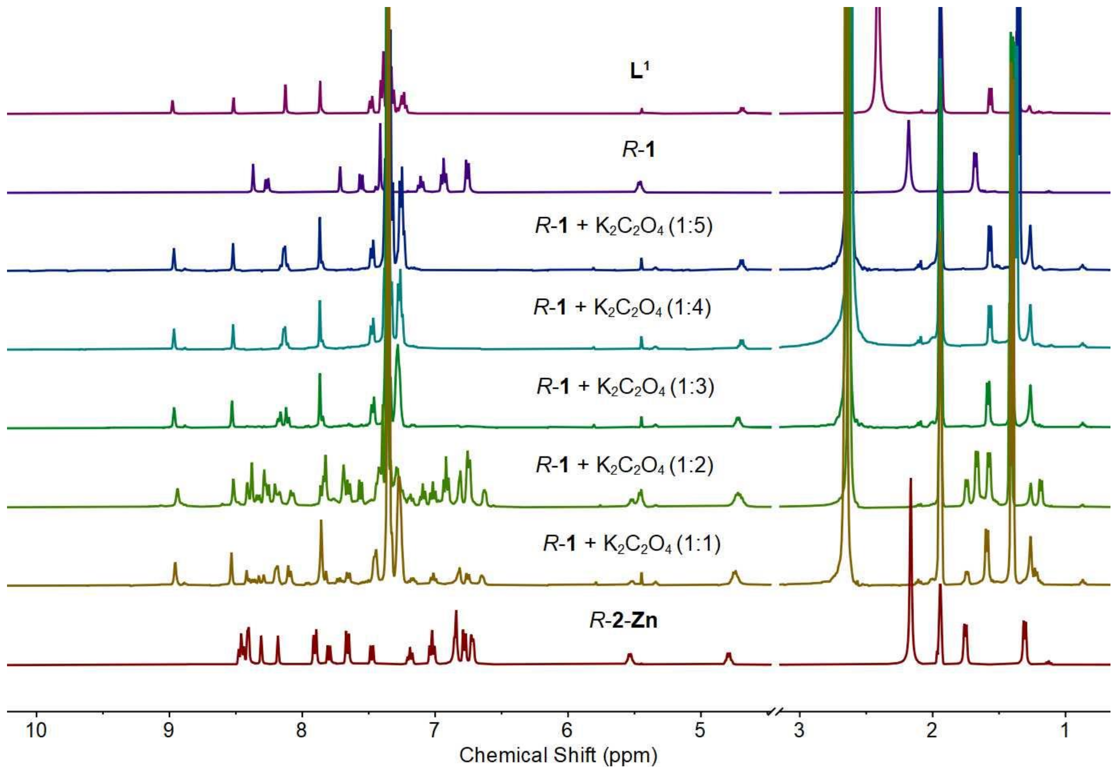

Supplementary Figure 19. ^{1}H NMR spectra (CD_3CN, 298K) of additions of different mole amounts of potassium oxalate into the solution of *R*-**1** with *R*-**1**, *R*-**2**-Zn and $\mathbf{L^1}$ as references. The addition of potassium oxalate without additional Zn^{2+} ions resulted in decomposition of tetrahedral cage **1**, indicating stronger coordination ability of oxalate ion than ligands with pyridylimines.

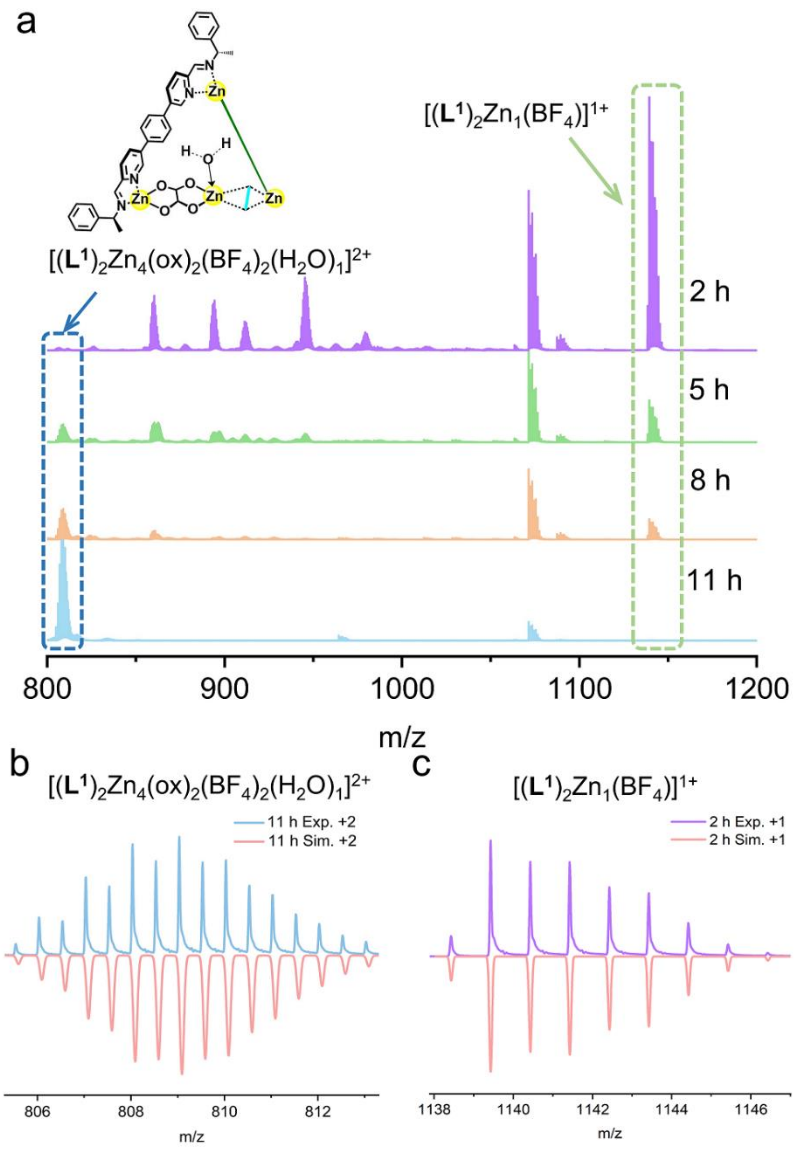

Supplementary Figure 20. a) ESI-MS spectra of reaction mixture containing *R*-**1** and 2 eq. $Zn(BF_4)_2$ and 3 eq. potassium oxalate at different reaction time with a key intermediate highlighted and a proposed structure showed, b) experimental and simulated isotopic patterns of intermediates $[(\mathbf{L^1})_2Zn_4(ox)_2(BF_4)_2(H_2O)_1]^{2+}$, c) experimental and simulated isotopic patterns of fragment $[(\mathbf{L^1})_2Zn_1(BF_4)]^{1+}$ from *R*-**1**. The $[(\mathbf{L^1})_2Zn_4(ox)_2(BF_4)_2(H_2O)_1]^{2+}$ is almost one-third of the final structure of *R*-**2**-**Zn**, and the intensity of this peak rises as time increases, while that of the fragment $[(\mathbf{L^1})_2Zn_1(BF_4)]^{1+}$ from *R*-**1** lowers down. $[(\mathbf{L^1})_2Zn_4(ox)_2(BF_4)_2(H_2O)_1]^{2+}$ is believed to be the intermediate when transforming *R*-**1** to *R*-**2**-**Zn**.

3.5 Attempts to induce cages by using 2, 2′-bipyrimdine or [Ru(2, 2′-bipyrimdine)$_3$]$^{2+}$

Using 2,2′-bipyrimidine instead of $K_2C_2O_4$

Subcomponent **A** (10 mg, 34.7 μmol) and *R*-1-phenylethylamine (9 μL, 69.4 μmol) were dissolved in 2 mL of acetonitrile. The mixture was stirred for 4 hours at 68 °C leading to the formation of ligand **L**^{1}. After that, zinc tetrafluoroborate hydrate (12 mg, 34.7 μmol) and 2,2′-bipyrimidine (3.0 mg, 17.4 μmol) were added into the mixture. The reaction mixture was stirred at 68 °C for another 48 hours. After that, the solvent was removed under vacuum. The crude product was dissolved in 0.5 mL of acetonitrile and precipitated by the addition of excess diethyl ether. The precipitate was further washed with diethyl ether. The product was dried in vacuum. The resulting solid was dissolved in CD_3CN for NMR analysis.

Using [Ru(2,2′-bipyrimdine)$_3$]·(BAr^F_4)$_2$ instead of $K_2C_2O_4$

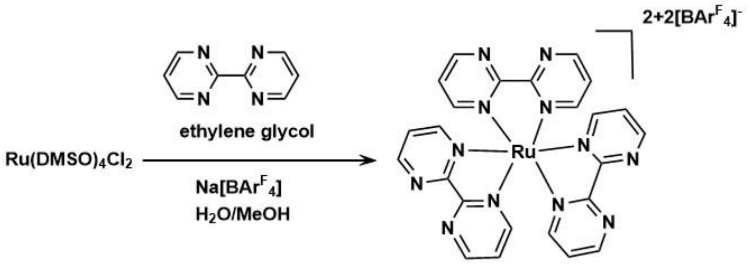

[Ru(2,2′-bipyrimdine)$_3$]·(BAr^F_4)$_2$ was prepared according to a literature.[1] $RuCl_2(DMSO)_4$ (336.9 mg, 0.695 mmol, 1.0 eq.) and 2,2'-bipyrimidine (550 mg, 3.47 mmol, 5.0 eq.) were added into a Schlenk flask, followed by ethylene glycol (20 mL). The solution was bubbled with nitrogen for 10 minutes and then heated to 120 °C for 72 hours under nitrogen. The solvent was removed in vacuo when the solution was cooled to 80 °C. The black crude material was dissolved in H_2O (10 mL) and adsorbed onto Al_2O_3. Purification was done by column chromatography (Al_2O_3; CH_3CN / H_2O; 100:0→90:10) to obtained a red solid (300 mg). A portion of the above red solid (100 mg) was further dissolved in H_2O (6 mL). Sodium tetrakis[3,5-bis(trifluoromethyl)phenyl]borate (Na[BAr^F_4], 280 mg, 0.317 mmol) which was dissolved in MeOH (3 mL) was further added dropwise into above solution with vigorous

stirring. The reaction was stirred at ambient temperature for 30 minutes and red precipitates were formed. The reaction was filtered to obtain the red solid, which was further washed with H_2O (2 × 1 mL) and dried in vacuo to give the title compound as an orange solid (200 mg, 87.2 μmol, 56.0%). ^1H NMR (400 MHz, acetone-d_6) δ 9.26 (dd, J = 4.9, 2.3 Hz, 6H, ArH), 8.77 (d, J = 5.6 Hz, 6H, ArH), 7.81-7.78 (m, 24H, Ar^FH + ArH), 7.67 (s, 8H, Ar^FH).

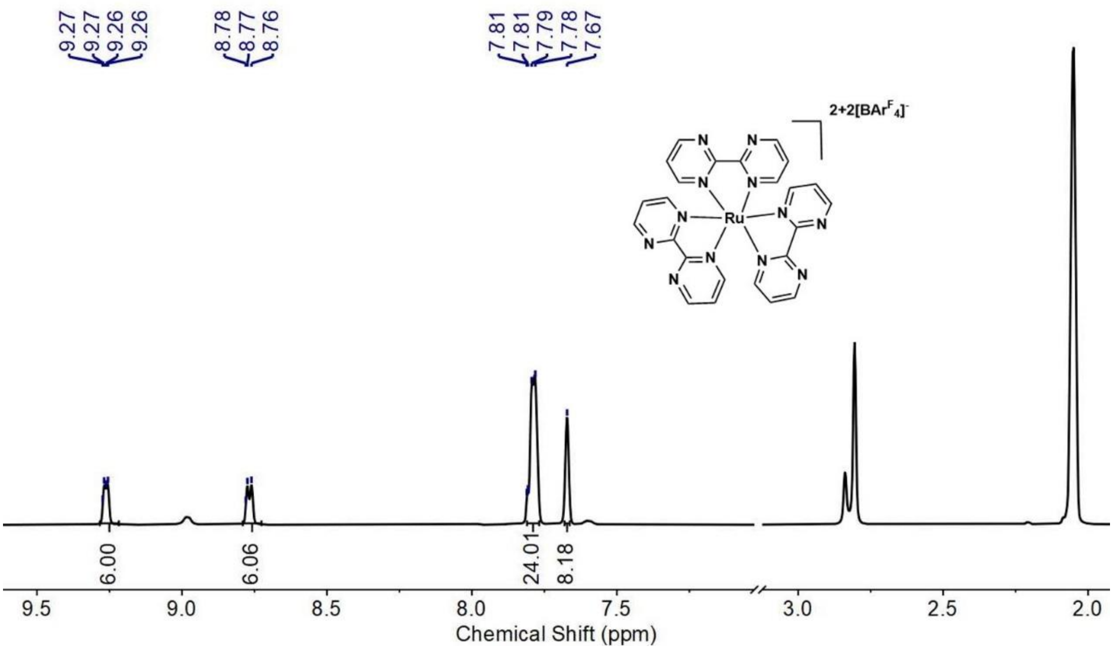

Supplementary Figure 21. ^1H NMR spectrum of [Ru(2,2'-bipyrimidine)$_3$][BAr^F_4]$_2$ in acetone-d_6 (400 MHz, 298 K).

Subcomponent **A** (10 mg, 34.7 μmol) and *R*-1-phenylethylamine (9 μL, 69.4 μmol) were dissolved in 2 mL of acetonitrile. The mixture was stirred for 4 hours at 68 °C leading to the formation of ligand $\mathbf{L^1}$. After that, zinc tetrafluoroborate hydrate (12 mg, 34.7 μmol) and [Ru(2,2′-bipyrimdine)$_3$]·(BAr^F_4)$_2$ (13.3mg, 5.8 μmol) were added, and the reaction mixture was stirred at 68 °C for another 48 hours. After that, the solvent was removed under vacuum. The crude product was dissolved in 0.5 mL of acetonitrile and precipitated by the addition of excess diethyl ether. The precipitate was further washed with diethyl ether. The product was dried in vacuum. The resulting solid was dissolved in CD_3CN for NMR analysis.

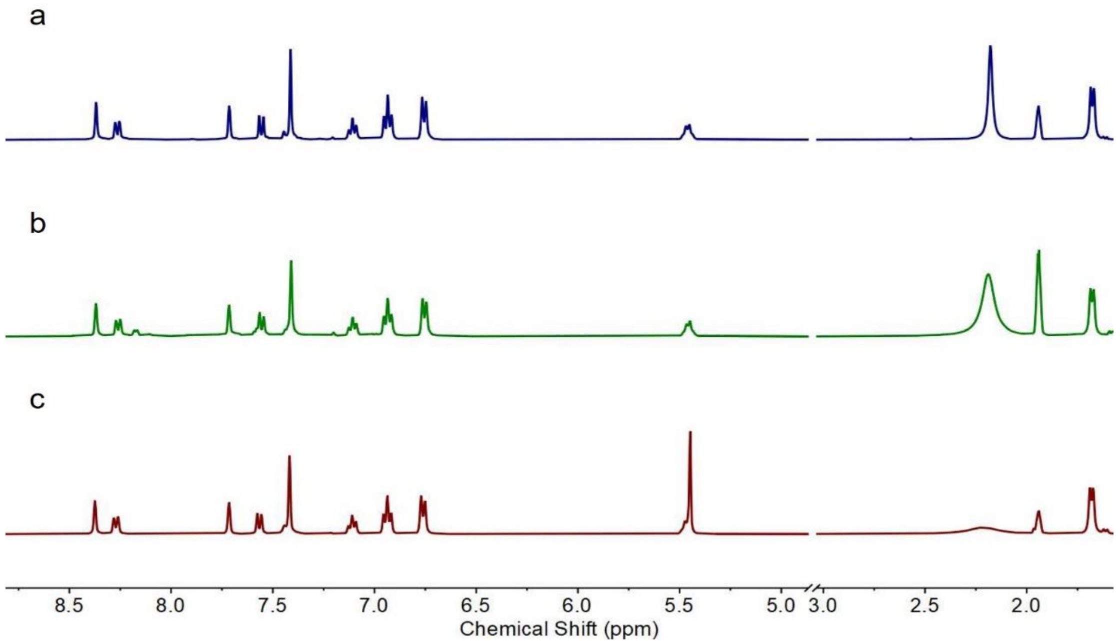

Supplementary Figure 22. ^{1}H NMR spectra of (a) **1** for reference; (b) products from above mentioned reaction which using 2,2′-bipyrimidine instead of $K_2C_2O_4$; and (c) products from above mentioned reaction which using [Ru(2,2′-bipyrimdine)$_3$]·(BAr^F$_4$)$_2$ instead of $K_2C_2O_4$ (400 MHz, CD_3CN, 298 K). According to the spectra, only **1** was formed in all reactions, indicating that 2,2′-bipyrimidine or pre-organized bpm, ie, [Ru(2,2′-bipyrimdine)$_3$]$^{2+}$, failed to induce a trigonal bipyramid.

3.6 Synthesis of cage 3

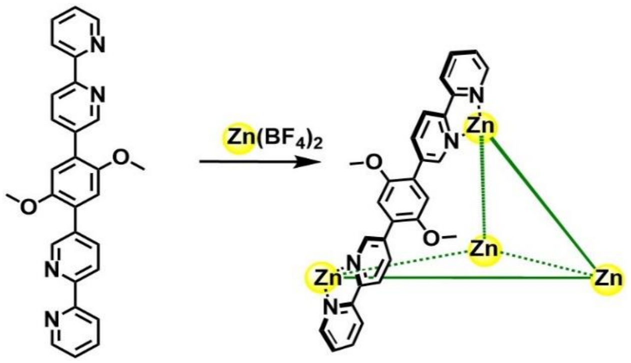

Subcomponent **L²** (10 mg, 22.4 μmol) and zinc tetrafluoroborate hydrate (7.8 mg, 22.4 μmol) were dissolved in 2 mL acetonitrile. The mixture was stirred at room temperature overnight. The resulting solution was filtered into a thin tube, and anhydrous diethyl ether was layered onto it. The diffusion led to crystals of cage **3**. About 10.0 mg of crystalline cage **3** was obtained, yield: 74%. The counterions (perchlorate, bis-(trifluoromethylsulfonyl)imide and tetrafluoroborate ions) of zinc salts did not affect the formation of the target cages. ^{1}H NMR (400 MHz, CD_3CN) δ 8.79 (s, 1H), 8.64 (d, *J* = 8.0 Hz, 2H), 8.43 (d, *J* = 8.4 Hz, 1H), 8.27 (dd, J_1= 8 Hz, J_2= 8 Hz, 1H), 7.93 (d, *J* = 5.2 Hz, 1H), 7.57 (dd, J_1= 6.4 Hz, J_2= 6.4 Hz, 1H), 7.0 (s, 1H), 3.32 (s, 3H). ^{13}C NMR (100 MHz, CD_3CN) δ 150.46, 148.94, 148.68, 147.80, 146.97, 141.64, 141.02, 135.94, 127.16, 124.98, 123.05, 122.81, 113.66, 55.67. ESI-MS (positive mode, CH_3CN, m/z, high resolution): calculated for $[(\mathbf{L}^2)_6Zn_4(BF_4)_6]^{2+}$: 1730.38, found: 1730.17; calculated for $[(\mathbf{L}^2)_6Zn_4(BF_4)_5]^{3+}$: 1124.87, found: 1124.59; calculated for $[(\mathbf{L}^2)_6Zn_4(BF_4)_4]^{4+}$: 821.95, found: 821.93.

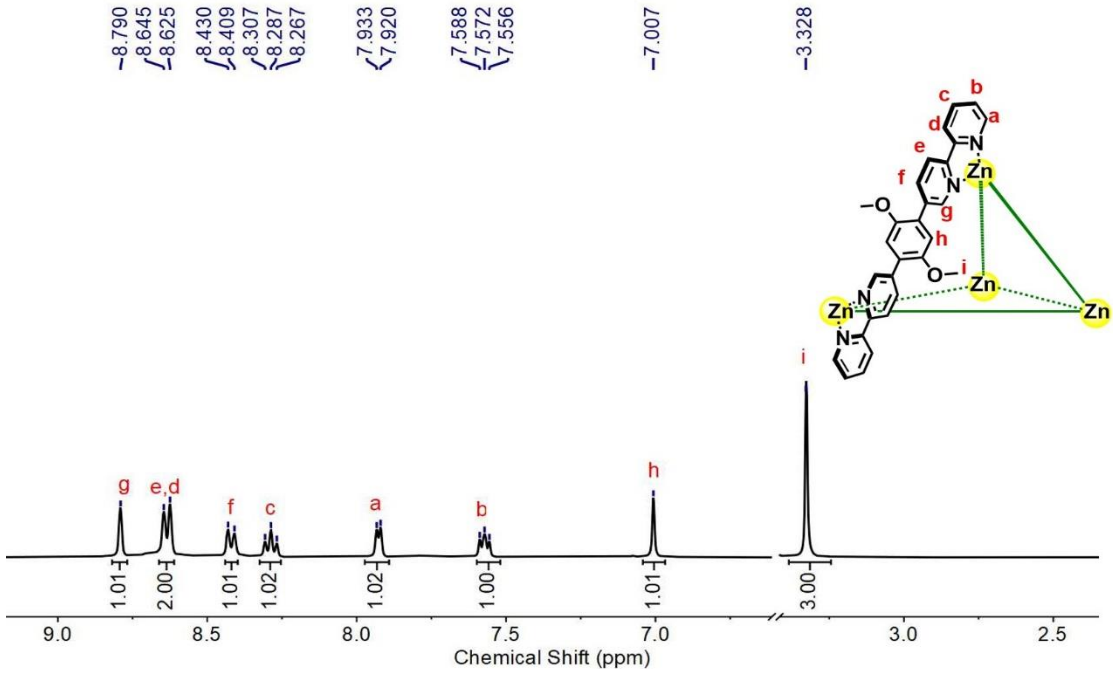

Supplementary Figure 23. ^{1}H NMR spectrum of **3** in CD_3CN (400 MHz, 298 K).

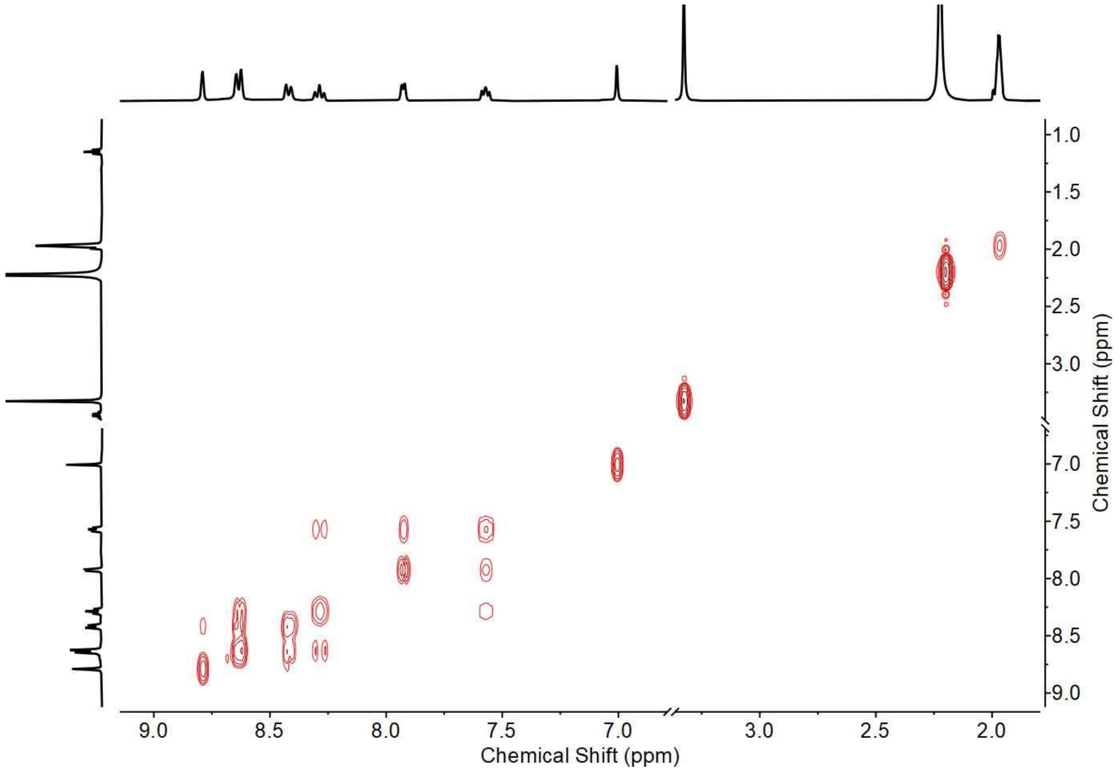

Supplementary Figure 24. ^{1}H-^{1}H COSY spectrum of **3** in CD_3CN (298 K).

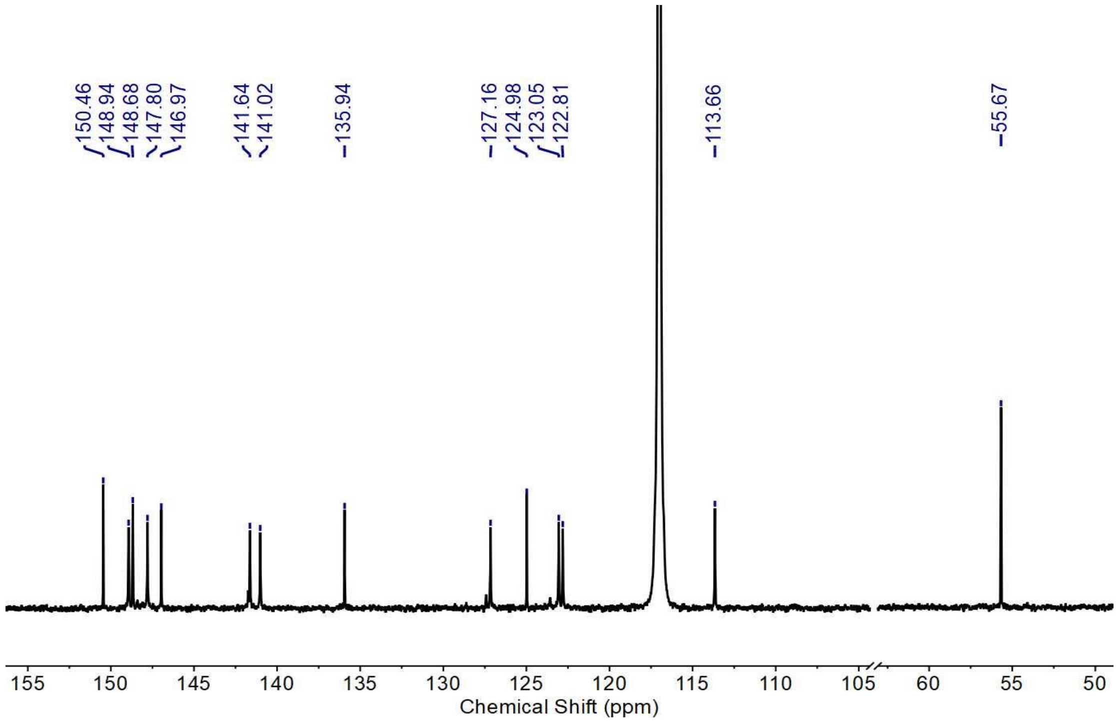

Supplementary Figure 25. ^{13}C NMR spectrum of **3** in CD_3CN (100 MHz, 298 K).

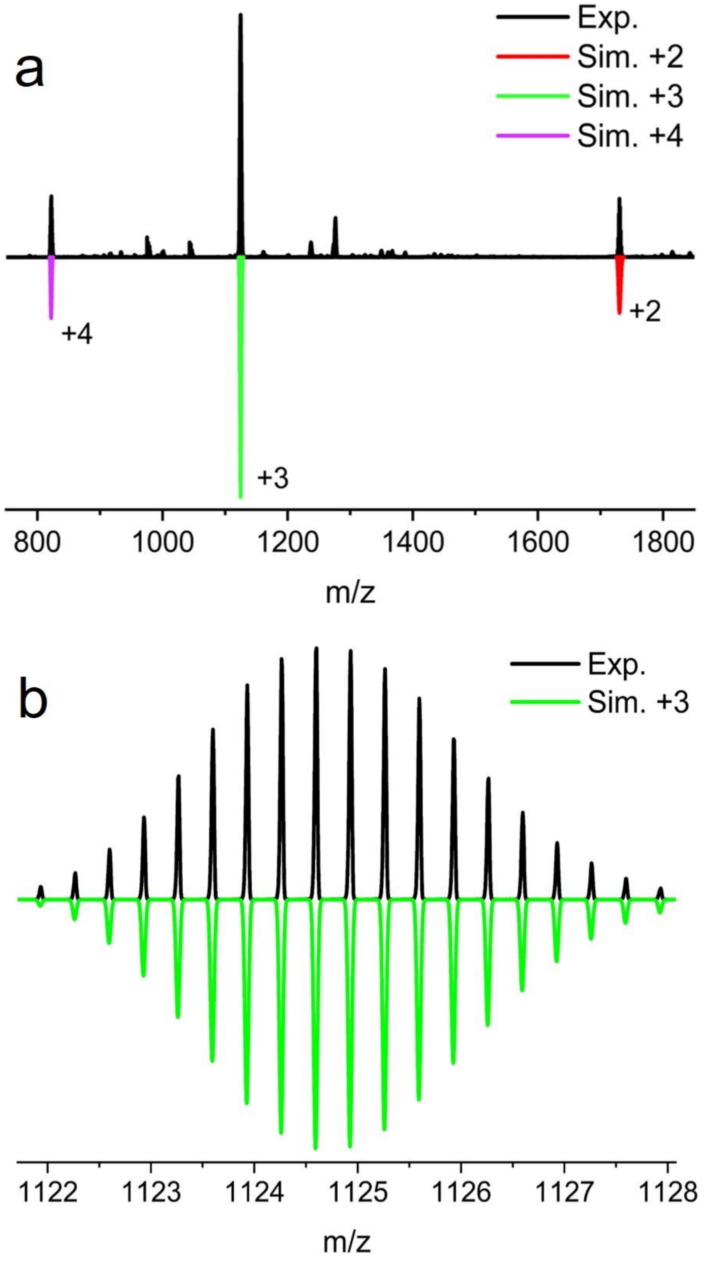

Supplementary Figure 26. a) ESI-MS spectrum of **3** in acetonitrile solution; b) experimental and simulated isotopic patterns of 3+ charged molecular peak of **3** by losing the corresponding numbers of counter-ions.

3.7 Synthesis of cage 4

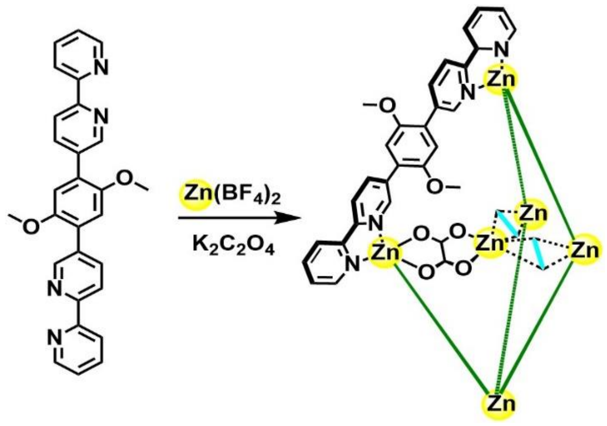

Subcomponent **L**^{2} (50mg, 112.0 μmol), zinc tetrafluoroborate hydrate (38.83 mg, 112.0 μmol) and $K_2C_2O_4$ (10.3 mg, 56.0 μmol) were dissolved in 2 mL acetonitrile. The mixture was stirred at 80 °C for 48 hours. After that, the solvent was removed under vacuum. The crude product was dissolved in 0.5 mL of acetonitrile and precipitated by the addition of excess diethyl ether. The precipitate was further washed with diethyl ether. The product was dried in vacuo. About 56.3 mg of cage **4** was obtained, yield: 78%. The counterions (perchlorate, bis-(trifluoromethylsulfonyl)imide and tetrafluoroborate ions) of zinc salts did not affect the formation of the target cages. ^{1}H NMR (400 MHz, CD_3CN) δ 8.63 (d, J = 8.8 Hz, 2H), 8.57 (s, 1H), 8.48 (d, J = 7.2 Hz, 3H), 8.40 (s, 1H), 8.31 (dd, J_1= 7.6 Hz, J_2= 8.0 Hz, 2H), 8.19 (dd, J_1= 7.6 Hz, J_2= 5.2 Hz 2H), 7.91 (d, J = 5.2 Hz, 1H), 7.63 (dd, J_1= 6.4 Hz, J_2= 6.8 Hz, 1H), 7.55 (dd, J_1= 6.4 Hz, J_2= 6.4 Hz, 1H), 6.91 (s, 1H), 6.82 (s, 1H), 3.50 (s, 3H), 3.41 (s, 3H). ^{13}C NMR (100 MHz, CD_3CN) δ 167.54, 151.08, 150.73, 149.23, 149.10, 148.99, 148.88, 148.42, 148.21, 148.07, 147.53, 145.10, 141.97, 141.75, 141.15, 139.09, 136.86, 136.46, 127.66, 126.81, 126.50, 125.72, 123.78, 122.77, 122.21, 117.39, 114.08, 65.35, 56.44, 56.21. ESI-MS (positive mode, CH_3CN, m/z, high resolution): calculated for $\{(\mathbf{L}^2)_6Zn_5[Zn(C_2O_4)_3](BF_4)_3\}^{3+}$: 1198.61, found: 1198.49; calculated for $\{(\mathbf{L}^2)_6Zn_5[Zn(C_2O_4)_3](BF_4)_2\}^{4+}$: 877.26, found: 877.13; calculated for $\{(\mathbf{L}^2)_6Zn_5[Zn(C_2O_4)_3](BF_4)_1\}^{5+}$: 684.44, found: 684.32; calculated for $\{(\mathbf{L}^2)_6Zn_5[Zn(C_2O_4)_3]\}^{6+}$: 555.90, found: 555.91.

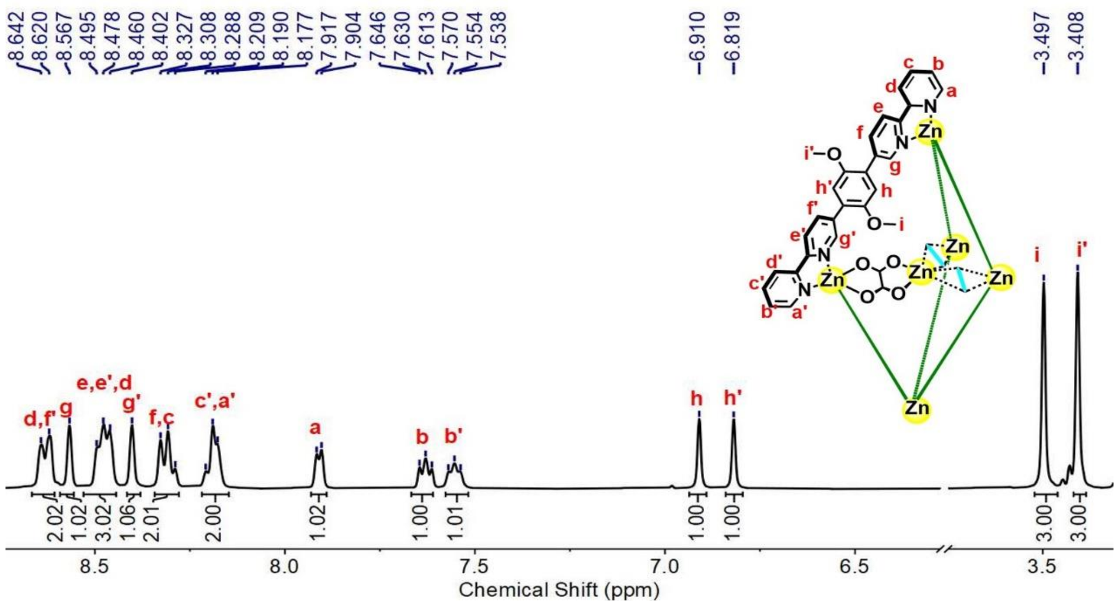

Supplementary Figure 27. ^{1}H NMR spectrum of **4** in CD_3CN (400 MHz, 298 K).

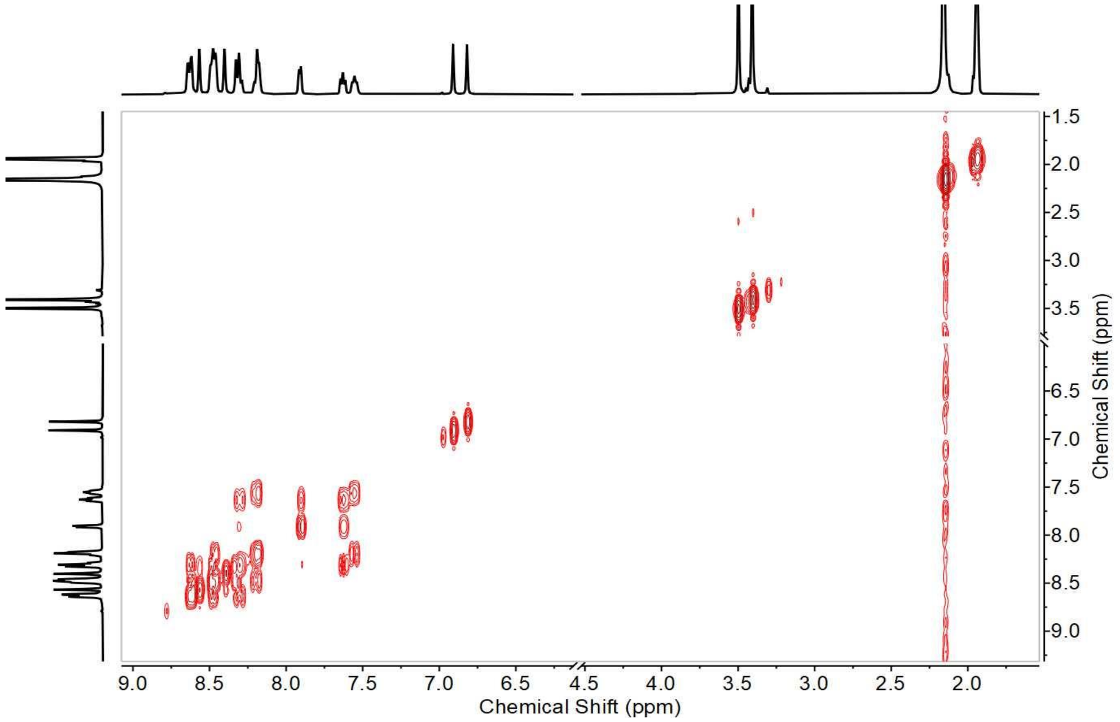

Supplementary Figure 28. ^{1}H-^{1}H COSY spectrum of **4** in CD_3CN (298 K).

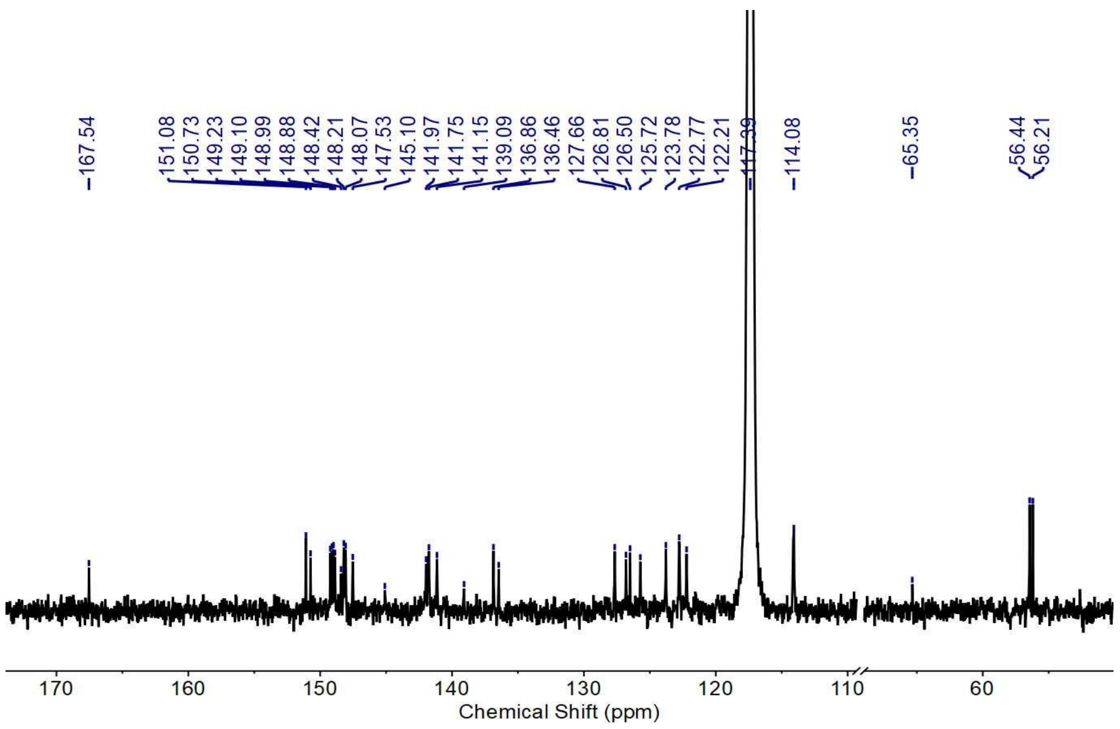

Supplementary Figure 29. ^{13}C NMR spectrum of **4** in CD_3CN (100 MHz, 298 K).

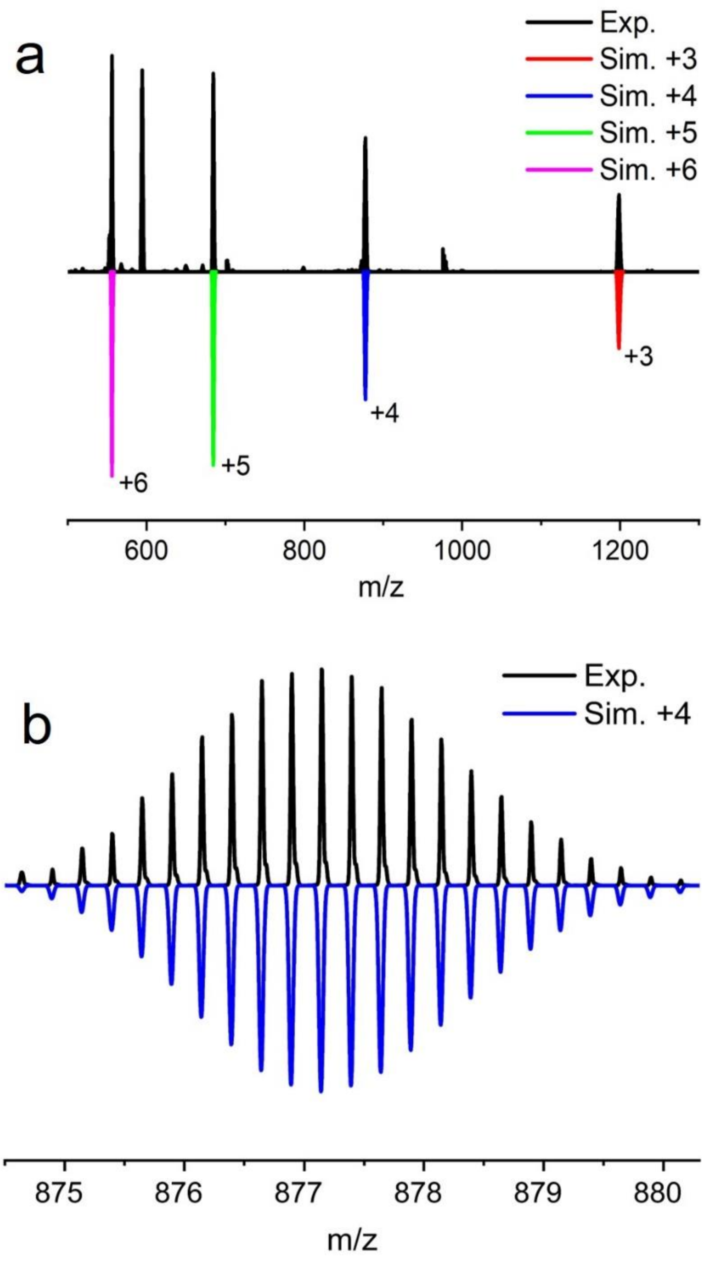

Supplementary Figure 30. a) ESI-MS spectrum of **4** in acetonitrile solution, b) experimental and simulated isotopic patterns of 4+ charged molecular peak of **4** by losing the corresponding numbers of counter-ions.

3.8 Synthesis of cage 5

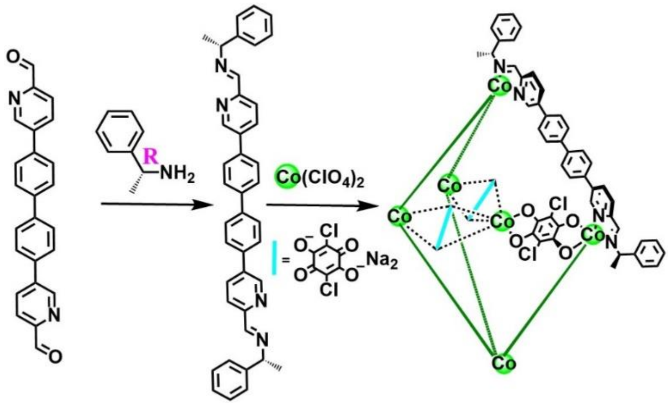

Subcomponent **B** (10 mg, 27.4 μmol) and *R*-1-phenylethylamine (7 μL, 55.2 μmol) were dissolved in 2 mL acetonitrile. The mixture was stirred at 68 °C for 4 hours to form $\mathbf{L^3}$. After that, cobalt (II) perchlorate hexahydrate (10.1 mg, 27.6 μmol) and chloranilic acid sodium salt hydrate (3.5 mg, 13.8 μmol) dissolving in 100 μL of methanol were added. The reaction solution was stirred at 80 °C for 48 hours. After cooling to room temperature, the resulting solution was filtered into a thin tube, and anhydrous diethyl ether was layered onto the filtrate. The slow diffusion led to crystals of *R*-**5**. About 10.4 mg of crystalline cage *R*-**5** was obtained, yield: 45%. *S*-**1** was obtained by replacing *R*-1-phenylethylamine with *S*-1-phenylethylamine. ESI-MS (positive mode, CH_3CN, m/z, high resolution): calculated for $\{(\mathbf{L^3})_6Co_5[Co(C_6O_4Cl_2)_3](ClO_4)_3\}^{3+}$: 1565.73, found: 1565.62; calculated for $\{(\mathbf{L^3})_6Co_5[Co(C_6O_4Cl_2)_3](ClO_4)_2\}^{4+}$: 1149.43, found: 1149.23; calculated for $\{(\mathbf{L^3})_6Co_5[Co(C_6O_4Cl_2)_3](ClO_4)_1\}^{5+}$: 899.66, found: 899.61; calculated for $\{(\mathbf{L^3})_6Co_5[Co(C_6O_4Cl_2)_3](ClO_4)_0\}^{6+}$: 733.14, found: 733.19.

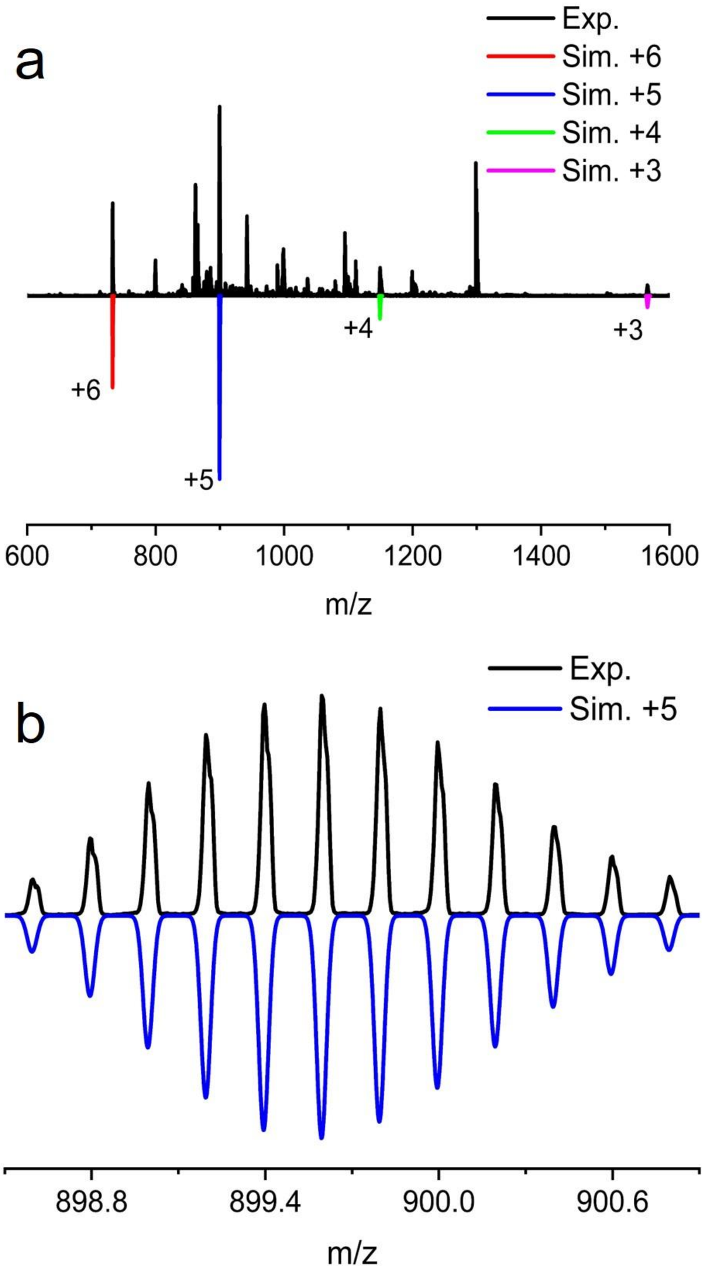

Supplementary Figure 31. a) ESI-MS spectrum of **5** in acetonitrile solution, b) experimental and simulated isotopic patterns of 5+ charged molecular peak of **5** by losing the corresponding numbers of counter-ions.

3.9 Synthesis of cage 6-Zn

Reactions involving subcomponent C without $K_2C_2O_4$

Subcomponent **C** (15 mg, 38.7 μmol) and *R*-1-phenylethylamine (10 μL, 77.6 μmol) were dissolved in 2 mL acetonitrile. The mixture was stirred at 68 °C for 4 hours to form $\mathbf{L^4}$, after cooling to room temperature, zinc tetrafluoroborate hydrate (when $\mathbf{L^4}$: Zn = 6 : 6: 13.4 mg, 38.7 μmol; when $\mathbf{L^4}$: Zn = 6 : 4: 8.9 mg, 25.8 μmol) were added. The reaction mixture was stirred at room temperature overnight. After that, the solvent was removed under vacuum. The crude product was dissolved in 0.5 mL acetonitrile and precipitated by the addition of excess diethyl ether. The precipitate was further washed with diethyl ether. The product was dried in vacuo. About 10 mg of precipitate was obtained, which was further examined by ^{1}H NMR.

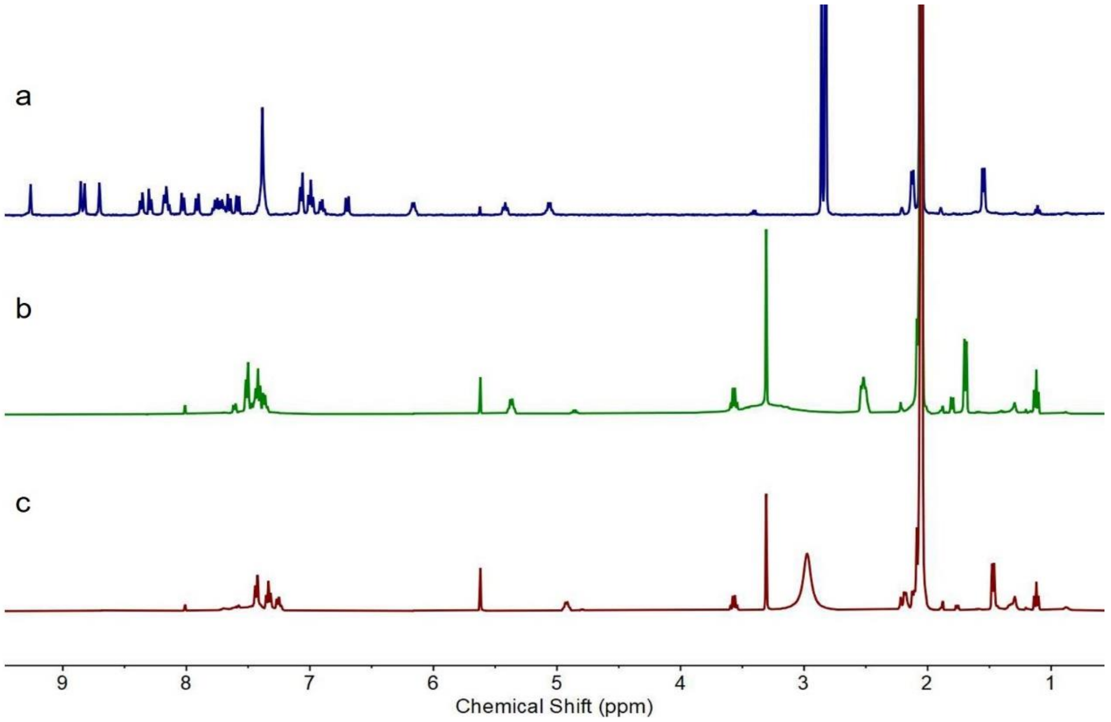

Supplementary Figure 32. ^{1}H NMR spectra of a) **6-Zn** for references; products from above mentioned reaction which is the same processes without the addition of $K_2C_2O_4$ b) when $\mathbf{L^4}$ **:** Zn = 6 : 6; and c) $\mathbf{L^4}$: Zn =6 : 4 (400 MHz, acetone-d$_6$, 298 K). According to the spectra, without the addition of $K_2C_2O_4$, no well-defined cage products could be formed in the reactions.

Reactions involving subcomponent C with $K_2C_2O_4$

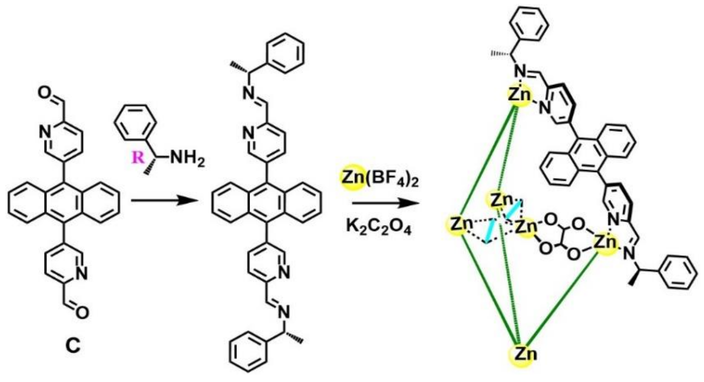

Subcomponent **C** (15 mg, 38.7 μmol) and *R*-1-phenylethylamine (10 μL, 77.6 μmol) were dissolved in 2 mL acetonitrile. The mixture was stirred at 68 °C for 4 hours to form $\mathbf{L^4}$. After cooling to room temperature, zinc tetrafluoroborate hydrate (13.4 mg, 38.7 μmol) and $K_2C_2O_4$ (3.5 mg, 19.0 μmol) dissolving in 30 μL of water were added. The resulting mixture was stirred at room temperature overnight. After that, the solution was filtered into a thin tube, and anhydrous diethyl ether was layered onto it. The slow diffusion led to crystals of *R*-**6-Zn**. About 23.2 mg of crystalline cage *R*-**6-Zn** was obtained, yield: 74%. *S*-**6-Zn** was obtained by replacing *R*-1-phenylethylamine with *S*-1-phenylethylamine. The counterions (perchlorate, bis-(trifluoromethylsulfonyl)imide and tetrafluoroborate ions) of zinc salts did not affect the formation of the target cages. ^{1}H NMR (400 MHz, acetone-d_6) δ 9.26 (s, 1H), 8.85 (s, 1H), 8.82 (s, 1H), 8.70 (s, 1H), 8.36 (d, J = 7.6 Hz, 1H), 8.29 (d, J = 8.0 Hz, 1H), 8.17 (dd, J_1= 8.0 Hz, J_2= 10.0 Hz, 2H), 8.03 (d, J = 7.6 Hz, 1H), 7.91 (d, J = 8.8 Hz, 1H), 7.77 (dd, J_1= 7.6 Hz, J_2= 8.0 Hz, 1H) 7.72 (dd, J_1= 7.6 Hz, J_2= 8.0 Hz, 1H), 7.64 (d, J = 8.8 Hz, 1H), 7.57(d, J= 9.2 Hz, 1H), 7.38 (m, 5H), 7.07 (d, J = 7.6 Hz, 2H), 6.99 (dd, J_1= 7.6 Hz, J_2= 10.0 Hz, 2H), 6.90 (dd, J_1= 7.2 Hz, J_2= 7.2 Hz, 1H), 6.70 (d, J = 8.4 Hz, 1H), 6.17 (q, J_1= 6.4 Hz, J_2= 6.4 Hz, 1H), 5.43 (dd, J_1= 8.0 Hz, J_2= 7.6 Hz, 1H), 5.06 (q, J_1= 6.8 Hz, J_2= 6.8 Hz, 1H), 2.12 (d, J = 6.4 Hz, 3H), 1.55 (d, J = 6.8 Hz, 3H). ^{13}C NMR (100 MHz, CD_3CN) δ 169.03, 163.48, 162.03, 150.97, 150.72, 148.01, 146.69, 145.65, 145.49, 141.97, 140.49, 139.96, 132.69, 132.67, 130.93, 130.50, 130.36, 130.26, 130.17, 129.92, 129.73, 129.59, 129.21, 128.93, 128.36, 127.73, 127.57, 127.06, 127.01, 126.84, 126.22, 125.77, 125.30, 66.19, 65.64, 65.36, 55.24, 23.71, 21.05, 15.53. ESI-MS (positive mode, CH_3CN, m/z, high resolution): calculated for

$\{(\mathbf{L^4})_6Zn_5[Zn(C_2O_4)_3](C_2NS_2O_4F_6)_3\}^{3+}$: 1688.45, found: 1688.69; calculated for $\{(\mathbf{L^4})_6Zn_5[Zn(C_2O_4)_3](C_2NS_2O_4F_6)_2\}^{4+}$: 1196.30, found: 1196.23; calculated for $\{(\mathbf{L^4})_6Zn_5[Zn(C_2O_4)_3](C_2NS_2O_4F_6)_1\}^{5+}$: 901.01, found: 901.02; calculated for $\{(\mathbf{L^4})_6Zn_5[Zn(C_2O_4)_3](C_2NS_2O_4F_6)_0\}^{6+}$: 704.15, found: 704.02.

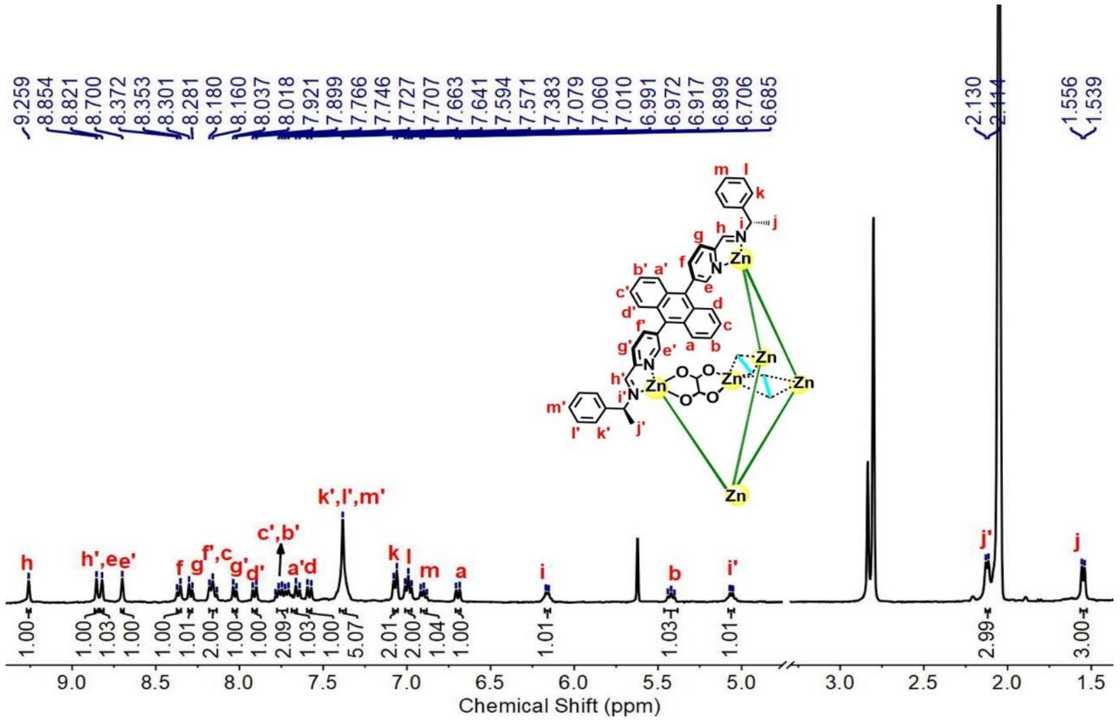

Supplementary Figure 33. ^{1}H NMR spectrum of **6-Zn** in acetone-d_6 (400 MHz, 298 K).

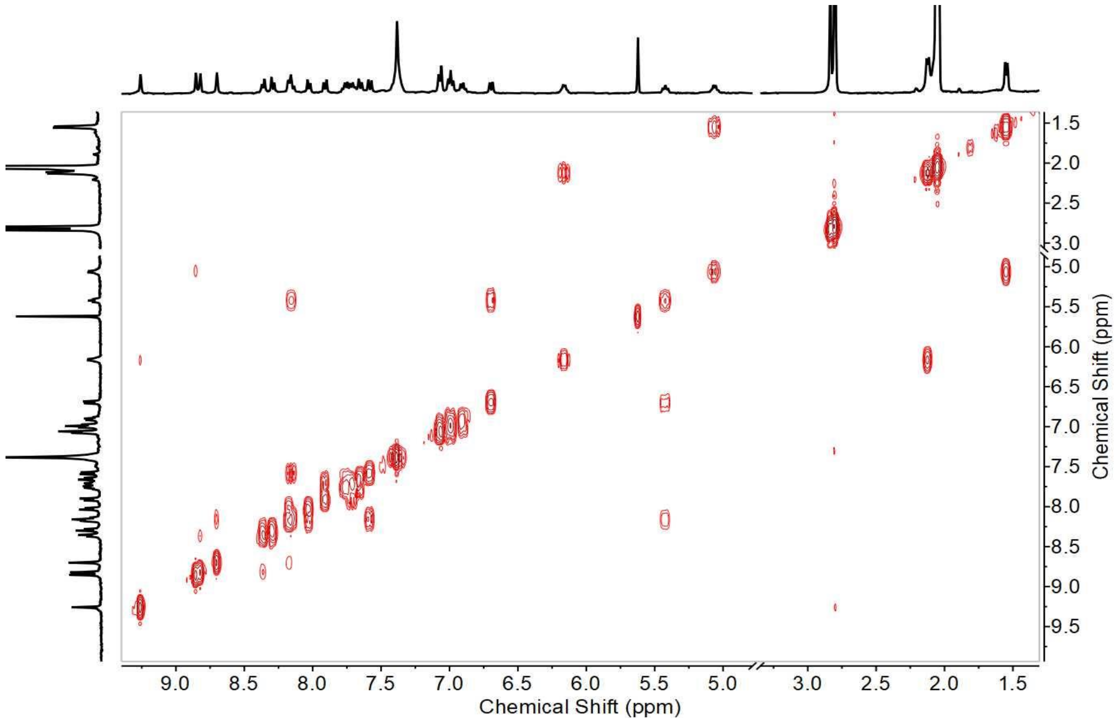

Supplementary Figure 34. ^{1}H-^{1}H COSY spectrum of **6-Zn** in acetone-d_6 (298 K).

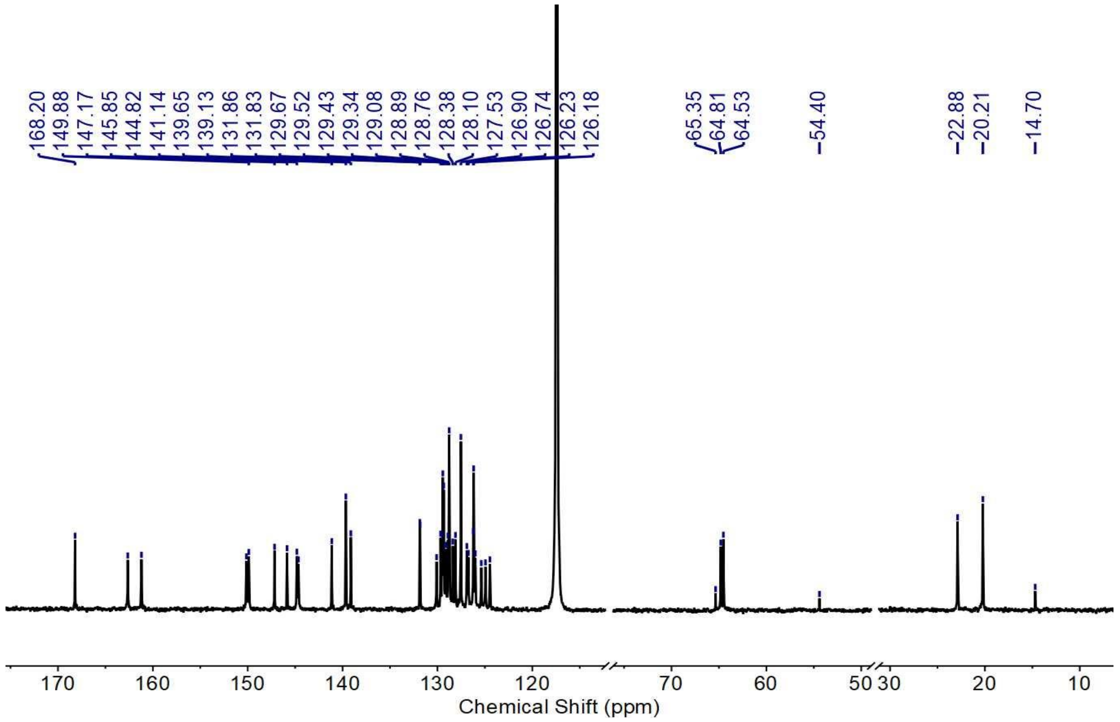

Supplementary Figure 35. ^{13}C NMR spectrum of **6-Zn** in CD_3CN (100 MHz, 298 K).

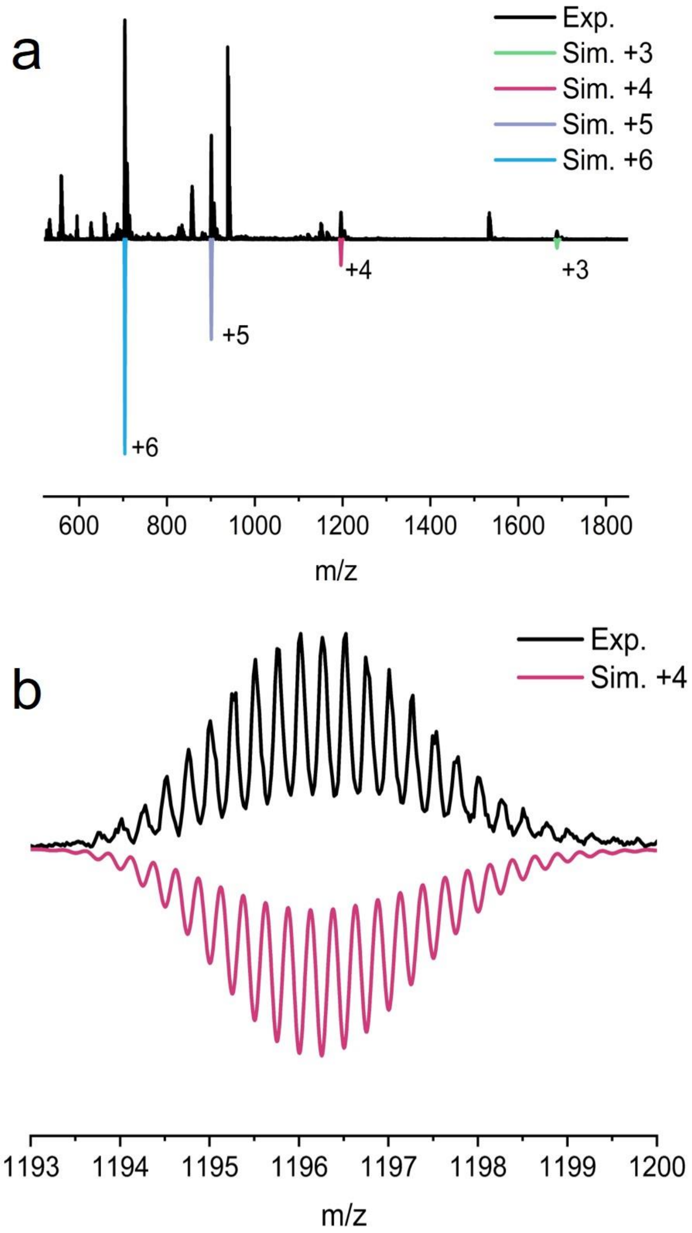

Supplementary Figure 36. a) ESI-MS spectrum of **6-Zn**, b) experimental and simulated isotopic patterns of 4+ charged molecular peak of **6-Zn** by losing the corresponding numbers of counter-ions.

3.10 Synthesis of cage 6-Co

Subcomponent **C** (15 mg, 38.7 μmol) and *S*-1-phenylethylamine (10 μL, 77.6 μmol) were dissolved in 2 mL acetonitrile. The mixture was stirred at 68 °C for 4 hours to form $\mathbf{L^4}$. After cooling to room temperature, cobalt (II) perchlorate hexahydrate (14.0 mg, 38.7 μmol) and $K_2C_2O_4$ (3.5 mg, 19.0 μmol) dissolving in 30 μL of water were added. The resulting mixture was stirred at room temperature overnight. After that, the solution was filtered into a thin tube, and anhydrous diethyl ether was layered onto it. The slow diffusion led to red crystals of *S*-**6-Co**. About 26.5 mg of crystalline cage *S*-**6-Co** was obtained, yield: 86%. *R*-**6-Zn** was obtained by replacing *R*-1-phenylethylamine with *S*-1-phenylethylamine. ESI-MS (positive mode, CH_3CN, m/z, high resolution): calculated for $\{(\mathbf{L^4})_6Co_5[Co(C_2O_4)_3](ClO_4)_1\}^{5+}$: 857.07, found: 857.04; calculated for $\{(\mathbf{L^4})_6Co_5[Co(C_2O_4)_3](ClO_4)_2\}^{4+}$: 1096.02, found: 1096.05; calculated for $\{(\mathbf{L^4})_6Co_5[Co(C_2O_4)_3](ClO_4)_3\}^{3+}$: 1494.68, found: 1494.70.

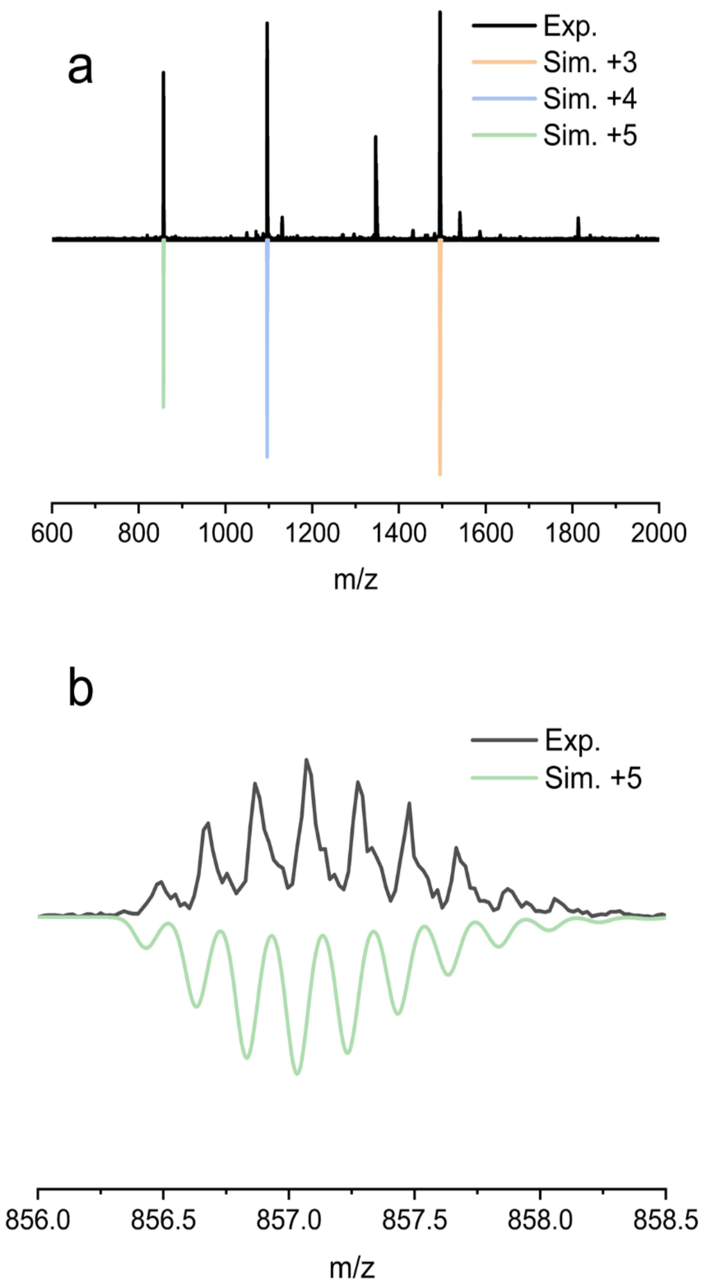

Supplementary Figure 37. a) ESI-MS spectrum of **6-Co**, b) experimental and simulated isotopic patterns of 5+ charged molecular peak of **6-Co** by losing the corresponding numbers of counter-ions.

3.11 Synthesis of cage 6-Zn-O_2

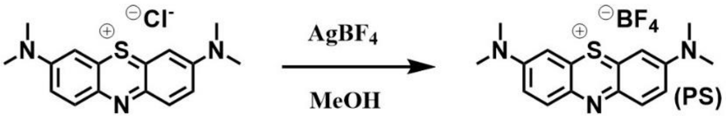

Methylene blue hydrate (50 mg, 0.157 mmo1) and $AgBF_4$ (340 mM, 0.98 mL, 66.4 μmol, 2.5 eq) were mixed in 25 mL of MeOH. After stirring for 1 hour, the solvent was evaporated under vacuum to obtain a dark blue solid, which was further suspended in Et_2O (15.0 mL) and filtrated. The residual solid was washed with Et_2O (1 mL × 2) and extracted with CH_2Cl_2 (ca. 10 mL).[2] About 170 mg of products (**PS**) was obtained, yield: 40%. ^{1}H NMR (400 MHz, acetone-d$_6$) δ 8.00 (dd, J = 9.6, 1.0 Hz, 2H), 7.60 (dd, J = 9.6, 2.7 Hz, 2H), 7.51 (dd, J = 2.9, 1.0 Hz, 2H), 3.52 (d, J = 1.0 Hz, 12H).

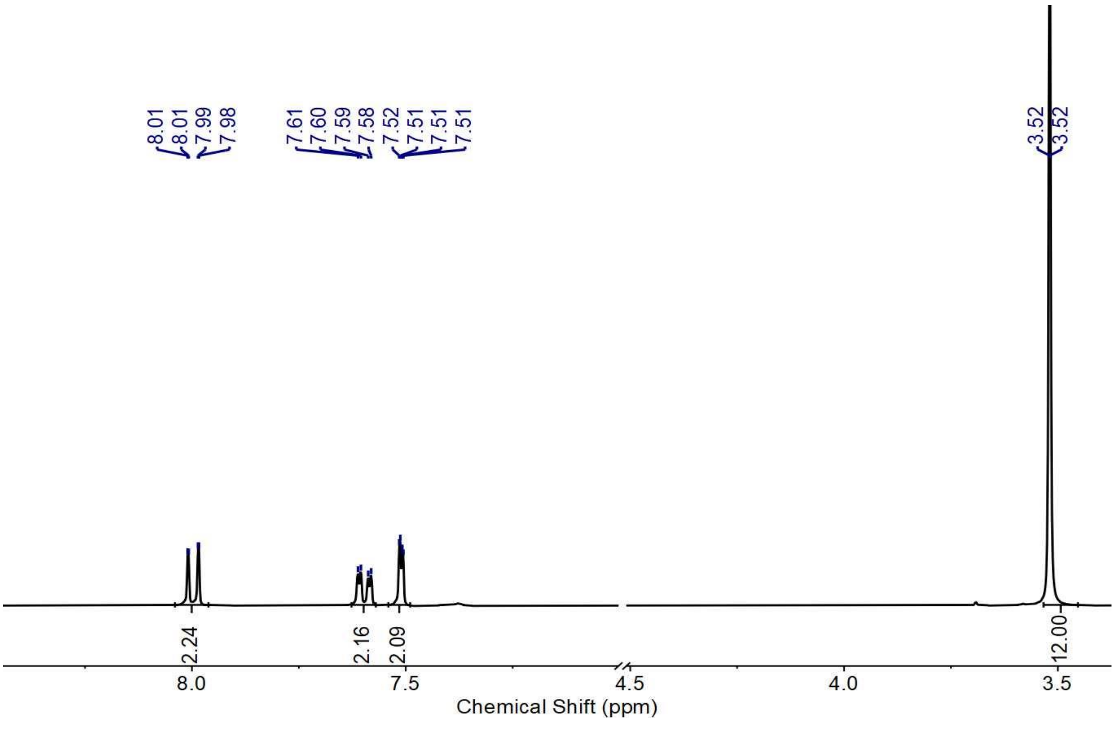

Supplementary Figure 38. ^{1}H NMR spectrum of **PS** in acetone-d$_6$ (400 MHz, 298 K).

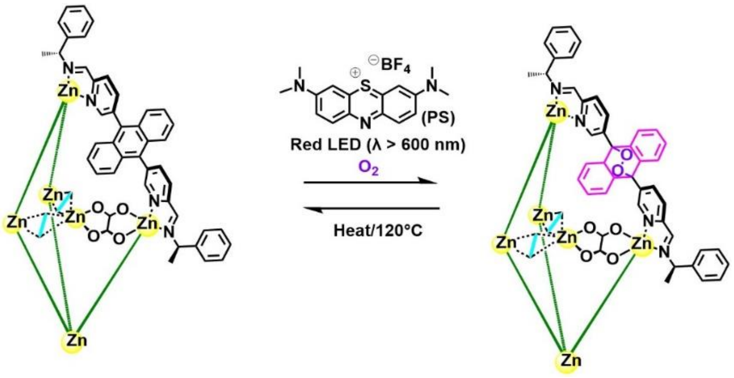

PS (0.5 mg, 0.118 µmol, 0.1 eq.) was added into an acetone (600 µL) solution containing *R*-**6** (5.6 mg, 1.18 µmol) in a glass vessel equipped with a stir bar and capped with a rubber plug. The mixture was then filled with oxygen to keep it in an oxygen atmosphere while being irradiated with red lights using a LED lamp (SMD 5050 RGB, dominant wavelength range: 620-635 nm, λ_{max} = 630 nm) at room temperature for 16 hours. After that, the solvent was removed under vacuum. The crude product was dissolved in 0.5 mL acetone and excessive diethyl ether was added to precipitate. The precipitate was collected by centrifugation. The same processes were repeated by using dichloromethane, n-hexane, and methanol instead of acetone, respectively, aiming at removing the photocatalyst. The final produce was dry under vacuum. About 4.3 mg of cage *R*-**6-Zn-O$_2$** was obtained, yield: 74%. *S*-**6-Zn-O$_2$** was obtained by replacing cage *R*-**6-Zn** with cage *S*-**6-Zn**. The counterions (perchlorate, bis-(trifluoromethylsulfonyl)imide and tetrafluoroborate ions) of zinc salts did not affect the formation of the target cages. ^{1}H NMR (400 MHz, acetone-d$_6$) δ 9.32 (s, 1H), 9.25 (s, 1H), 8.76 (s, 1H), 8.57 (d, *J* = 8.4 Hz, 1H), 8.53 (d, *J* = 8.4 Hz, 1H), 8.35 (d, *J* = 8.4 Hz, 1H), 8.34 (s, 1H), 8.17 (d, *J* = 8 Hz, 1H), 8.03 (dd, J_1= 8.4 Hz, J_2= 7.6 Hz,1H), 7.37 (d, *J* = 7.6 Hz, 1H), 7.27-7.21 (m, 7H), 7.14 (dd, J_1= 7.6 Hz, J_2= 7.6 Hz, 3H), 7.05 (dd, J_1= 7.6 Hz, J_2= 7.6 Hz,, 3H), 6.87 (d, *J* = 8 Hz, 1H), 6.07 (q, J_1= 4.8 Hz, J_2= 4.8 Hz 1H), 5.13 (m, 2H), 4.23 (dd, J_1= 8 Hz, J_2= 7.6 Hz, 1H), 2.08 (d, *J* = 4.8 Hz, 3H), 1.71 (d, *J* = 7.2 Hz, 3H). The ^{13}C NMR spectrum of cage **6-Zn-O$_2$** was unable to obtain due to the low solubility of the sample in acetone-d$_6$ or CD_3CN. ESI-MS (positive mode, CH_3CN, m/z, high resolution): calculated for $\{(\mathbf{L^4})_6Zn_5[Zn(C_2O_4)_3](O_2)_6(BF_4)_3\}^{3+}$: 1559.11, found: 1559.03; calculated for $\{(\mathbf{L^4})_6Zn_5[Zn(C_2O_4)_3](O_2)_6(BF_4)_2\}^{4+}$: 1147.63, found: 1147.53; calculated for

$\{(\mathbf{L^4})_6Zn_5[Zn(C_2O_4)_3](O_2)_6(BF_4)_1\}^{5+}$: 900.62, found: 900.63; calculated for $\{(\mathbf{L^4})_6Zn_5[Zn(C_2O_4)_3](O_2)_6(BF_4)_0\}^{6+}$: 736.15, found: 736.03.

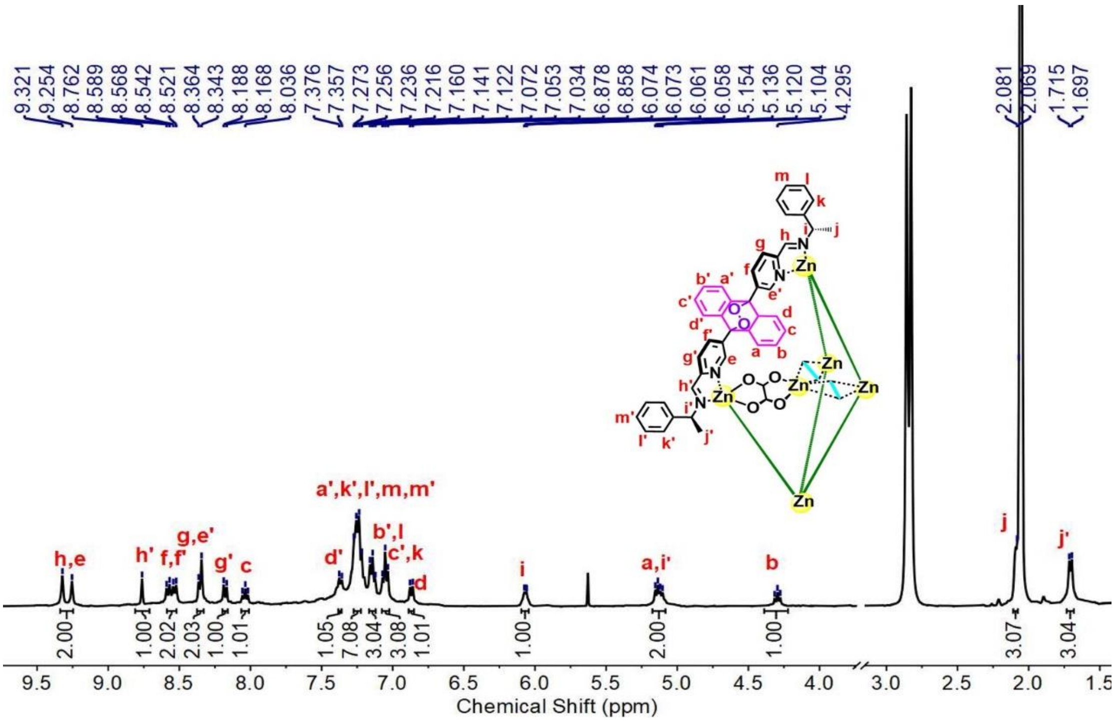

Supplementary Figure 39. ^{1}H NMR spectrum of **6-Zn-O$_2$** in acetone-d$_6$ (400 MHz, 298 K).

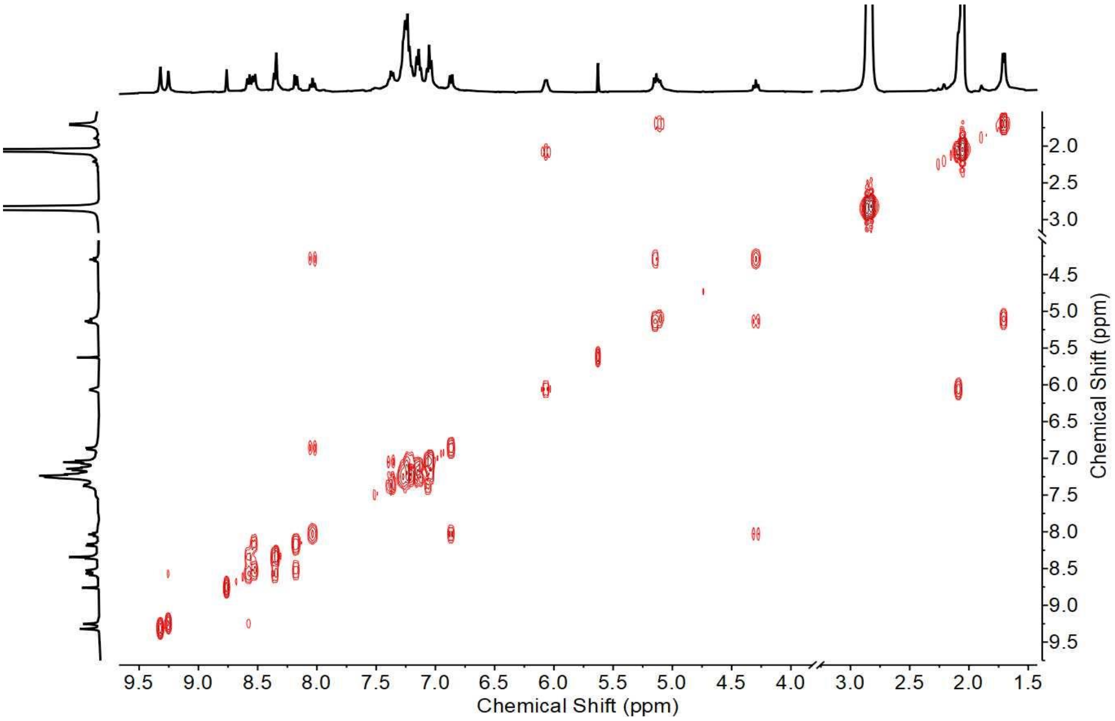

Supplementary Figure 40. ^{1}H-^{1}H COSY spectrum of **6-Zn-O$_2$** in acetone-d$_6$ (298 K).

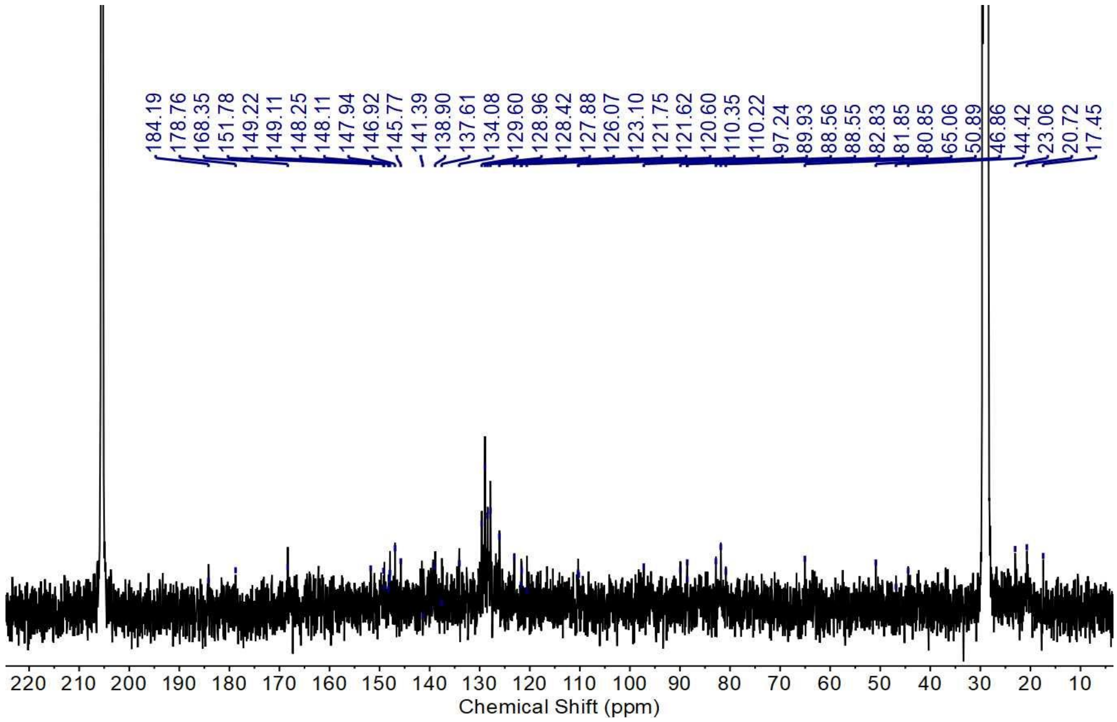

Supplementary Figure 41. ^{13}C NMR spectrum of **6-Zn-O$_2$** in acetone-d$_6$ (100 MHz, 298 K).

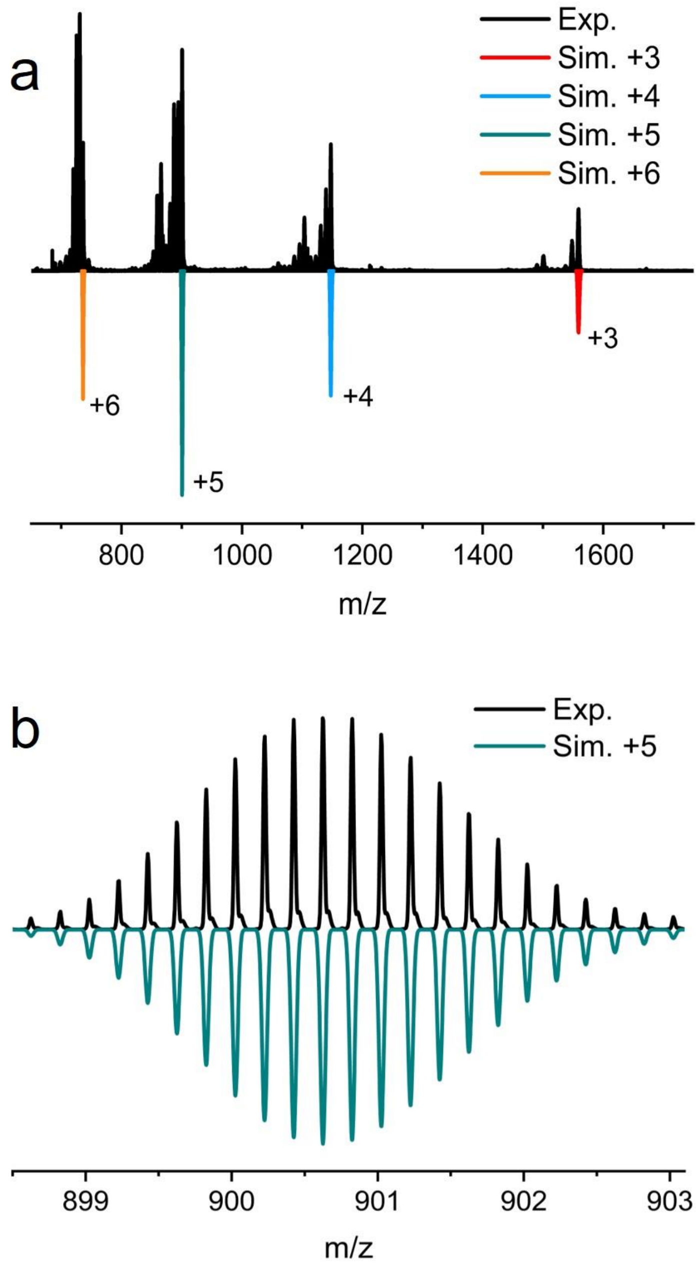

Supplementary Figure 42. a) ESI-MS spectrum of **6-Zn-O_2** in acetonitrile solution, b) experimental and simulated isotopic patterns of 5+ charged molecular peak of **6-Zn-O_2** by losing the corresponding numbers of counter-ions. Fragments of partial loss of the six O_2 on the ligands were also observed, which may be produced under the ESI condition.

4. Density functional theory (DFT) calculations

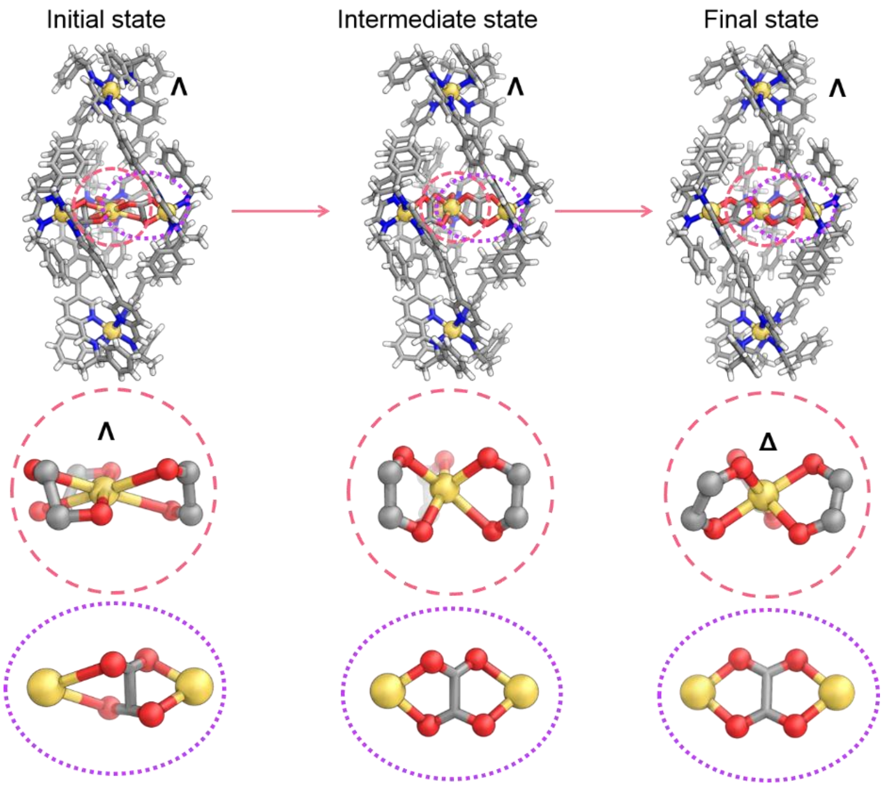

Supplementary Figure 43. The structural optimization of a mismatched pair of Λ-Zn(ox)$_3$⊂Λ$_5$-Zn$_5$(*R*-**L^1**)$_6$ using Gaussian 16 with B3LYP function and 6-31G(d) basis set. The initial state is a Λ-Zn(ox)$_3$⊂Λ$_5$-Zn$_5$(*R*-**L^1**)$_6$, while energy minimum state after structural optimization is a Δ-Zn(ox)$_3$⊂Λ$_5$-Zn$_5$(R-**L^1**)$_6$.

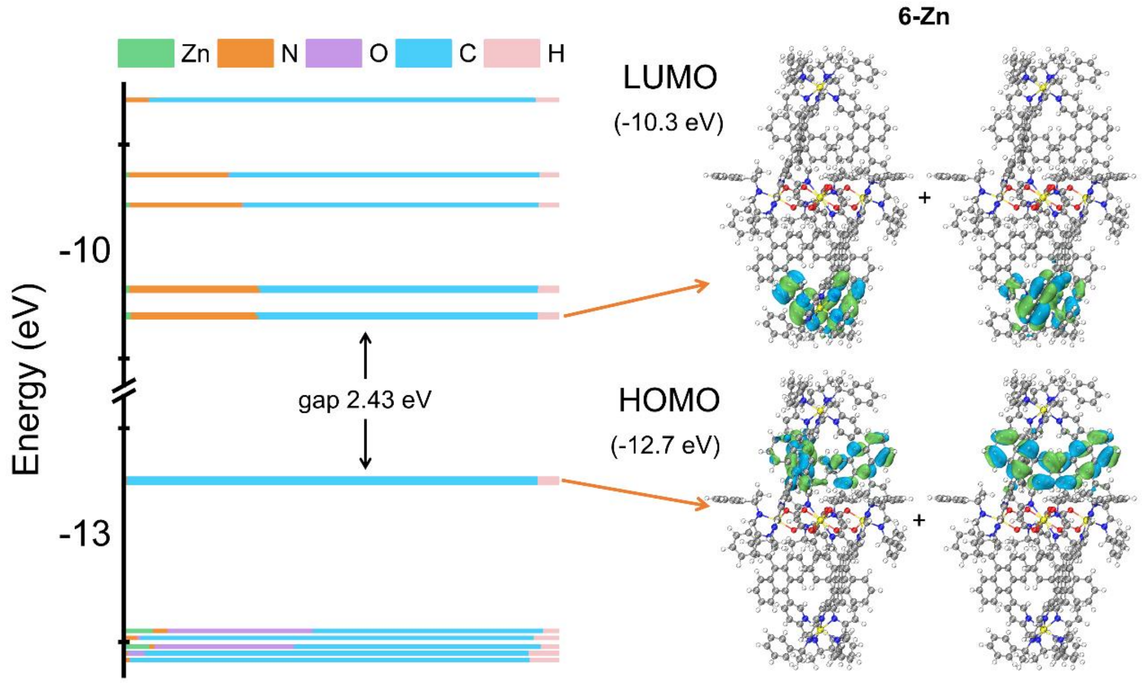

Supplementary Figure 44. Kohn−Sham frontier orbital diagram of **6-Zn** with localizations of all elements, and the HOMO and LUMO distributions (isovalue = 0.02) of **6-Zn** based on the TD-DFT calculations with B3LYP function and 6-31G(d) basis set. Color code of the atoms: zinc, yellow; carbon, gray; oxygen, red; nitrogen, blue; hydrogen, white.

5. Release of singlet oxygen

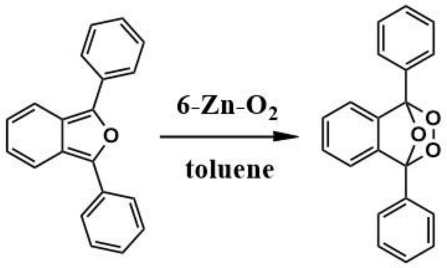

To confirm that some of the liberated oxygen is in its singlet state, the **6-Zn-O$_2$** was mixed with a toluene solution containing the $^1O_2$ trapping reagent 1,3-diphenylisobenzofuran (DPBF). For details, **6-Zn-O$_2$** (6.39 mg, 0.00129 mmol) and DPBF (2.76 mg, 0.0102 mmol) were dissolved in 2.7 mL of toluene and refluxed at 120°C, avoiding from lights. The UV-vis spectra of the solutions were monitored by pipetting 100 µL of this mixed solution at 0.5 h intervals and added into 2900 µL of toluene. As a control group, only 2.76 mg of DPBF (without **6-Zn-O$_2$**) was added under the same conditions and monitored the absorption spectra in the same way. The results showed that the addition of **6-Zn-O$_2$** for the same period of time resulted in a significant change in the absorbance of DPBF at 413 nm, due to the consumption of the trapping reagent by $^1O_2$ liberated by **6-Zn-O$_2$**.

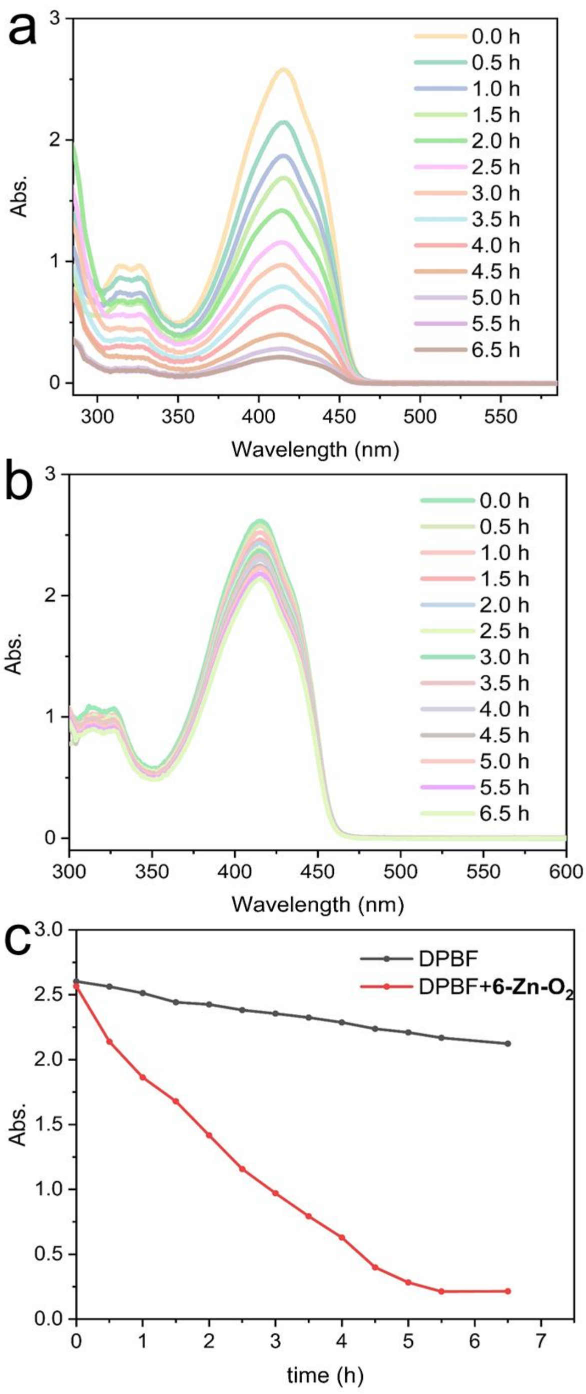

Supplementary Figure 45. Absorption spectra of a) the reaction solution of DPBF with $^1O_2$ generated upon the thermolysis of the **6-Zn-O$_2$**; b) solely DPBF blank control under the same condition; c) absorbance value at 413 nm of DPBF by capturing $^1O_2$ and blanks.

6. Photophysical properties

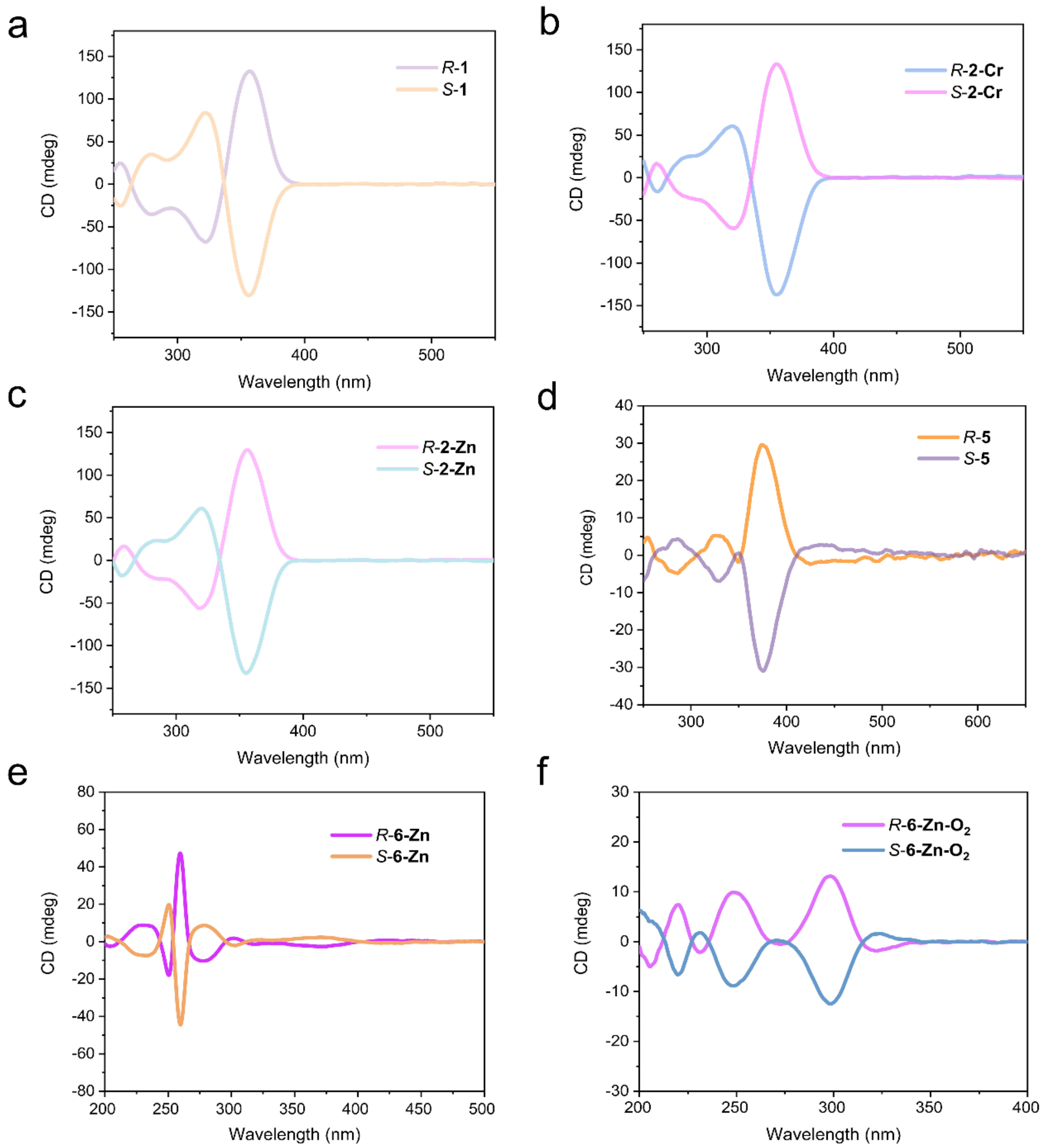

Supplementary Figure 46. The circular dichroism (CD) spectra of a) **1** (0.011 μM), b) **2-Cr** (0.013 μM), c) **2-Zn** (0.013 μM), d) **5** (0.014 μM), e) **6-Zn** (0.010 μM) and f) **6-Zn-O$_2$** (0.010 μM) in acetonitrile solution.

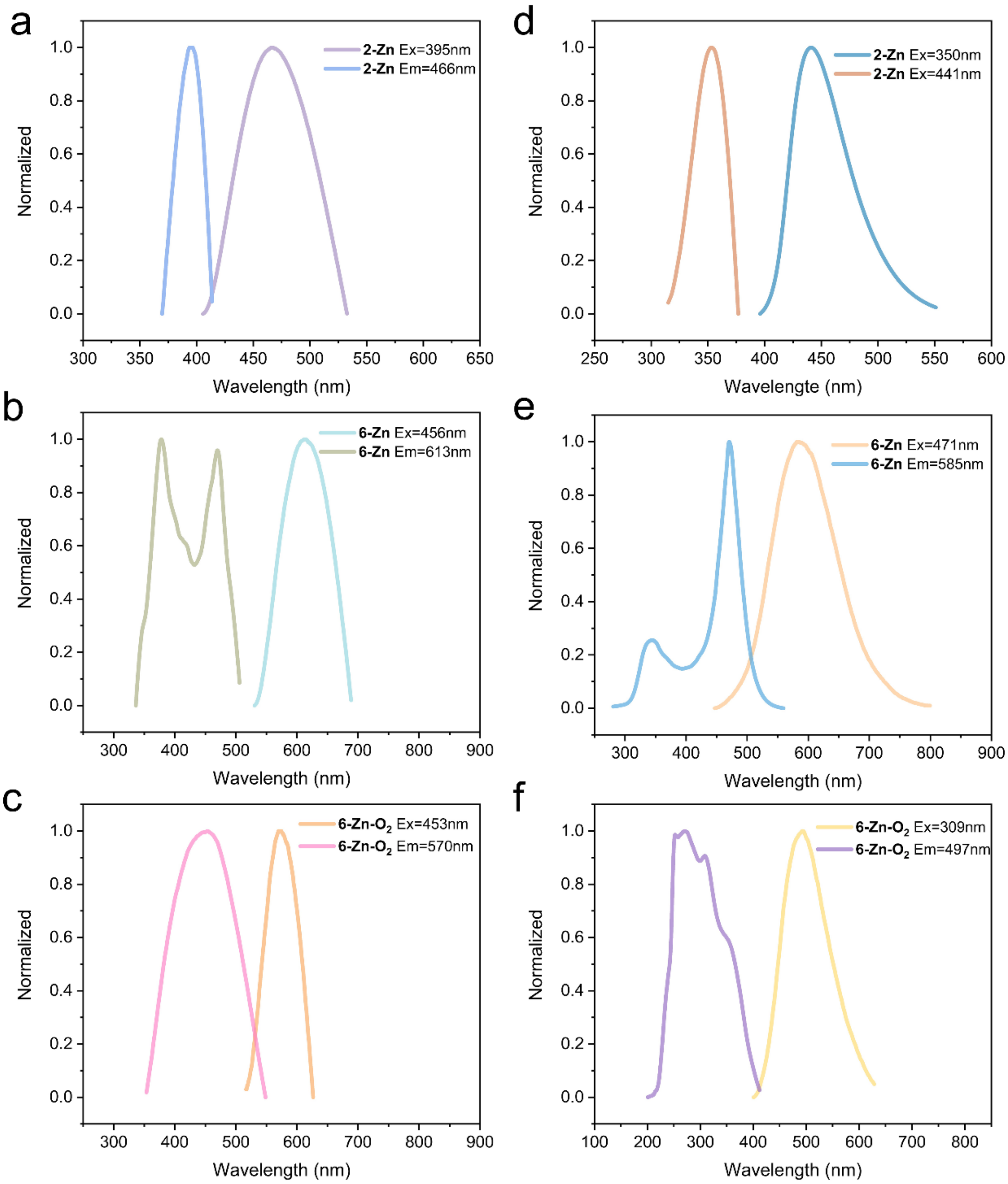

Supplementary Figure 47. The normalized excitation and emission spectra of a) **2**, b) **6-Zn** and c) **6-Zn-O_2** in solid state; and the normalized excitation and emission spectra of d) **2**, e) **6-Zn** and f) **6-Zn-O_2** in acetonitrile solution.

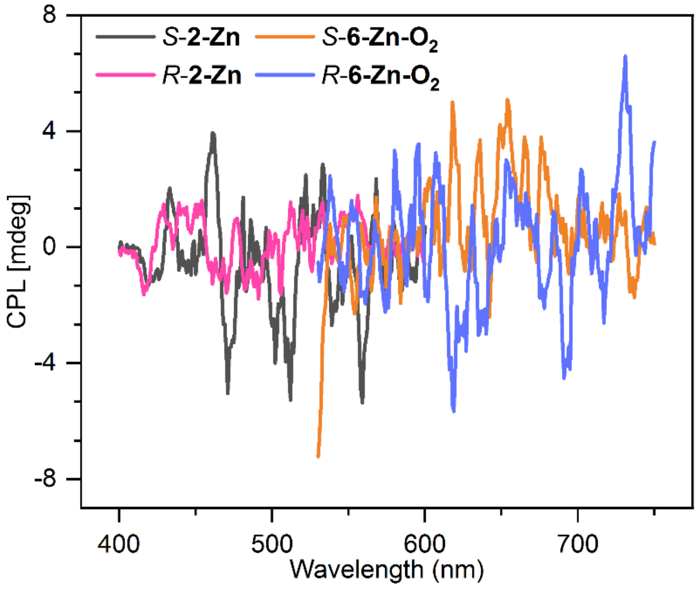

Supplementary Figure 48. The CPL spectra of *S/R*-**2-Zn** and *S*/*R*-**6-Zn-O$_2$** in solid state.

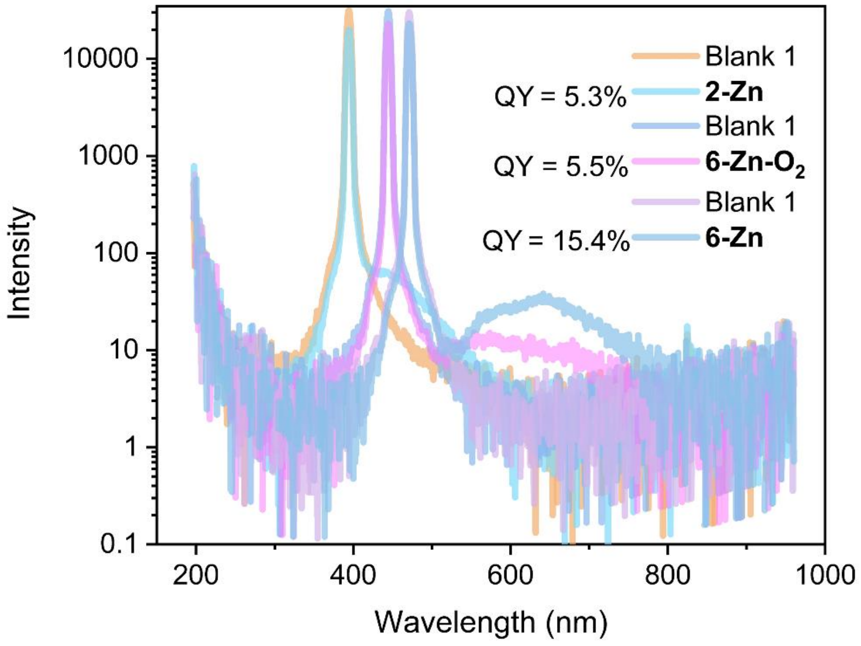

Supplementary Figure 49. Quantum efficiency measurements of crystalline **2-Zn** (QY = 5.3%), **6-Zn-O$_2$** (QY = 5.5%) and **6-Zn** (QY = 15.4%).

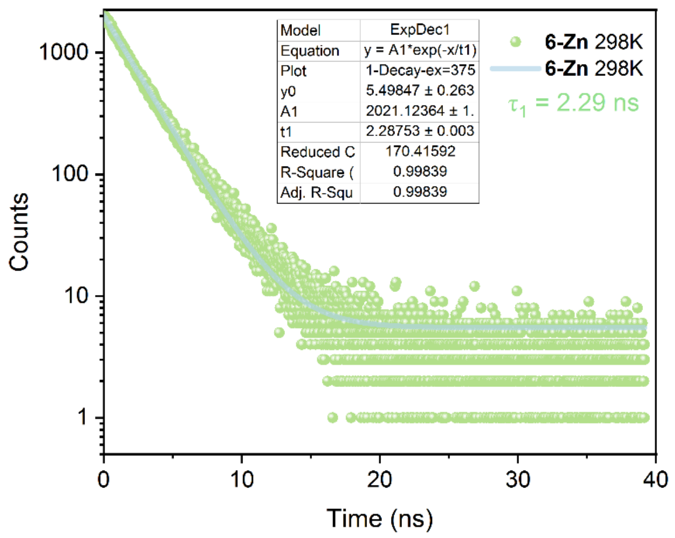

Supplementary Figure 50. Emission lifetimes of acetonitrile solution of **6-Zn** at room temperature (298 K). It is monoexponential decay, which indicates single species in **6-Zn**.

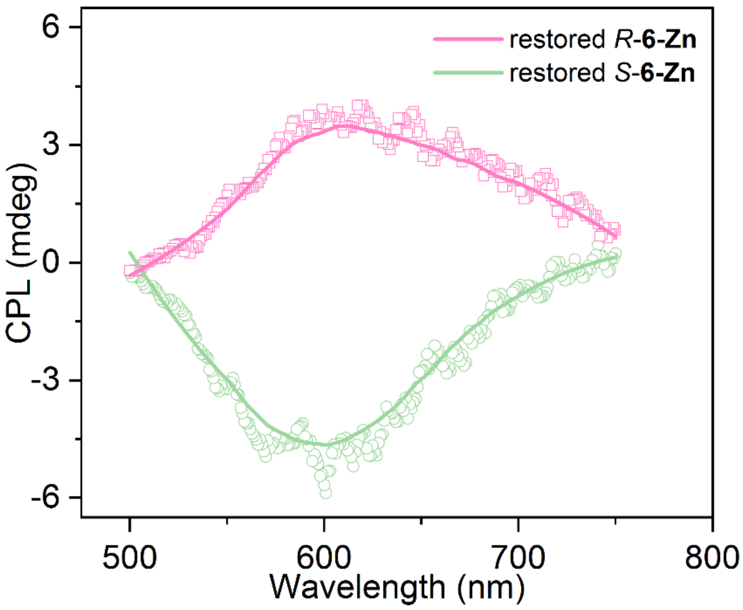

Supplementary Figure 51. The solid state CPL spectra of *S*/*R*-**6-Zn** restored by heating *S*/*R*-**6-Zn-O$_2$**.

7. Crystallographic data and structure refinement

The crystals were fragile and easily lost solvent molecules. Thus low temperature and rapid handing of the samples was needed. Diffraction data of *R*-**1**, *R*-**2**-Cr and *S*-**6-Co** were collected on Bruker SMART APEX II X-Ray diffractometer at 150 K using Mo-Kα (λ=0.71073 Å) X-ray sources. Data were processed with the INTEGRATE program of the APEX2 software for reduction and cell refinement. Multi-scan absorption corrections were applied by using the SCALE program for area detector. Diffraction data of *S*-**5** was collected on a Rigaku XtaLAB Synergy (DW system, HyPix) X-Ray diffractometer at 100 K using micro-focus X-ray sources (Cu Kα, λ=1.54184 Å). Data were processed with *CrysAlisPro* software suite. Empirical absorption correction was done by using spherical harmonics, implemented in SCALE3 ABSPACK scaling algorithm. Data of *S*-**6-Zn-O$_{12}$** were recorded on Bruker D8 VENTURE diffractometer with an Incoatec IμS 3.0 Cu EF microfocus source (55W, Cu Kα, λ = 1.54178 Å) equipped with a PHOTON III C28 detector at 100 K. The raw frame data were processed using SAINT and SADABS to yield the reflection data file.

Using Olex2,[3] the structure was solved with the ShelXT structure solution program using Intrinsic Phasing and refined with the ShelXL refinement package using Least Squares minimization.[4] In general, non-hydrogen atoms with occupancies greater than 0.5 were refined anisotropically. Carbon-bound hydrogen atoms were included in idealized positions and refined using a riding model. Crystallographic data have been deposited with the CCDC: 2307653 (*R*-**1**), 2307651 (*R*-**2-Cr**), 2307652 (*S*-**5**), 2112669 (*S*-**6-Co**) and 2307654 (*S*-**6-Zn-O$_2$**).

R-**1**: $[Zn_4(\mathbf{L^1})_6]\cdot 8ClO_4\cdot[+ 60CH_3CN]$

Formula: $C_{324}H_{357}Cl_8N_{84}O_{32}Zn_4$, M = 6485.06, a = 30.074(4) Å, b = 30.074(4) (15) Å, c = 30.074(4) Å, α = 90°, β = 90°, γ = 90°, V = 27200(9) Å^3, Cubic space group $P2_13$, Z = 3.99996, T = 150 K, 42503 reflections measured, 5506 unique (Rint = 0.2424), final $^*R1$ = 0.0922, w$R2$ = 0.2381 for 2998 observed reflections [$I > 2\sigma(I)$], flack factor 0.109(19).

$^*R1 = \Sigma||F_o| - |F_c||/\Sigma|F_o|$ for $F_o > 2\sigma(F_o)$; $wR2 = (\Sigma w(F_o^2 - F_c^2)^2/\Sigma(wF_c^2)^2)^{1/2}$ all reflections

$w=1/[\sigma^2(F_o^2)+(0.2000P)^2+200.0000P]$ where $P=(F_o^2+2F_c^2)/3$

Specific refinement details:

The crystals of *R*-**1** were grown by layering of diethyl ether with an acetonitrile solution of the complex in a thin tube. The crystals employed immediately lost solvent after removal from the mother liquor and rapid handling prior to flash cooling in liquid nitrogen was required to collect data. Despite these measures, the diffraction intensity was still relatively weak. Few reflections at greater than 1.2 Å resolution were observed and the data were trimmed accordingly. Nevertheless, the quality of the data is far more than sufficient to establish the connectivity of the structure. The asymmetric unit was found to contain one-third Zn_4L_6 assembly. Some restraints (including DFIX, FLAT, RIGU, and SIMU) were applied to areas of the structure showing higher levels of thermal motion. The structure was examined using the Addsym function of PLATON[5] to ensure that no additional symmetry could be applied to the models. Further reflecting the solvent loss and poor diffraction properties there is a substantial amount of void volume in the lattice containing smeared electron density from disordered solvent and undefined counterions. Consequently, the SQUEEZE[6] function of PLATON[5] was employed to remove the contribution of the electron density associated with these highly disordered solvents and counterions, which gave a potential solvent-accessible void of 13315 Å^3 per unit cell (a total of approximately 6845 electrons). The amount of acetonitrile solvent residues (60 acetonitrile molecules per cage) was estimated by the electron count and void volumes suggested by SQUEEZE, by assuming that any Squeezed moieties other than counterions indicated by ESI-MS are acetonitrile solvent residues. The correct chemical formula reported in CIF took the undefined acetonitrile solvent and counter-ions into consideration. The configuration of the crystal was supported by the solution CD studies of bulk samples, which showed active signals. Furthermore, chiral amines used for synthesis of the cages are enantiopure and with known exact handedness, which also are consistent with absolute structures as determined by crystallography. Thus, we believe there is no inversion twinning. CheckCIF gives an A level alert and 5 B level alerts, resulting from the limited resolution of the data.

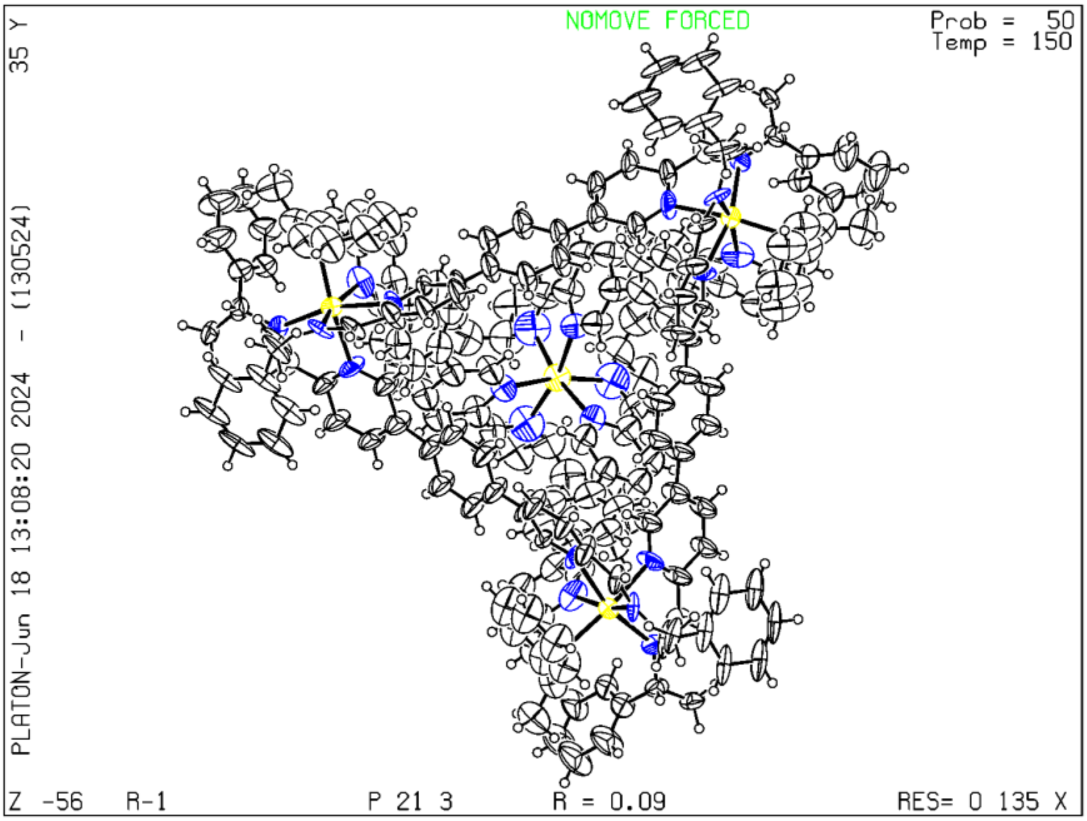

Supplementary Figure 52. ORTEP-style illustration of *R*-**1** (CCDC: 2307653) with 50% probability ellipsoids.

R-**2-Cr**: $\{[Cr^{III}(ox)_3]\subset Zn^{II}_5(\mathbf{L^1})_6\}\cdot 7ClO_4\cdot[+\ 50CH_3CN]$

Formula: $C_{310}H_{330}Cl_7CrN_{74}O_{40}Zn_5$, M = 6359.52, a = 20.8670(15) Å, b = 20.8670(15) Å, c = 58.316(4) (7) Å, α = 90°, β = 90°, γ = 120°, V = 21991(4) Å^3, trigonal space group $R3$, Z = 3, T = 150 K, 33244 reflections measured, 7961 unique (Rint = 0.0560), final $^*R1$ = 0.0845, w$R2$ = 0.2279 for 5638 observed reflections [$I > 2\sigma(I)$], flack factor 0.110(11).

$^*R1 = \Sigma||F_o| - |F_c||/\Sigma|F_o|$ for $F_o > 2\sigma(F_o)$; $wR2 = (\Sigma w(F_o^2 - F_c^2)^2/\Sigma(wF_c^2)^2)^{1/2}$ all reflections

$w=1/[\sigma^2(F_o^2)+(0.2000P)^2+200.0000P]$ where $P=(F_o^2+2F_c^2)/3$

Specific refinement details:

The crystals of *R*-**2-Cr** were grown by layering of diethyl ether with an acetonitrile solution of the complex in a thin tube. The crystals employed immediately lost solvent after removal from the mother liquor and rapid handling prior to flash cooling in liquid nitrogen was required to collect data. Despite these measures, the diffraction intensity was still relatively weak. Few reflections at greater than 1.09 Å resolution were observed and the data were trimmed accordingly. Nevertheless, the quality of the data is far more than sufficient to establish the connectivity of the structure. The asymmetric unit was found to contain one-third $Cr \subset Zn_5L_6$ assembly. Some restraints (including DFIX, FLAT, RIGU, and SIMU) were applied to areas of the structure showing higher levels of thermal motion. The structure was examined using the Addsym function of PLATON[5] to ensure that no additional symmetry could be applied to the models. Further reflecting the solvent loss and poor diffraction properties there is a substantial amount of void volume in the lattice containing smeared electron density from disordered solvent and undefined counterions. Consequently, the SQUEEZE[6] function of PLATON[5] was employed to remove the contribution of the electron density associated with these highly disordered solvents and counterions, which gave a potential solvent-accessible void of 10572 Å^3 per unit cell (a total of approximately 4373 electrons). The amount of acetonitrile solvent residues (50 acetonitrile molecules per cage) was estimated by the electron count and void volumes suggested by SQUEEZE, by assuming that any Squeezed moieties other than counterions indicated by ESI-MS are acetonitrile solvent residues. The correct chemical formula reported in CIF took the undefined acetonitrile solvent and counter-ions into consideration. The configuration of the crystal was supported by the solution CD studies of bulk samples, which showed active signals. Furthermore, chiral amines used for synthesis of the cages are enantiopure and with known exact handedness, which also are consistent with absolute structures as determined by crystallography. Thus, we believe there is no inversion twinning. CheckCIF gives an A level alert and 3 B level alerts, resulting from the limited resolution of the data.

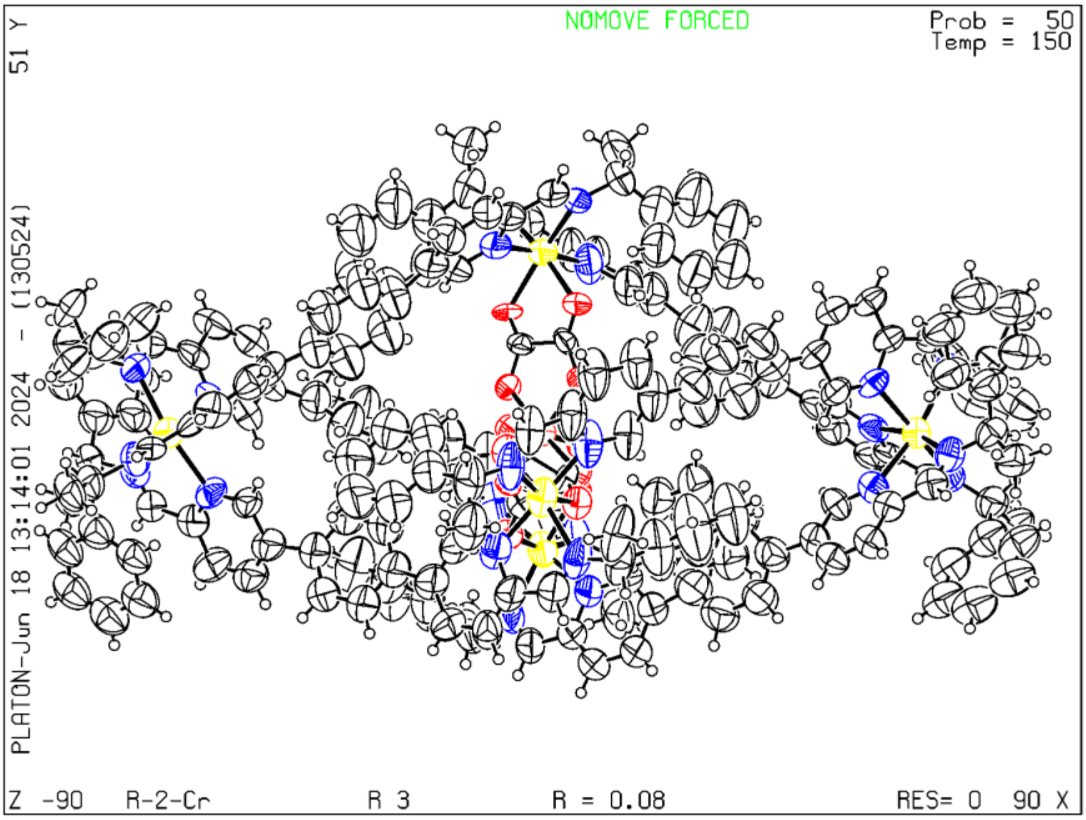

Supplementary Figure 53. ORTEP-style illustration of *R*-**2-Cr** (CCDC: 2307651) with 50% probability ellipsoids.

S-**5**: {[Co^{II}(chl)$_3$]⊂Co$^{II}_5$(**L**^{3})$_6$·6ClO$_4$·[+ 58CH$_3$CN]

Formula: $C_{374}H_{378}Cl_{12}Co_6N_{82}O_{36}$, M = 7376.54, a = 20.6366 Å (2), b = 20.6366(2) Å, c = 77.9720(9) Å, α = 90°, β = 90°, γ = 120°, V = 28757.1(6) Å^3, trigonal space group *R*32, Z = 3, T = 100.01(10) K, 39814 reflections measured, 12616 unique (*R*int = 0.0380), final $^*R1$ = 0.0578, w*R*2 = 0.1603 for 10552 observed reflections [$I > 2\sigma(I)$], flack factor -0.019(3).

$^*R1 = \Sigma||F_o| - |F_c||/\Sigma|F_o|$ for $F_o > 2\sigma(F_o)$; $wR2 = (\Sigma w(F_o^2 - F_c^2)^2/\Sigma(wF_c^2)^2)^{1/2}$ all reflections

$w=1/[\sigma^2(F_o^2)+(0.2000P)^2+200.0000P]$ where $P=(F_o^2+2F_c^2)/3$

Specific refinement details:

The crystals of *S*-**5** were grown by layering of diethyl ether with an acetonitrile solution of the complex in a thin tube. The crystals employed immediately lost solvent after removal from the mother liquor and rapid handling prior to flash cooling in liquid nitrogen was required to collect data. The asymmetric unit was found to contain one-sixth $Co\subset Co_5L_6$ assembly. Some restraints (including DFIX, SADI , SIMU and ISOR) were applied to areas of the structure showing higher levels of thermal motion. The structure was examined using the Addsym function of PLATON[5] to ensure that no additional symmetry could be applied to the models. Further reflecting the solvent loss, there is a substantial amount of void volume in the lattice containing smeared electron density from disordered solvent and undefined counterions. Consequently, the SQUEEZE[6] function of PLATON[5] was employed to remove the contribution of the electron density associated with these highly disordered solvents and counterions, which gave a potential solvent-accessible void of 10236 $Å^3$ per unit cell (a total of approximately 3120 electrons). 20 acetonitrile molecules per cage were molded and refined. The amount of unidentified acetonitrile solvent residues (38 acetonitrile molecules per cage) was estimated by the electron count and void volumes suggested by SQUEEZE, by assuming that any Squeezed moieties other than counterions indicated by ESI-MS are acetonitrile solvent residues. The correct chemical formula reported in CIF took the undefined acetonitrile solvent and counter-ions into consideration.

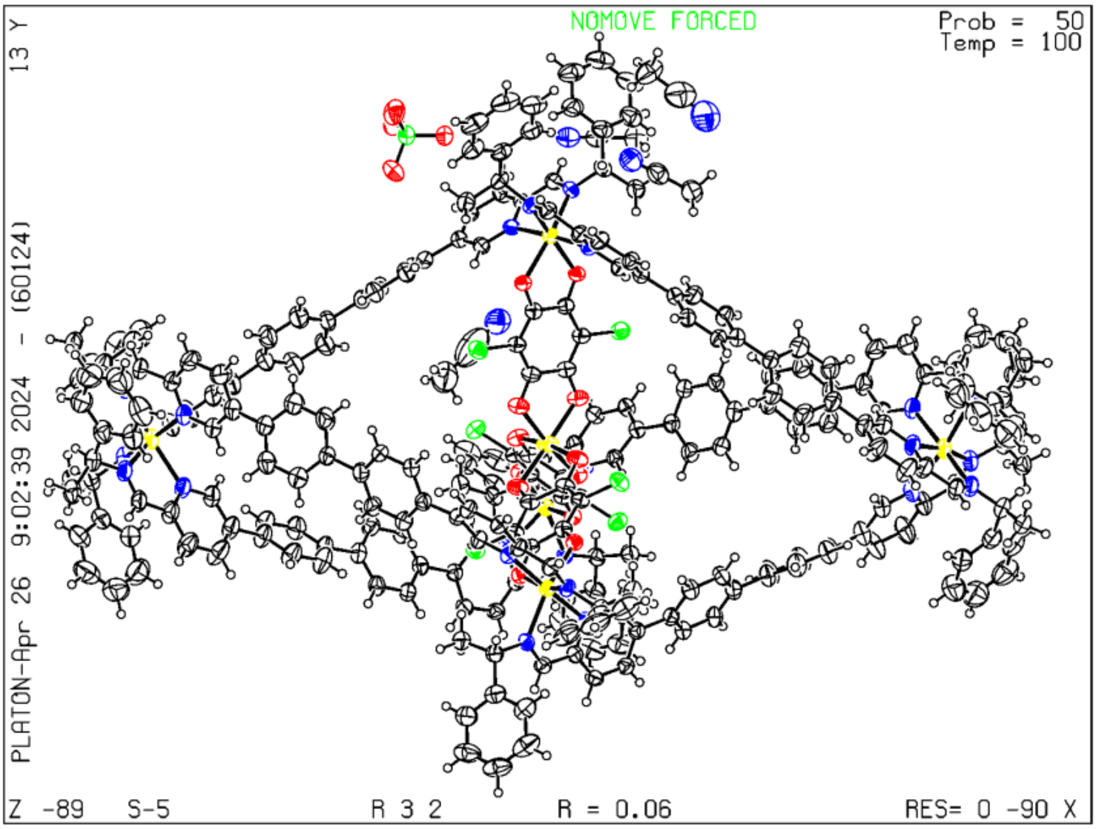

Supplementary Figure 54. ORTEP-style illustration of *S*-**5** (CCDC: 2307652) with 50% probability ellipsoids.

S-**6-Co**: {[2CH$_3$CN·Co(C$_2$O$_4$)$_3$]⊂*S*-Co$_5$**L**$^4{}_6$}·6ClO$_4$ [+ 30 CH$_3$CN]

Formula: $C_{322}H_{300}Cl_6Co_6N_{56}O_{36}$, M = 6096.44, a = 42.380(5) Å, b = 42.380 Å, c = 59.217(7) Å, α = 90°, β = 90°, γ = 120°, V = 92107(26) Å^3, trigonal space group $R3$, Z = 12, T = 150(2) K, 105559 reflections measured, 41212 unique (Rint = 0.0651), final $^*R1$ = 0.0664, w$R2$ = 0.1678 for 25017 observed reflections [$I > 2\sigma(I)$], flack factor 0.060(7).

$^*R1 = \Sigma||F_o| - |F_c||/\Sigma|F_o|$ for $F_o > 2\sigma(F_o)$; $wR2 = (\Sigma w(F_o^2 - F_c^2)^2/\Sigma(wF_c^2)^2)^{1/2}$ all reflections

$w=1/[\sigma^2(F_o^2)+(0.2000P)^2+200.0000P]$ where $P=(F_o^2+2F_c^2)/3$

Specific refinement details:

The crystals of *S*-**6-Co** were grown by layering of diethyl ether with an acetonitrile solution of the complex in a thin tube. The crystals employed immediately lost solvent after removal from the mother liquor and rapid handling prior to flash cooling in liquid nitrogen was required to collect data. Despite these measures, the diffraction intensity was still relatively weak. Few reflections at greater than 0.99 Å resolution were observed and the data were trimmed accordingly. Nevertheless, the quality of the data is far more than sufficient to establish the connectivity of the structure. The asymmetric unit was found to contain one and one-third Co_5L_6 assemblies and associated counterions and guests. Some restraints (including DFIX, RIGU, FLAT and ISOR) were applied to areas of the structure showing higher levels of thermal motion. The structure was examined using the Addsym function of PLATON[5] to ensure that no additional symmetry could be applied to the models. Further reflecting the solvent loss and poor diffraction properties there is a substantial amount of void volume in the lattice containing smeared electron density from disordered solvent and undefined counterions. Consequently, the SQUEEZE[6] function of PLATON[5] was employed to remove the contribution of the electron density associated with these highly disordered solvents and counterions, which gave a potential solvent-accessible void of 34549 Å^3 per unit cell (a total of approximately 10038 electrons). 2 acetonitrile molecules inside the cage were molded and refined. The amount of unidentified acetonitrile solvent residues (30 acetonitrile molecules per cage) was estimated by the electron count and void volumes suggested by SQUEEZE, by assuming that any Squeezed moieties other than counterions indicated by ESI-MS are acetonitrile solvent residues. The correct chemical formula reported in CIF took the undefined acetonitrile solvent and counter-ions into consideration. The configuration of the crystal was supported by the solution CD studies of bulk samples, which showed active signals. Furthermore, chiral amines used for synthesis of the cages are enantiopure and with known exact handedness, which also are consistent with absolute structures as determined by crystallography. Thus, we believe there is no inversion twinning. CheckCIF gives an A level alert and 2 B level alerts, resulting from the limited resolution of the

data.

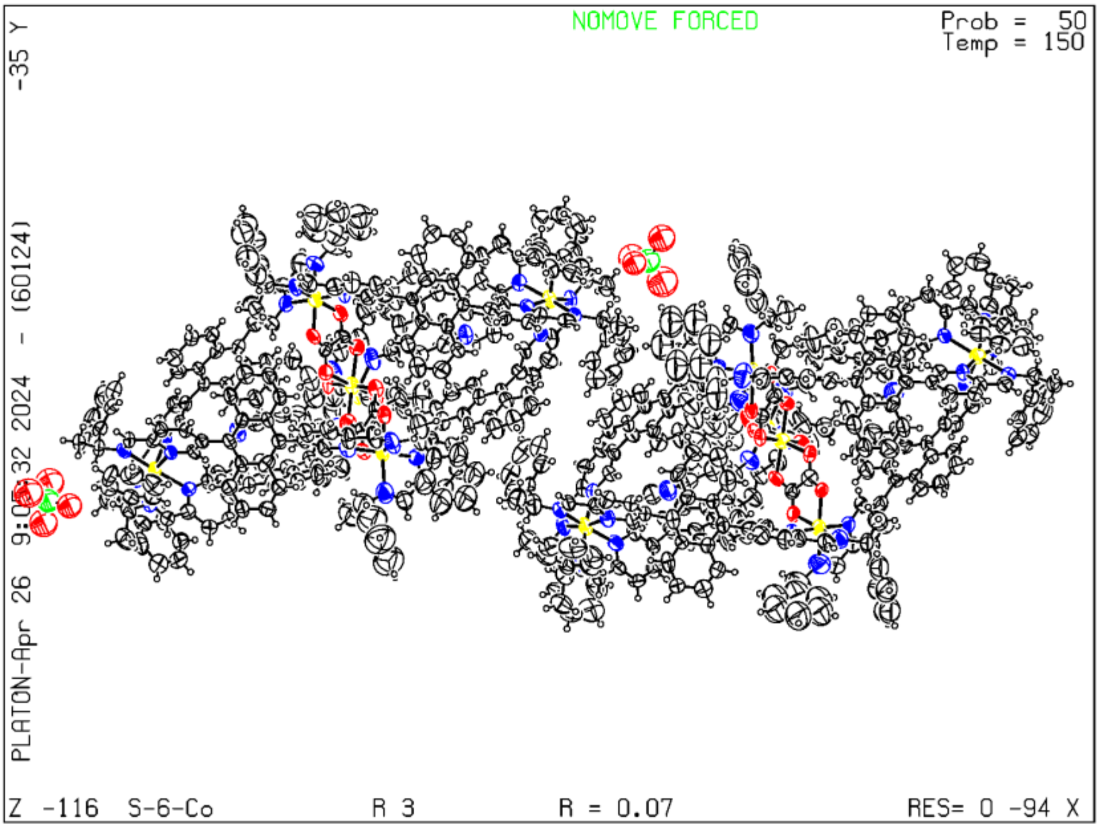

Supplementary Figure 55. ORTEP-style illustration of *S*-**6-Co** (CCDC: 2112669) with 50% probability ellipsoids.

S-**6-Zn-O**$_2$: {[Zn(C_2O_4)$_3$]⊂*S*-Zn$_5$**L**$^4{}_6$(O_2)$_6$}·6NTf$_2$ [+ 51 CH_3CN]

Formula: $C_{364}H_{357}F_{12}N_{77}O_{32}S_4Zn_6$, M = 7070.71, a = 24.8711(9) Å, b = 30.9077(10) Å, c = 52.9994(18) Å, α = 90°, β = 90°, γ = 90°, V = 40741(2) Å^3, Orthorhombic space group $P2_12_12_1$, Z = 4, T = 100.0 K, 254202 reflections measured, 71769 unique (Rint = 0.0856), final $^*R1$ = 0.0662, w$R2$ = 0.1786 for 33324 observed reflections [$I > 2\sigma(I)$], flack factor 0.156(9).

$^*R1 = \Sigma||F_o| - |F_c||/\Sigma|F_o|$ for $F_o > 2\sigma(F_o)$; $wR2 = (\Sigma w(F_o{}^2 - F_c{}^2)^2/\Sigma(wF_c{}^2)^2)^{1/2}$ all reflections

w=1/[$\sigma^2(F_o{}^2)$+(0.2000P)^2+200.0000P] where P=($F_o{}^2$+2$F_c{}^2$)/3

Specific refinement details:

The crystals of ***S*-6-Zn-O$_2$** (NTf_2^- ions were the counterions, which were obtained by using $Zn(NTf_2)_2$, i.e. zinc bis(trifluoromethylsulfonyl)imide, instead of $Zn(BF_4)_2$ in the synthesis) were grown by layering of diethyl ether with an acetonitrile solution of the complex in a thin tube. The crystals employed immediately lost solvent after removal from the mother liquor and rapid handling prior to flash cooling in liquid nitrogen was required to collect data. Despite these measures, the diffraction intensity was still relatively weak. Few reflections at greater than 0.84 Å resolution were observed and the data were trimmed accordingly. Nevertheless, the quality of the data is far more than sufficient to establish the connectivity of the structure. The asymmetric unit was found to contain one whole Co_5L_6 assemblies and associated counterions. Some restraints (including DFIX, SADI, RIGU, FLAT and SIMU) were applied to areas of the structure showing higher levels of thermal motion. The thermal parameters of phenyl rings C140, C188, C285, and C236 exceed those of similar phenyl groups in the structure, which exhibit more typical values. We attempted to consider these phenyl rings as disordered at half occupancy, or any other occupation values, but were unsuccessful in identifying suitable alternate half occupancies. Any attempts to model an alternative position for these rings result in configurations with significantly large thermal parameters, despite the application of strict restraints. We found that the phenyl groups with normal thermal parameters all form obviously π-π interactions with adjacent ligands, while the ones with large thermal parameters are on the tail of the ligands and able to rotate without forming any intra-molecular or inter-molecular interactions with neighbors. Thus, it is reasonable for these phenyl groups to have larger thermal parameters than chemically similar phenyl moieties. The structure was examined using the Addsym function of PLATON[5] to ensure that no additional symmetry could be applied to the models. Further reflecting the solvent loss and poor diffraction properties there is a substantial amount of void volume in the lattice containing smeared electron density from disordered solvent and undefined counterions. Consequently, the SQUEEZE[6] function of PLATON[5] was employed to remove the contribution of the electron density associated with these highly disordered solvents and counterions, which gave a potential solvent-accessible void of 21257 Å^3 per unit cell (a total of approximately 6559 electrons). The amount of acetonitrile solvent residues (51 acetonitrile molecules per cage) was estimated by the electron count and void volumes

suggested by SQUEEZE, by assuming that any Squeezed moieties other than counterions indicated by ESI-MS are acetonitrile solvent residues. The correct chemical formula reported in CIF took the undefined acetonitrile solvent and counter-ions into consideration. The configuration of the crystal was supported by the solution CD studies of bulk samples, which showed active signals. Furthermore, chiral amines used for synthesis of the cages are enantiopure and with known exact handedness, which also are consistent with absolute structures as determined by crystallography. Thus, we believe there is no inversion twinning. CheckCIF gives B level alerts, resulting from the limited resolution of the data and high levels of thermal motion of phenyl rings at the waist of the cage.

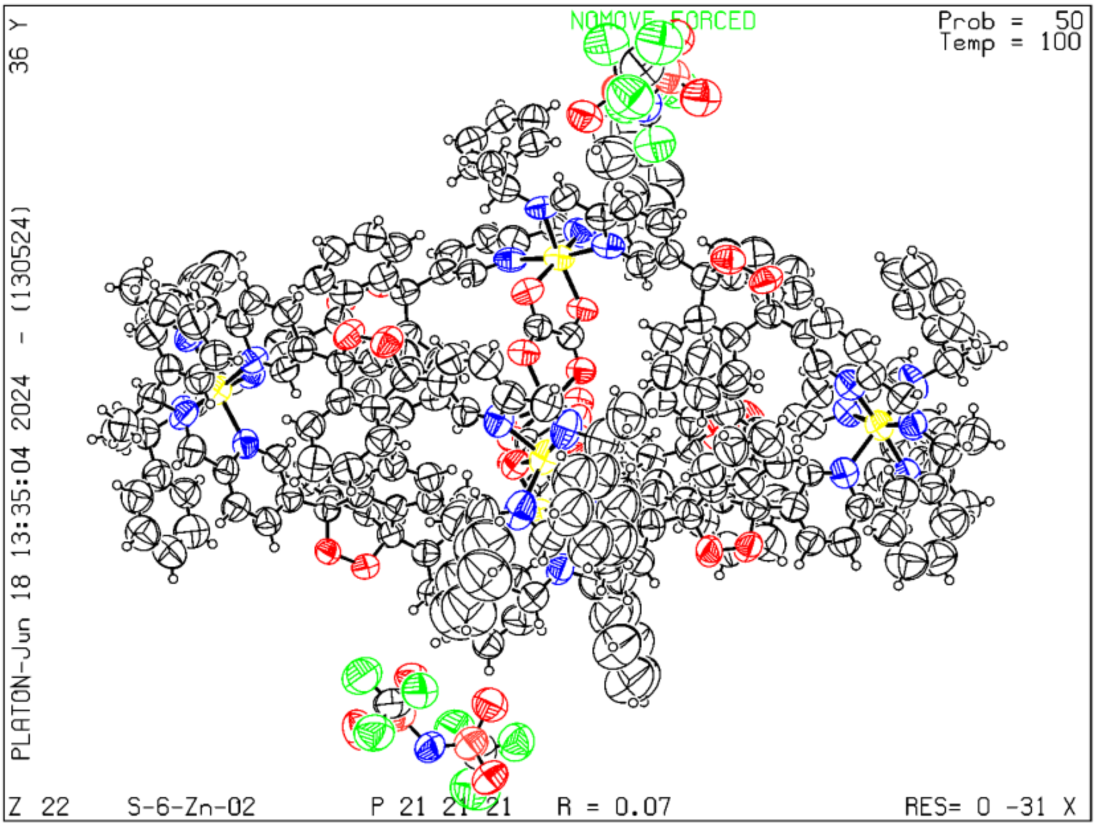

Supplementary Figure 56. ORTEP-style illustration of *S*-**6-Zn-O$_2$** (CCDC: 2307654) with 50% probability ellipsoids.

Supplementary References:

1. Knighton R. C., *et al.* Upconversion in a d–f [$RuYb_3$] Supramolecular Assembly. *J Am Chem Soc* **144**, 13356-13365 (2022).

2. Omoto K., Tashiro S., Shionoya M. Phase-Dependent Reactivity and Host–Guest Behaviors of a Metallo-Macrocycle in Liquid and Solid-State Photosensitized Oxygenation Reactions. *J Am Chem Soc* **143**, 5406-5412 (2021).

3. Dolomanov O. V., Bourhis L. J., Gildea R. J., Howard J. A. K., Puschmann H. OLEX2: a complete structure solution, refinement and analysis program. *J Appl Crystallogr* **42**, 339-341 (2009).

4. Sheldrick G. SHELXT - Integrated space-group and crystal-structure determination. *Acta Crystallogr, Sect A* **71**, 3-8 (2015).

5. Spek A. L. *PLATON: A Multipurpose Crystallographic Tool*. Utrecht University (2008).

6. van der Sluis P., Spek A. L. BYPASS: an effective method for the refinement of crystal structures containing disordered solvent regions. *Acta Cryst* **A46**, 194-201 (1990).
