# Supplementary material for: Enantiopure trigonal bipyramidal coordination cages templated by in situ self-organized D2h-symmetric anions
Source: Nat Commun. 2024 Jul 4;15:5628. doi: 10.1038/s41467-024-49964-w (PMC11224320; doi:10.1038/s41467-024-49964-w)
Supplement: Supplementary file 3 — Description of Additional Supplementary Files [file 41467_2024_49964_MOESM3_ESM.pdf]

Description of Additional Supplementary Files

File Name: Supplementary Data 1
Description: Atomic coordinates of the optimized computational models
